# Time-updated patterns of hemoglobin and hematocrit and the risk of CKD progression

**DOI:** 10.3389/fendo.2025.1642307

**Published:** 2025-10-30

**Authors:** Li-zhe Fu, Hui-fen Chen, Yu-han Shen, Xian-long Zhang, Fang Tang, Xiao-xuan Hu, Zhen-jie Liu, Wen-wei Ouyang, Xu-sheng Liu, Yi-fan Wu

**Affiliations:** ^1^ The Second Clinical College of Guangzhou University of Chinese Medicine, Guangzhou, Guangdong, China; Chinese Medicine Guangdong Laboratory, Guangzhou, Guangdong, China; ^2^ Department of Chronic Disease Management, The Second Affiliated Hospital of Guangzhou University of Chinese Medicine (Guangdong Provincial Hospital of Chinese Medicine), Guangzhou, Guangdong, China; ^3^ The Second Clinical College/School of Nursing, Guangzhou University of Chinese Medicine, Guangzhou, Guangdong, China; Chinese Medicine Guangdong Laboratory, Guangdong Hengqin, China; ^4^ Department of Nephrology, The Second Affiliated Hospital of Guangzhou University of Chinese Medicine (Guangdong Provincial Hospital of Chinese Medicine), Guangzhou, Guangdong, China; ^5^ Department of Clinical Laboratory, The Second Affiliated Hospital of Guangzhou University of Chinese Medicine (Guangdong Provincial Hospital of Chinese Medicine), Guangzhou, China; ^6^ Key Unit of Methodology in Clinical Research, The Second Affiliated Hospital of Guangzhou University of Chinese Medicine (Guangdong Provincial Hospital of Chinese Medicine), Guangzhou, China; ^7^ Global Health - Health Systems and Policy, Department of Global Public Health, Karolinska Institute, Stockholm, Sweden

**Keywords:** chronic kidney disease, anemia, non-dialysis, trajectories, variability

## Abstract

**Background:**

Early intervention and management of anemia, particularly the commonly used measures of hemoglobin (Hb) and hematocrit (HCT), are important in slowing and preventing the progression of chronic kidney disease (CKD). However, the optimal range for regulating Hb and HCT levels remains uncertain.

**Objective:**

The aim of this study was to elucidate the intrinsic relationship between Hb and HCT and the short- and long-term prognosis of CKD, and determine optimal ranges for Hb and HCT.

**Methods:**

We retrospectively collected demographic and clinical data over a 6-year follow-up period in Lingnan, China, to show the long-term characteristics of Hb and HCT, and studied the association between Hb, HCT, and the prognosis of patients with CKD stages 3–4. We constructed Cox and group-based trajectory modeling (GBTM) models to examine Hb’s and HCT’s associations with short- and long-term risk of composite outcomes in patients with CKD stages 3–4.

**Results:**

A total of 730 individuals were included, with a median age of 59.30 (48.47, 68.63) years, 306 (41.92%) were women, and median eGFR was 39.24 (26.26, 50.67) mL/min/1.73 m^2^. A multivariate time-dependent Cox model revealed mean_Hb and mean_HCT as independent protective factors for the composite outcome {hazard ratio (HR) (95% confidence interval [CI]): 0.851 (0.786, 0.921) g/L, *p=*0.000; 0.578% (0.441%, 0.758%), *p=*0.000}. Optimized GBTM models categorized Hb and HCT into four groups. Group 1 (“lower and decreasing”) (Hb<100 g/L, HCT approximately 30%) served as the reference. Groups 2 (“lower and growing slightly”) (Hb 110–120 g/L, HCT approximately 35%), 3 (“higher and growing slightly”) (Hb 125–135 g/L, HCT approximately 40%), and 4 (“higher and growing steadily”) (Hb 145–160 g/L, HCT approximately 45%) served as independent protective factors for patients with CKD stages 3–4 for the composite outcome (*p=*0.000; *p* for trend<0.000). Subgroup analyses showed interactions between mean_Hb and sex (*p* for interaction=0.034), as well as between Hb trajectory group 2 and CKD stage (*p* for interaction=0.015).

**Conclusions:**

Maintenance of stable and higher Hb levels of 110–130 g/L and HCT levels of 35%–40% in patients with CKD stages 3–4 is both protective and reliable in delaying CKD progression.

## Introduction

Chronic kidney disease (CKD) is characterized by a progressive loss of renal structure and function, which may eventually lead to irreversible renal impairment. The global prevalence of adult CKD ranges from approximately 15% to 20%, with the prevalence of adult CKD in mainland China estimated at approximately 8.2% (1–3). Patients with end-stage renal disease (ESRD) must rely on renal replacement therapy (RRT), such as dialysis or renal transplantation, in order to continue to live. It is possible that those affected may experience a greater economic burden and a lower quality of life (4, 5). Therefore, the development of efficacious interventions to impede the progression of CKD has become a current research priority in the field of nephrology.

Anemia is a prevalent complication in patients with CKD, with a twofold higher prevalence than in the general population (6). Impaired renal function results in a microinflammatory state, decreased oxidative capacity, and abnormal endocrine function. Together, these factors lead to decreased erythropoietin (EPO) production and abnormal absorption and transport of hematopoietic raw materials such as iron. Research indicates that anemia has a detrimental impact on the quality of life of patients with CKD. On the one hand, anemia is associated with adverse symptoms such as fatigue and depression. On the other hand, anemia increases the risk of entering ESRD and developing all-cause mortality (6–9). The prompt intervention and management of anemia is of significant importance in the slowing down and prevention of the progression of CKD.

Hemoglobin (Hb) and hematocrit (HCT) are commonly employed in the assessment and management of anemia, as they provide a direct reflection of the status and function of red blood cells. Hb is responsible for transporting oxygen and is directly related to the body’s oxygen supply, which is a key substance for maintaining normal physiological functions of the body. HCT is the proportion of red blood cells and is crucial for assessing the status of red blood cell production and destruction. Therefore, Hb and HCT play an important role in the diagnosis, treatment, and management of renal anemia. Hb and HCT can be effectively improved by conventional pharmacological means of treatment, including EPO, iron, and folic acid, as well as the latest drugs such as roxadustat (10, 11).

However, the optimal range for regulating levels of Hb and HCT remains uncertain, with conflicting recommendations present across guidelines. For instance, the 2012 the Kidney Disease: Improving Global Outcomes (KDIGO) guideline recommended that the Hb of patients with CKD should be controlled at 110–120 grams per liter (g/L), corresponding to an HCT of 33%–36%. In contrast, the 2017 UK NICE guideline recommended controlling the level of Hb of adults at 100–120 g/L. Researchers in mainland China proposed that the optimal therapeutic target for Hb in patients with CKD should be within the range of 115–130 g/L. It was further recommended that Hb levels should be individually adjusted within the range of 110–120 g/L in accordance with the patient’s chronological age, the mode and duration of dialysis, the duration of the erythropoiesis-stimulating agent (ESA) treatment, and the presence of other concomitant diseases (12–14). Furthermore, there are only a few guidelines that suggest a growth rate in Hb during anemia treatment. For instance, the Chinese expert consensus on renal anemia states that the initial goal of ESA treatment is to increase Hb by 10–20 g/L per month, and to avoid an increase in Hb of more than 20 g/L within 1 month (14). The controversy surrounding the Hb targets makes it challenging for physicians to identify a clear standard in the actual treatment process, which in turn increases the complexity and uncertainty of anemia management.

The optimal range for level of Hb is a matter of contention for a number of reasons. Firstly, the majority of studies only observe the relationship between baseline Hb level and disease prognosis, whereas in reality, the Hb concentration undergoes changes with the CKD progression. Focusing solely on a specific point in time is not sufficiently objective to analyze the relationship between anemia and disease prognosis (8, 9, 15). Furthermore, most studies on the prognosis of patients with CKD with anemia have employed stratified comparative analyses, which have yielded disparate conclusions, leading to heterogeneity in Hb control criteria (16, 17). Thirdly, a number of pertinent studies were conducted among Western populations, with fewer studies conducted in Asian populations (16, 17).

In light of the controversies previously outlined, our study investigates the relationship between baseline level and longitudinal change of Hb and HCT and CKD prognosis in Chinese patients with CKD stages 3–4 in order to elucidate the intrinsic link between Hb and HCT and CKD short- and long-term prognosis, as well as to determine the optimal range of Hb and HCT control.

## Methods

### Study design and population

We searched the Hospital Information System (HIS) at Guangdong Provincial Hospital of Chinese Medicine (GPHCM) with the following keywords: “kidney disease” or “renal failure” or “renal disease” or “nephritis” or “proteinuria” or “hematuria”. Patients with kidney disease who visited from March 2012 to March 2023 were enrolled in a single-center, retrospective cohort study (Ethics approval no. ZE2023-330). Patients aged 18–80 years with CKD stages 3–4 were eligible (18). Patients who had been diagnosed with acute and critical illnesses (e.g., acute cerebral infarction, acute heart failure, shock, malignant tumor, and hematological diseases) within 3 months of the baseline, those who had incomplete follow-up data, those who had received RRT within 3 months from the baseline, those with eGFR less than 15 mL/min/1.73m2, those with a follow-up period of less than 3 months, or those who had missing data for covariates exceeding 20% were excluded.

### Exposures

Hb and HCT were collected at each clinical visit. The baseline values, the mean values during the first year, and the trajectories during the follow-up period of Hb and HCT were considered as exposure variables. Trajectories were modeled using values aggregated in 3-month intervals.

### Covariates

All covariates, including demographic data, clinical diagnoses, medication in use, physical examination, and laboratory indicators, were fixed variables collected at baseline. The demographic characteristics included age (continuous), sex (fixed variables, male vs. female), and marital status. The clinical diagnosis included etiology and comorbidities. The etiology included primary glomerulopathy, hypertensive nephropathy, diabetic nephropathy, other secondary kidney diseases (including infectious or autoimmune kidney diseases, obstructive nephropathy, uric acid nephropathy, and pyelonephritis), and unknown. Comorbidities included hypertension, diabetes, hyperuricemia, hyperlipidemia, and anemia. Medication in use included ACEI/ARB, hypoglycemic agents, urate-lowering drugs, lipid-lowering drugs, calcium supplements, sodium bicarbonate, ketoacid tablets, diuretics, ESAs, or iron. Laboratory measurements were mostly obtained under standardized procedures at Guangdong Provincial Hospital of Chinese Medicine—morning venous sampling after an overnight fast. These measurements included serum albumin (ALB), creatinine (SCr), blood urea nitrogen (urea), total carbon dioxide (TCO_2_), uric acid (UA), aspartate aminotransferase (AST), alanine aminotransferase (ALT), phosphorus (P), calcium (Ca^2+^), potassium (K^+^), sodium (Na^+^), low-density lipoprotein cholesterol (LDL-C), total cholesterol (TC), high-density lipoprotein cholesterol (HDL-C), fasting blood glucose (Glu), and urine protein/creatinine ratio (UPCR). Covariates with a missing rate exceeding 30% were excluded, including P, Ca^2+^, K^+^, Na^+^, Glu, and UPCR (**Supplementary Data Sheet 1**). eGFR was calculated using the CKD Epidemiology Collaboration (CKD-EPI) creatinine equation.

### Outcomes

Composite outcome was defined as all-cause mortality, ESRD (received RRT or eGFR<15 mL/min/1.73 m^2^), at least a 50% decline in eGFR from baseline, and a doubling of SCr. Patients were followed until the end of the follow-up period or until they were lost to follow-up.

### Statistical analysis

Outliers of continuous variables were winsorized with 0.1 cutoffs at each tail. The corrplot R package was applied to analyze variable multicollinearity (**Supplementary Data Sheet 1**), and LASSO regression was used for features selection. Missing baseline covariates were imputed with multiple imputation through the Markov chain Monte Carlo sampling approach (with the mice R package) to reduce potential bias and the estimators were pooled based on Rubin’s 1987 criterion (19).

Continuous variables were summarized by mean ± standard deviation (SD) for normal distributions and by median (interquartile ranges) for non-normal distributions. Categorical variables were summarized by frequencies and percentages (%). Mann–Whitney *U*-test and chi-square test were applied to compare between-group characteristics. Tables and bar charts were employed to demonstrate and contrast the characteristics of the individuals included and the features of the groups classified in accordance with the Chinese guideline and consensus (14).

Exposure 1 (baseline_Hb and mean_Hb during the first year), exposure 2 (baseline_HCT and mean_HCT during the first year), and their trajectories via the group-based trajectory modeling (GBTM) method were considered as independent variables. The trajectories were extracted based on trajectory length, average velocity, direction, and other trajectory features and fitted in one to six trajectory subgroups with linear, square, and cubic forms. The best-fit model was selected by statistical criteria, model-fit indices, and specialized knowledge (**Supplementary Data Sheet 4**).

Kaplan–Meier curves with a log-rank test were employed to visualize survival probability. Univariate and multivariate Cox regression models were fitted with baseline_Hb and mean_Hb, baseline_HCT and mean_HCT, and the change trajectory groups of Hb and HCT as independent variables; the composite outcome and survival time as dependent variables; and the baseline values of other demographic and clinical information as covariates. Covariates with a *p*-value of less than 0.10 were added to construct multivariate Cox models with Lasso regression. *p* for trend was calculated to show whether there are any trends between trajectory subgroups.

### Subgroup analyses and sensitivity analyses

Subgroup analyses were performed based on the final multivariate Cox model for each dataset to explore the existing interaction of subgroups of age, sex, CKD stage, and comorbidity (hypertension and diabetes mellitus) with the composite outcome. The above analyses were repeated as sensitivity analyses using cases with complete baseline information.

Proportional hazard (PH) assumption was tested via Schoenfeld residuals. An interaction with time of follow-up was introduced if the PH assumption was violated. The results of the Cox regressions were expressed as hazard ratios (HRs) with 95% confidence intervals (CIs) and a forest plot was applied to visualize the results of the analysis. GBTM was conducted using SAS 9.4. The remaining analyses were performed using R Studio. All statistical tests were two-sided, and *p*-values < 0.05 were considered statistically significant.

## Results

### Baseline characteristics

**Figure 1:**
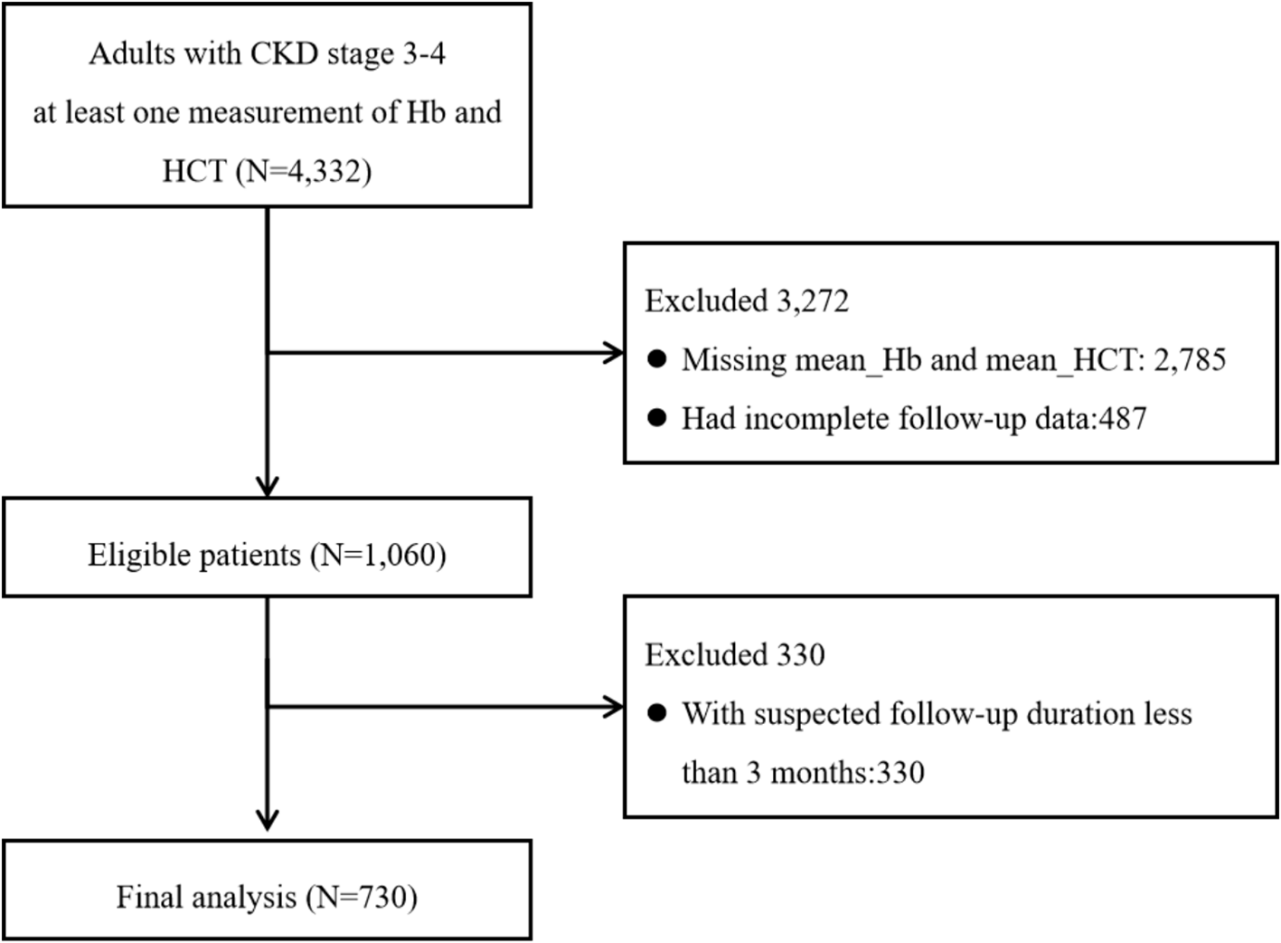
Patient selection flowchart.

We identified 4,332 adult patients with CKD stages 3–4 who had at least one Hb and HCT measurement. A total of 2,785 individuals had lack of exposures, 487 had incomplete follow-up data, and 330 had a follow-up duration of less than 3 months. A total of 730 individuals were included (**Figure 1**).

The median age of the 730 patients was 59.30 (48.47, 68.63) years with 306 (41.92%) women. The median eGFR was 39.24 (26.26, 50.67) mL/min/1.73 m^2^. A total of 204 (27.90%) patients were diagnosed with primary glomerulonephritis, 536 (73.42%) patients had a history of hypertension, 234 (32.05%) were diagnosed with diabetes mellitus, 33 (4.52%) had a history of anemia, and 299 (40.96%) reported the use of ACEI/ARB. The median level of baseline_Hb was 121.00 (107.00, 135.00) g/L and the median level of mean_Hb in the first year was 120.00 (105.81, 132.92) g/L. The median level of baseline_HCT was 36.80% (32.62%, 40.80%), and the median level of mean_HCT in the first year was 36.61% (32.27%, 40.19%) (**Table 1**).

**Table 1:** Baseline characteristics of the total sample and the grouped sample according to expert consensus.

Variables		Overall(N=730)	Substandard group (<115g/L) (N=270)	Standard group (≥115g/L, ≤130g/L) (N=219)	Excess group (>130g/L) (N=241)	*P*
		Mean (SD)/Median (IQR)/N(%)				
baseline_Hb, g/L		121.00 (107.00, 135.00)	102.00 (92.00, 109.00)	122.00 (118.00, 126.00)	140.00 (135.00, 150.00)	<0.001
mean_Hb, g/L		120.00 (105.81, 132.92)	104.25 (95.36, 111.20)	120.25 (113.33, 125.22)	139.60 (130.00, 148.17)	<0.001
baseline_HCT, %		36.80 (32.62, 40.80)	31.30 (28.32, 33.07)	37.20 (35.90, 38.45)	42.40 (40.80, 44.90)	<0.001
mean_HCT, %		36.61 (32.27, 40.19)	31.89 (29.11, 34.20)	36.65 (34.48, 38.18)	42.10 (39.26, 44.27)	<0.001
Age, year		59.30 (48.47, 68.63)	59.54 (49.24, 68.72)	60.83 (49.90, 68.90)	58.13 (47.01, 68.37)	0.324
Sex	Female	306 (41.92)	158 (58.52)	101 (46.12)	47 (19.50)	<0.001
	Male	424 (58.08)	112 (41.48)	118 (53.88)	194 (80.50)	
eGFR, ml/min/1.73 m2		39.24 (26.26, 50.67)	30.52 (22.34, 45.42)	39.45 (26.34, 50.47)	45.85 (34.56, 55.13)	<0.001
ALB, g/L		40.90 (35.90, 44.50)	37.40 (31.65, 41.45)	40.90 (36.50, 44.30)	44.10 (40.10, 46.50)	<0.001
Urea, mmol/L		9.19 (7.30, 11.92)	10.80 (8.42, 13.40)	9.33 (7.42, 11.50)	7.90 (6.44, 9.79)	<0.001
UA, mmol/L		454.85 (383.00, 529.00)	458.00 (379.00, 543.00)	451.00 (376.00, 516.00)	463.00 (391.75, 539.75)	0.374
TCO2, mmol/L		23.60 (21.50, 25.98)	22.70 (20.90, 25.55)	23.55 (21.77, 26.20)	24.40 (22.45, 26.10)	<0.001
LDL-C, mmol/L		3.21 (2.44, 4.21)	2.97 (2.31, 4.18)	3.36 (2.49, 4.33)	3.32 (2.55, 4.16)	0.276
TC, mmol/L		4.97 (4.18, 6.14)	4.77 (3.91, 6.29)	5.16 (4.22, 6.15)	4.98 (4.37, 6.00)	0.484
HDL-C, mmol/L		1.16 (0.95, 1.44)	1.14 (0.89, 1.48)	1.17 (0.99, 1.44)	1.17 (0.94, 1.44)	0.661
AST, mmol/L		19.00 (15.00, 24.00)	18.00 (14.50, 23.00)	19.00 (15.00, 23.00)	20.00 (16.00, 25.00)	0.004
ALT, mmol/L		14.75 (11.00, 20.00)	12.00 (9.00, 18.00)	14.55 (11.00, 18.02)	17.00 (13.00, 25.00)	<0.001
Protopathy	Primary Glomerulonephritides	204 (27.95)	75 (27.78)	62 (28.31)	67 (27.80)	0.083
	Hypertensive Renal Disease	19 (2.60)	6 (2.22)	4 (1.83)	9 (3.73)	
	Diabetic nephropathy	40 (5.48)	19 (7.04)	14 (6.39)	7 (2.90)	
	Others	62 (8.49)	20 (7.41)	12 (5.48)	30 (12.45)	
	Unknown	405 (55.48)	150 (55.56)	127 (57.99)	128 (53.11)	
With Hypertension	No	194 (26.58)	58 (21.48)	55 (25.11)	81 (33.61)	0.007
	Yes	536 (73.42)	212 (78.52)	164 (74.89)	160 (66.39)	
With Diabetes mellitus	No	496 (67.95)	166 (61.48)	147 (67.12)	183 (75.93)	0.002
	Yes	234 (32.05)	104 (38.52)	72 (32.88)	58 (24.07)	
With Hyperuricemia	No	661 (90.55)	245 (90.74)	198 (90.41)	218 (90.46)	0.991
	Yes	69 (9.45)	25 (9.26)	21 (9.59)	23 (9.54)	
With Hyperlipidemia	No	619 (84.79)	233 (86.30)	180 (82.19)	206 (85.48)	0.425
	Yes	111 (15.21)	37 (13.70)	39 (17.81)	35 (14.52)	
With Anemia	No	697 (95.48)	241 (89.26)	215 (98.17)	241 (100.00)	<0.001
	Yes	33 (4.52)	29 (10.74)	4 (1.83)	0 (0.00)	
With ACEI/ARB	No	431 (59.04)	160 (59.26)	123 (56.16)	148 (61.41)	0.518
	Yes	299 (40.96)	110 (40.74)	96 (43.84)	93 (38.59)	
With Calcium Supplements	No	564 (77.26)	186 (68.89)	174 (79.45)	204 (84.65)	<0.001
	Yes	166 (22.74)	84 (31.11)	45 (20.55)	37 (15.35)	
With Sodium Bicarbonate	No	466 (63.84)	159 (58.89)	151 (68.95)	156 (64.73)	0.066
	Yes	264 (36.16)	111 (41.11)	68 (31.05)	85 (35.27)	
With Ketoacid Tablets	No	507 (69.45)	172 (63.70)	152 (69.41)	183 (75.93)	0.011
	Yes	223 (30.55)	98 (36.30)	67 (30.59)	58 (24.07)	
With Diuretics	No	559 (76.58)	181 (67.04)	173 (79.00)	205 (85.06)	<0.001
	Yes	171 (23.42)	89 (32.96)	46 (21.00)	36 (14.94)	
With ESAs or Iron	No	587(80.41)	157(58.15)	198(90.41)	232(96.27)	<0.001
	Yes	143(19.59)	113(41.85)	21(9.59)	9(3.7)	
Follow-up duration		35.97 (16.12, 56.18)	27.21 (12.98, 46.97)	38.49 (16.20, 62.08)	42.75 (24.03, 60.69)	<0.001
Composite outcomes (%)	No	473 (64.79)	143 (52.96)	141 (64.38)	189 (78.42)	<0.001
	Yes	257 (35.21)	127 (47.04)	78 (35.62)	52 (21.58)	

Estimated glomerular filtration rate, eGFR; albumin, ALB; uric acid, UA; total carbon dioxide, TCO2; low-density lipoprotein cholesterol, LDL-C; total cholesterol, TC; high-density lipoprotein cholesterol, HDL-C; aspartate transaminase, AST; alanine aminotransferase, ALT; angiotensin converting enzyme inhibitors, ACEI; angiotensin receptor blocker, ARB;Primary Glomerulonephritides included chronic nephritis, nephropathy syndrome and IgA nephropathy.Other secondary nephrosis included systemic lupus erythematosus nephritis, Henoch-Schonlein purpura,Hepatitis B virus-associated nephritis and obstructive nephropathy, etc.; hemoglobin, Hb; hematocrit, HCT.

The median follow-up duration was 35.97 (16.12, 56.18) months with 257 (35.21%) composite outcomes. The features and survival probability comparison of the groups were classified in accordance with the Chinese guideline and consensus (**Supplementary Data Sheet 2**) (14).

### Survival analysis

#### Cox regression

An interaction term with time was introduced for mean_Hb and mean_HCT to construct a time-dependent Cox model. After multiple imputation, pooled results of univariate Cox regression revealed that exposure 1 {baseline_Hb [HR (95% CI): 0.977 (0.972, 0.983) g/L, *p*=0.000]; mean_Hb [HR (95% CI): 0.958 (0.951, 0.965) g/L, *p*<0.000]} and exposure 2 {baseline_HCT [HR (95% CI): 0.918% (0.900%, 0.937%), *p*<0.000]; mean_HCT [HR (95% CI): 0.858% (0.837%, 0.880%), *p*<0.000]} were significantly associated with the composite outcome (**Supplementary Data Sheet 3**).

**Figure 2:**
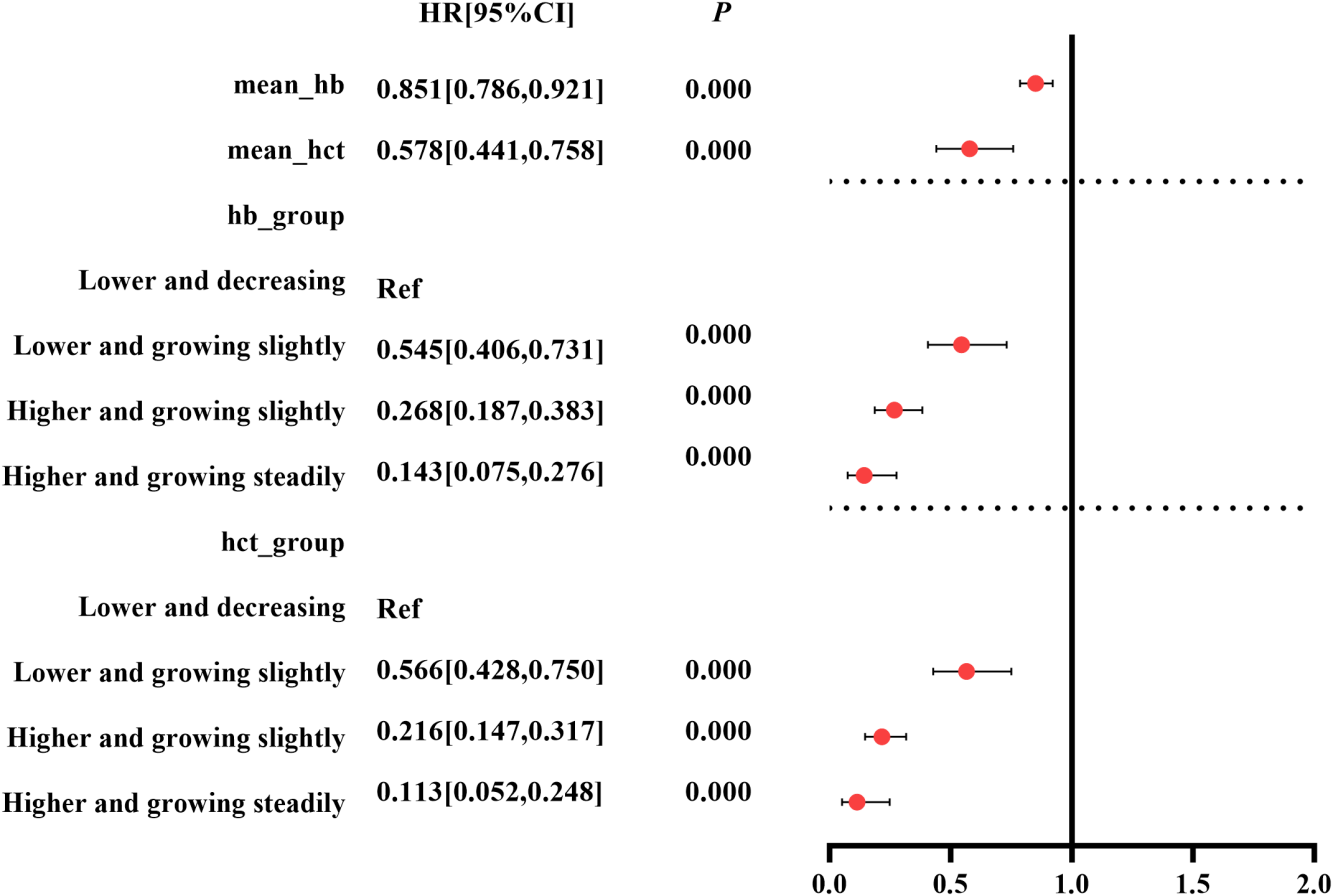
Forest plots of exposure effect estimates.

Taking exposure 1 and exposure 2 as independent variables, respectively, pooled results of multivariate Cox model showed that mean_Hb and mean_HCT were independent factors associated with the composite outcome [mean_Hb: HR (95% CI): 0.851 (0.786, 0.921) g/L, *p*=0.000; mean_HCT: HR (95% CI): 0.578% (0.441%, 0.758%), *p*=0.000] (**Figure 2**, **Supplementary Data Sheet 3**).

### GBTM models

#### Selection of trajectory clusters

**Figure 3:**
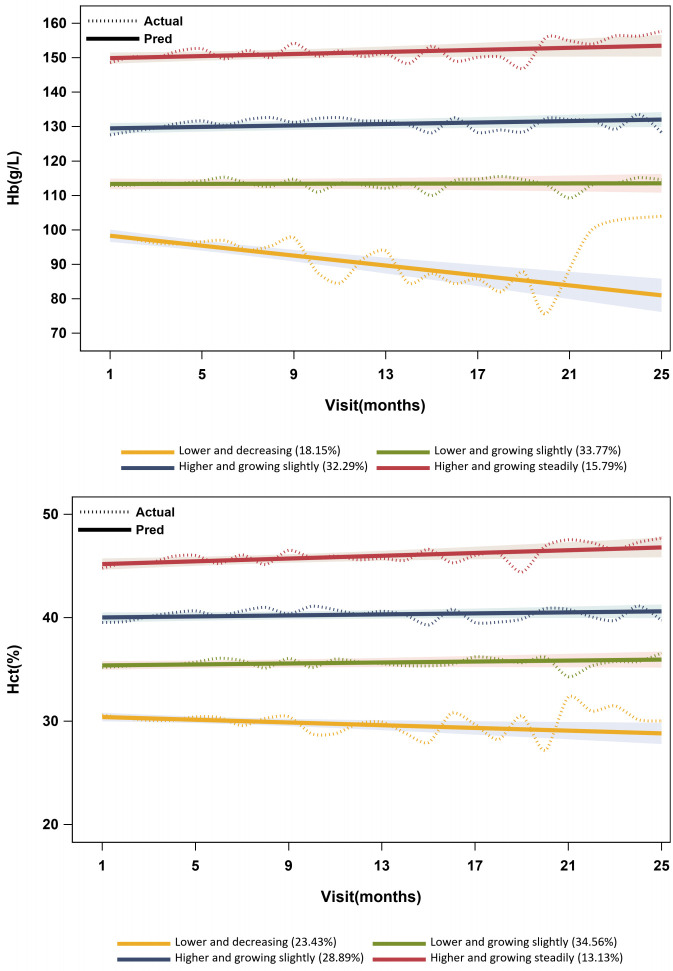
Hb and HCT level trajectories.

The linear form of four groups was selected as the optimal model according to statistical criteria and model fit indices in **Supplementary Data Sheet 4**. The optimal model’s trajectories are visualized in **Figure 3**.

For trajectories in the optimal GBTM model of Hb, group 1 had a lower Hb concentration of less than 100 g/L, with a decreasing trend. Group 2 had a lower Hb concentration between 110 and 120 g/L, with a slight increase [π_j_(%): 33.77%; *P*_j_(%): 34.38%]. The concentration of Hb was higher in group 3 (ranging from 125 to 135 g/L), exhibiting a slight growth. Group 4 displayed a higher concentration of Hb (ranging from 145 to 160 g/L) with a steady increase. For trajectories in the optimal GBTM model of HCT, group 1 had a lower level of HCT (approximately 30%), with a declining trend. Group 2 had a lower level of HCT (approximately 35%), with a slight increase. The level of HCT was higher in group 3 (approximately 40%), exhibiting a slight increase. Group 4 displayed a higher level of HCT (approximately 45%) with a steady increase. In addition, group 1 displayed considerable fluctuations, while groups 2 to 4 exhibited relatively smooth curves in both GBTM models of Hb and HCT (**Figure 3**).

Comparison of baseline characteristics indicated that exposure 1, exposure 2, age, sex, eGFR, ALB, urea, TCO_2_, AST, ALT, etiology, with hypertension, with diabetes mellitus, with anemia, use of calcium supplements, and use of diuretics were statistically significant between trajectories in both GBTM models of Hb and HCT (*p*<0.05) (**Supplementary Data Sheet 4**).

#### Cox regression

**Figure 4:**
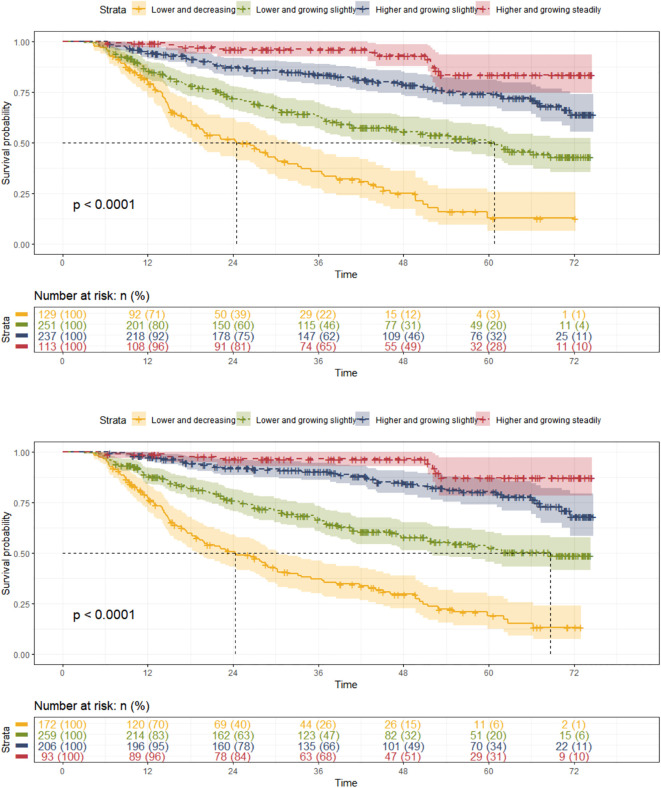
Kaplan-Meier survival analysis of Hb and HCT trajectories.

Both Hb and HCT trajectory groups showed a significant difference in survival probability (*p*<0.0001) (**Figure 4**). The median survival time for Hb trajectory group 1 was 24.5 months with 83 (64.34%) composite outcomes, and that for Hb trajectory group 2 was 60.8 months with 108 (43.03%) composite outcomes. HCT trajectory group 1 has a median survival time of 24.3 months with 113 (65.70%) composite outcomes, and HCT trajectory group 2 has a median survival time of 68.7 months with 100 (38.61%) composite outcomes.

Taking group 1 as the control group, the univariate Cox regression showed that Hb and HCT trajectory groups 2 to 4 had potential protective effects against the composite outcome in patients with CKD stages 3–4 [Hb: group 2 HR (95% CI): 0.429 (0.321, 0.573), *p* = 0.000; group 3 HR (95% CI): 0.184 (0.130, 0.261), *p*<0.000; group 4 HR (95% CI): 0.075 (0.040, 0.142), *p* = 0. 000; HCT: group 2 HR (95% CI): 0.390 (0.297, 0.512), *p* = 0.000; group 3 HR (95% CI): 0.143 (0.098, 0.208), *p*<0.000; group 4 HR (95% CI): 0.059 (0.027, 0.126), *p* = 0.000] (**Supplementary File 4**). After adjusting for covariates, the pooled results of the multivariate Cox regression showed that Hb and HCT trajectory groups 2 to 4 were independently protective factors thwarting the occurrence of composite outcome in patients with CKD stages 3–4 with a significant p for trend [Hb: group 2 HR (95% CI): 0.545 (0.406, 0.731), *p* = 0.000; group 3 HR (95% CI):0.268 (0.187, 0.383), *p*<0.000; group 4 HR (95% CI): 0.143 (0.075, 0.276), *p* =0.000; HCT: group 2 HR (95% CI): 0.566 (0.428, 0.75), *p* = 0.000; group 3 HR (95% CI): 0.216 (0.147, 0.317), *p*<0.000; group 4 HR (95% CI): 0.113 (0.052, 0.248), *p* = 0.000] (**Figure 2**, **Supplementary File 4**).

#### Subgroup analyses

In order to investigate the potential interactions between subgroups of age, sex, CKD stage, with hypertension, and with diabetes mellitus and the composite outcome, subgroup analyses were conducted based on the final multivariate Cox model for each dataset.

The results showed that mean_Hb and mean_HCT were protective factors for subgroups of age <59.3 years, age ≥59.3 years, female, male, CKD stage 3, with hypertension, with diabetes mellitus, and without diabetes mellitus (*p*<0.05) and were not statistically significant in the subgroups of CKD stage 4 and without hypertension. There was an interaction effect between mean_Hb and subgroup of sex (*p* for interaction=0.034) (**Supplementary Data Sheet 5**).

Taking group 1 as the control group, groups 2 to 4 were protective factors for subgroups of age <59.3 years, age ≥59.3 years, female, male, CKD stage 3, with hypertension, with diabetes mellitus, and without diabetes mellitus (*p*<0.05). There was an interaction effect between Hb trajectory group 2 and CKD stage (*p* for interaction=0.015) (**Supplementary Data Sheet 5**).

### Sensitivity analyses

We repeated the above analyses with complete baseline data, and the results showed that the mean_Hb [HR (95% CI): 0.862 (0.782, 0.950), *p*=0.003], mean_HCT [HR (95% CI): 0.580 (0.416, 0.808), *p*=0.002], and Hb and HCT trajectory groups 2 to 4 [using group 1 as reference, Hb: group 2 HR (95% CI): 0.573 (0.394, 0.831), *p*=0.003; group 3 HR (95% CI): 0.230 (0.144, 0.368), *p*=0.000; group 4 HR (95% CI): 0.166 (0.083, 0.334), *p*=0.000; HCT: group 2 HR (95% CI): 0.460 (0.321, 0.660), *p*=0.000; group 3 HR (95% CI): 0.173 (0.105, 0.285), *p*=0.000; group 4 HR (95% CI): 0.121 (0.055, 0.266), *p*=0.000] were also independent protective factors for the occurrence of composite outcomes in patients with CKD stages 3–4. Both the Hb and HCT trajectories had a significant increasing trend in HRs (*p* for trend=0.000) (**Supplementary Data Sheet 6**).

## Discussion

We retrospectively collected demographic and clinical information over a 6-year follow-up period to investigate the association between Hb and HCT and the prognosis of patients with CKD stages 3–4 in Lingnan, China. The final cohort comprised 730 patients with CKD stages 3–4 in accordance with the inclusion and exclusion criteria. The baseline Hb level that met the recommended range of the Chinese renal anemia guideline was approximately 60%–63%, which was comparable to the Hb attainment rate of Japanese patients with CKD stages 3–4 and slightly higher than that of patients with CKD stages 3–4 in the United States (20, 21). Moreover, the proportion of individuals in the substandard group exhibited a decline from 40.0% to 14.8% over the course of the follow-up period, which may be related to implementation of effective anemia management strategies and a higher incidence of the composite outcome among the substandard group.

We constructed a time-dependent Cox model with baseline and first-year mean values of Hb and HCT as independent variables, and a GBTM model with longitudinal measurements of Hb and HCT, respectively, to explore the association of Hb and HCT with the short- and long-term risk of composite outcomes of patients with CKD stages 3–4. The results of the time-dependent Cox model showed that after adjusted for covariates, the mean_Hb [HR (95% CI): 0.851 (0.786, 0.921) g/L, *p*=0.000] and the mean_HCT [HR (95% CI): 0. 578 (0.441, 0.758) %, *p*=0.000] were the independent protective factors and baseline_Hb and baseline_HCT were not independent factors associated with the composite outcome. It reveals a closed relationship between mean_Hb and mean_HCT and CKD prognosis, and on the other hand, it suggests that levels of Hb and HCT may vary with the progression of the disease and the different treatments employed in patients with CKD stages 3–4. While baseline values remain clinically important for initial assessment and treatment initiation, our findings suggest that longitudinal monitoring of Hb and HCT levels provides superior prognostic information compared to single-point measurements. The findings indicate that patients with CKD require effective management and maintenance of their levels of Hb and HCT over time to improve their prognosis.

To gain further insight into the management of long-term anemia in patients with CKD, we constructed a GBTM using longitudinal data. This approach enabled us to divide the persistence values of Hb and HCT in these patients into four distinct groups based on their trajectory. We then compared the prognosis of these groups. Taking group 1 (“lower and decreasing”; Hb<100 g/L, HCT approximately 30%) as reference, group 2 (“lower and growing slightly”; Hb 110–120 g/L, HCT approximately 35%), group 3 (“higher and growing slightly”; Hb 125–135 g/L, HCT approximately 40%), and group 4 (“higher and growing steadily”; Hb 145–160 g/L, HCT approximately 45%) were independent protective factors for the occurrence of the composite outcome in patients with CKD stages 3–4 (*p*=0. 000). Group 1 (“lower and decreasing”; Hb<100 g/L, HCT approximately 30%) had more fluctuations than groups 2 to 4, which were relatively stable. As Hb and HCT moved from a lower to a higher level, the HRs decreased gradually, indicating a gradual decreased risk of composite outcomes in patients with CKD stages 3–4 (*p* for trend<0.000). We further compared baseline characteristics between groups 4 and 1. Compared with group 1, group 4 exhibited fewer comorbidities, a lower proportion of certain medications, and a more favorable physiologic and metabolic profile (**Supplementary Data Sheet 4**). These findings suggest that younger patients with fewer comorbidities may better tolerate higher Hb levels, and that preserved kidney function and better nutritional status may attenuate the risks associated with higher Hb.

The results of both the time-dependent Cox model and the GBTM model reveals that higher levels of Hb and HCT had a lower risk of short- and long-term disease progression in CKD. This finding is broadly consistent with previous studies in Asian populations (15). Previous studies have postulated that the observed improvement in hematopoiesis may be attributed to a reduction in metabolic pressure in the proximal tubules or adjacent interstitium of the kidney during the treatment process. This reduction in pressure is hypothesized to enhance the production of red blood cells, as evidenced by elevated levels of Hb and HCT. Therefore, higher levels of Hb and HCT can be considered a surrogate indicator of improved renal function (22). Subgroup analyses showed an interaction effect of mean_Hb with the subgroup of sex (*p* for interaction=0.034) and an interaction effect of group 2 of Hb trajectory with the subgroup of CKD stage (*p* for interaction=0.015). It suggests that the effect of Hb and HCT on the composite outcome may vary by sex and CKD stages, especially those with Hb levels ranging from 110 to 120 g/L.

We collected clinical data of patients with non-dialysis CKD based on a retrospective cohort study, attributing to a limited accuracy of determinations of cardiovascular outcomes. In view of this, we did not further investigate the association between Hb and HCT and cardiovascular outcomes in patients with CKD. However, it is worth noting that previous studies have shown that too high a level of Hb may be associated with an increased risk of cardiovascular events. For example, The Correction of Hemoglobin and Outcomes in Renal Insufficiency (CHOIR) trial included 1,432 patients with CKD stages 3–4 with anemia (defined as Hb <110 g/L) and randomly arranged them to groups with a higher or lower treatment target (135 g/L vs. 113 g/L) with a study duration of 3 years. It showed that the group with a higher treatment target had a higher HR (95% CI) of 1.34 (1.03,1.74) (*p*=0.03) after the occurrence of the first cardiovascular events compared to the group with a lower treatment target and no significant gain in quality of life (16). Some researchers had doubts about the conclusion because the group with the higher treatment target had a higher proportion of history of hypertension and history of undergoing coronary artery bypass grafting and a greater severity of congestive heart failure than the group with the lower treatment target at baseline (23). After adjusting for heart failure scores at baseline, the correlation between the two groups and the primary composite outcome was not significantly different [HR (95% CI) 1.24 (0.95, 1.62), *p*=0.11] (24). The Cardiovascular Risk Reduction by Early Anemia Treatment with Epoetin beta (CREATE) study obtained an opposite conclusion. It included 603 CKD stages 3b–4 patients with mild to moderate anemia (defined as Hb between 110 and 125 g/L) and also randomized them into groups with a higher or a lower treatment target (130–150 g/L vs. 105–115 g/L) with a study duration of 3 years. It showed that the group with a higher treatment target had a higher HR (95% CI) of 0. 78 (0.53, 1.14) (*p*=0.20) after the occurrence of the first cardiovascular events and had a higher quality of life than the group with the lower treatment target (17). The Kidney Disease Outcomes Quality Initiative (KDOQI) work group pooled the results of six related studies, and it showed that groups with higher Hb targets yielded a relative risk (RR) (95% CI) of 1.24 (1.02, 1.51) on adverse cardiovascular events in patients with non-dialysis CKD. However, the CHOIR trial and the CREATE study contributed 94% of the weight in this meta-analysis (25).

In light of the previous studies and the findings of our study, it is evident that there is a discernible advantage to maintaining a higher level of Hb and HCT over time in patients with CKD stages 3–4 to thwart the progress of CKD [including all-cause mortality, ESRD (RRT and eGFR <15 mL/min/1. 73m^2^), doubling of SCr from baseline, and ≥50% reduction in eGFR from baseline]. It should be noted that this benefit is contingent upon maintaining Hb and HCT at a relatively stable level over time, rather than merely reaching a specific point in time. In our study, we also did not find that having excessive levels of Hb and HCT were detrimental to the composite outcome. We think that it is important to note that the level of Hb and cardiovascular events remains controversial. Our retrospective study had limitations in exploring this issue. From a more cautious perspective, we recommend that Hb levels be maintained within the range of 110 to 130 g/L in patients with non-dialysis CKD.

## Limitations

First, although our study collected as much information as possible and used Cox regression and GBTM methods to adjust for confounding factors, it is important to note that the retrospective study design itself still has limitations in terms of study design and data collection. There are still potential confounding factors that cannot be fully adjusted. It should be noted that, because this is a retrospective study and we lacked data on cumulative ESA or iron doses and dosing frequency, residual confounding related to treatment intensity and medication response may persist. Additionally, as in many retrospective studies, selection bias is possible—particularly if less healthy patients are more likely to be lost to follow-up. Consistent with this concern, our baseline comparisons indicated potential selection bias: the included cohort was younger; had lower eGFR, Hb, and HCT; had higher urea and slightly higher ALT; had more frequent use of sodium bicarbonate and potassium-lowering agents but less calcium supplementation; and had a longer follow-up with fewer composite outcomes than the excluded cohort (**Supplementary Data Sheet 1**). Second, Hb and HCT are subject to short−term biological and pre−analytical variability (e.g., diurnal rhythms, hydration, and volume status), which may introduce minor bias. To mitigate this, baseline laboratories were obtained primarily under standardized procedures at GPHCM, and we adjusted for covariates related to volume and nutrition (e.g., eGFR, serum albumin, use of diuretics and ACEI/ARB, and presence of hypertension). Residual short-term variability may remain, but these steps reduce its impact. In addition, despite aggregating measurements into 3-month windows for GBTM, some trajectory misclassification related to irregular measurement timing may persist. Third, although the association between Hb level and risk of cardiovascular events remains unclear, there is a theoretical possibility of competing risks of cardiovascular outcomes in our study, and analysis of this potential risk was not possible given the limitations of the data. Fourth, the size of our study sample is relatively limited, and further increases in sample size, improvements in study methodology, or additional investigations of associated factors are required to enhance our understanding of the pathogenesis of the disease and predict future outcomes for patients. Fifth, it should be noted that our study only includes data from southern China, and its generalizability needs to be considered.

## Data Availability

The raw data supporting the conclusions of this article will be made available by the authors, without undue reservation.
