# Supplementary material for: Time-updated patterns of hemoglobin and hematocrit and the risk of CKD progression
Source: Front Endocrinol (Lausanne). 2025 Oct 30;16:1642307. doi: 10.3389/fendo.2025.1642307 (PMC12611651; doi:10.3389/fendo.2025.1642307)

**Supplementary file1**

Supplementary file1.1 Baseline Characteristics of Included and Excluded Groups

| Variables |  | Included group  (N=730) | Excluded group  (N=2785) | *P* |
| --- | --- | --- | --- | --- |
|  |  | Mean (SD)/Median (IQR)/N(%) | |  |
| Age, year |  | 59.00(48.00,68.00) | 63.00(51.00,71.00) | <0.001 |
| Sex | Female | 306 (41.92) | 1150 (41.29) | 0.793 |
|  | Male | 424 (58.08) | 1635 (58.71) |  |
| eGFR, ml/min/1.73 m2 |  | 39.41(26.39,50.92) | 42.64(30.48,51.89) | <0.001 |
| ALB, g/L |  | 40.9(35.90,44.50) | 41.10(36.90,44.20) | 0.522 |
| Hb, g/L |  | 121.00(107.00,135.00) | 124.00(110.0,138.00) | 0.002 |
| HCT, % |  | 36.80(32.63,40.80) | 37.70(33.60,41.60) | 0.001 |
| Urea, mmol/L |  | 9.20(7.30,11.92) | 8.51(6.80,11.12) | <0.001 |
| UA, mmol/L |  | 454.85(383.00,529.00) | 455.00(384.00,538.00) | 0.815 |
| TCO2, mmol/L |  | 23.60(21.50,25.98) | 23.95(21.90,26.00) | 0.140 |
| TC, mmol/L |  | 4.97(4.18,6.14) | 4.96(4.12,5.88) | 0.109 |
| AST, mmol/L |  | 19.00(15.00,24.00) | 19.00(16.00,24.00) | 0.055 |
| ALT, mmol/L |  | 14.75(11.0,20.00) | 15.00(11.0,22.00) | 0.012 |
| With Hypertension | No | 194 (26.58) | 760 (27.29) | 0.734 |
|  | Yes | 536 (73.42) | 2025 (72.71) |  |
| With Diabetes mellitus | No | 496 (67.95) | 1802 (64.70) | 0.111 |
|  | Yes | 234 (32.05) | 983 (35.30) |  |
| With Hyperuricemia | No | 661 (90.55) | 2547 (91.45) | 0.485 |
|  | Yes | 69 (9.45) | 238 (8.55) |  |
| With Hyperlipidemia | No | 619 (84.79) | 2316 (83.16) | 0.316 |
|  | Yes | 111 (15.21) | 469 (16.84) |  |
| With Anemia | No | 697 (95.48) | 2667 (95.76) | 0.815 |
|  | Yes | 33 (4.52) | 118 (4.24) |  |
| With ACEI/ARB | No | 431 (59.04) | 1700 (61.04) | 0.346 |
|  | Yes | 299 (40.96) | 1085 (38.96) |  |
| With Calcium Supplements | No | 564 (77.26) | 2339 (83.99) | <0.001 |
|  | Yes | 166 (22.74) | 446 (16.01) |  |
| With Sodium Bicarbonate | No | 466 (63.84) | 2050 (73.61) | <0.001 |
|  | Yes | 264 (36.16) | 735 (26.39) |  |
| With Ketoacid Tablets | No | 507 (69.45) | 2209 (79.32) | <0.001 |
|  | Yes | 223 (30.55) | 576 (20.68) |  |
| With Diuretics | No | 559 (76.58) | 2186 (78.49) | 0.287 |
|  | Yes | 171 (23.42) | 599 (21.51) |  |
| With ESAs or Iron | No | 587(80.41) | 2454(88.11) | <0.001 |
|  | Yes | 143(19.59) | 331(11.89) |  |
| Follow-up duration |  | 37.64(16.88,63.59) | 31.77(12.85,61.08) | <0.001 |
| Composite outcomes (%) | No | 445 (60.96) | 2078 (74.61) | <0.001 |
|  | Yes | 285 (39.04) | 707 (25.39) |  |

Note: Excluded group: participants with missing repeated Hb/HCT measurements required for trajectory analysis. estimated glomerular filtration rate, eGFR; albumin, ALB; uric acid, UA; total carbon dioxide, TCO2; ; total cholesterol, TC; ; aspartate transaminase, AST; alanine aminotransferase, ALT; angiotensin converting enzyme inhibitors, ACEI; angiotensin receptor blocker, ARB; hemoglobin, Hb; hematocrit, HCT.

Compared with excluded participants, the included group was younger (59.00 (48.00, 68.00) vs 63.00 (51.00, 71.00) years, *P*<0.001) and had lower eGFR (39.41 (36.36, 50.92) vs 42.64 (30.85, 51.39) ml/min/1.73 m², *P*<0.001), with lower Hb and HCT (Hb: 121.00 (107.00, 135.00) vs 124.00 (109.00, 138.00) g/L, *P*=0.002; HCT: 36.30 (32.63, 40.80) vs 37.70 (33.63, 41.60)%, *P*<0.001). Urea was higher (9.07 (7.30, 11.92) vs 8.52 (6.80, 11.12) mmol/L, *P*<0.001), and ALT was slightly higher (17.45 (11.00, 22.00) vs 15.01 (11.00, 22.00) U/L, *P*=0.012) in the included group. The excluded group had larger proportion of calcium supplementation (89.39% vs 77.26%, *P*<0.001) and ESAs or Iron(19.59% vs 11.89%, *P*<0.001), whereas sodium bicarbonate and potassium‑lowering agents were more common in the included group (both *P*≤0.001). Follow‑up duration was longer in the included group (37.64 (18.68, 53.59) vs 31.71 (26.85, 61.08) months, *P*<0.001), while composite outcomes were more frequent in the excluded group (74.6% vs 61.0%, *P*<0.001). Systematic differences in age, kidney function, hematologic and metabolic profiles, medication use, follow‑up length, and outcome rates indicate potential selection bias.

Supplementary file1.2 Missing rate of covariates

| Covariates | Missing rate, % | Covariates | Missing rate, % | Covariates | Missing rate, % | Covariates | Missing rate, % |
| --- | --- | --- | --- | --- | --- | --- | --- |
| Alb | 16.58 | **P** | **44.38** | LDL-C | 26.30 | **UPCR** | **59.45** |
| Urea | 3.01 | **Ca^2+^** | **42.19** | TC | 20.41 | AST | 16.99 |
| UA | 8.49 | **K^+^** | **38.77** | HDL-C | 27.81 | ALT | 16.97 |
| TCO2 | 10.41 | **Na^+^** | **39.45** | **Glu** | **46.16** |  |  |

Supplementary file1.3 Collinearity of variables


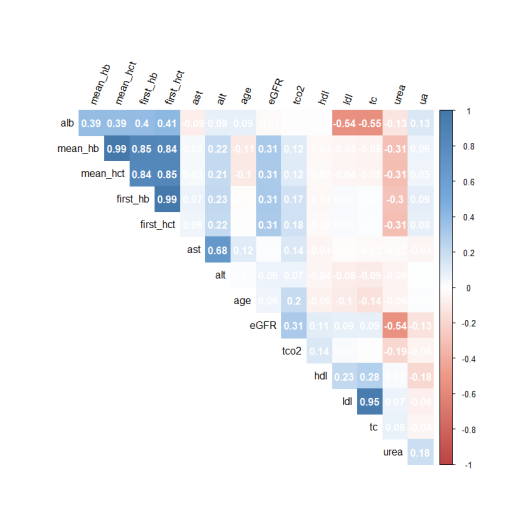


Supplementary file 1.4 Kaplan-Meier Curve of whole cohort


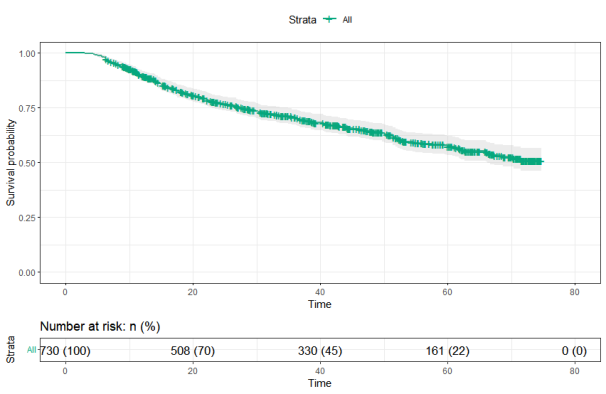

Supplement: Supplementary file 1 [file DataSheet1.docx]
