# Supplementary material for: Time-updated patterns of hemoglobin and hematocrit and the risk of CKD progression
Source: Front Endocrinol (Lausanne). 2025 Oct 30;16:1642307. doi: 10.3389/fendo.2025.1642307 (PMC12611651; doi:10.3389/fendo.2025.1642307)
Supplement: Supplementary file 2 [file DataSheet2.docx]

**Supplementary file 2**

Baseline_Hb and its level during follow-up duration was classified into three groups: substandard group (<115g/L), standard group (≥115g/L, ≤130g/L) and excess group (>130g/L) according to the expert consensus on renal anaemia in China. ^[14]^

270 individuals were in the substandard group, 219 individuals were in the standard group and 241 individuals were in the excess group. Sex, eGFR, ALB, urea, TCO2, AST, ALT, with hypertension, with diabetes mellitus, with anaemia, use of calcium supplements, use of ketoacid tablets and use of diuretics were statistically significant between the three groups. The median follow-up duration of the substandard group, standard group, and excess group was 27.21 (12.98, 46.97), 38.49 (16.20, 62.08), and 42.75 (24.03, 60.69) months, with composite outcomes occurring in 127 (47.04%), 78 (35.62%), and 52 (21.58%) patients, respectively. The Kaplan-Merier curve of groups were displayed in Figure 1.


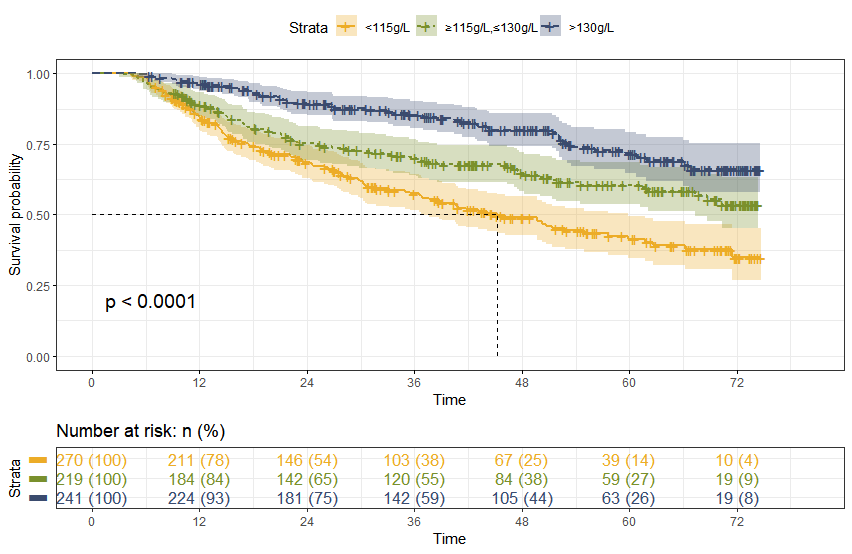


Figure1 Kaplan-Meier Curve of Hb <115g/L, 115g/L≤,≤130g/L and >130g/L

For baseline_Hb, 37.0% individuals were in the substandard group, 30.0% in the standard group and 33.0% in the excess group at baseline. After 6-year follow-up duration, the proportion of substandard group decreased to 14.8%, the proportion of standard group increased to 38.3%, and the proportion of excess group increased to 46.9%. For mean_Hb, 40.0% individuals were in the substandard group, 31.6% in the standard group and 28.4% in the excess group at baseline. After 6-year follow-up duration, the proportion of substandard group decreased to 16.4%, the proportion of standard group increased to 35.2%, and the proportion of excess group increased to 48.4%. The distribution of baseline_Hb and mean_Hb was relatively balanced, with a gradual shift towards imbalance over the follow-up period (Figure 2).


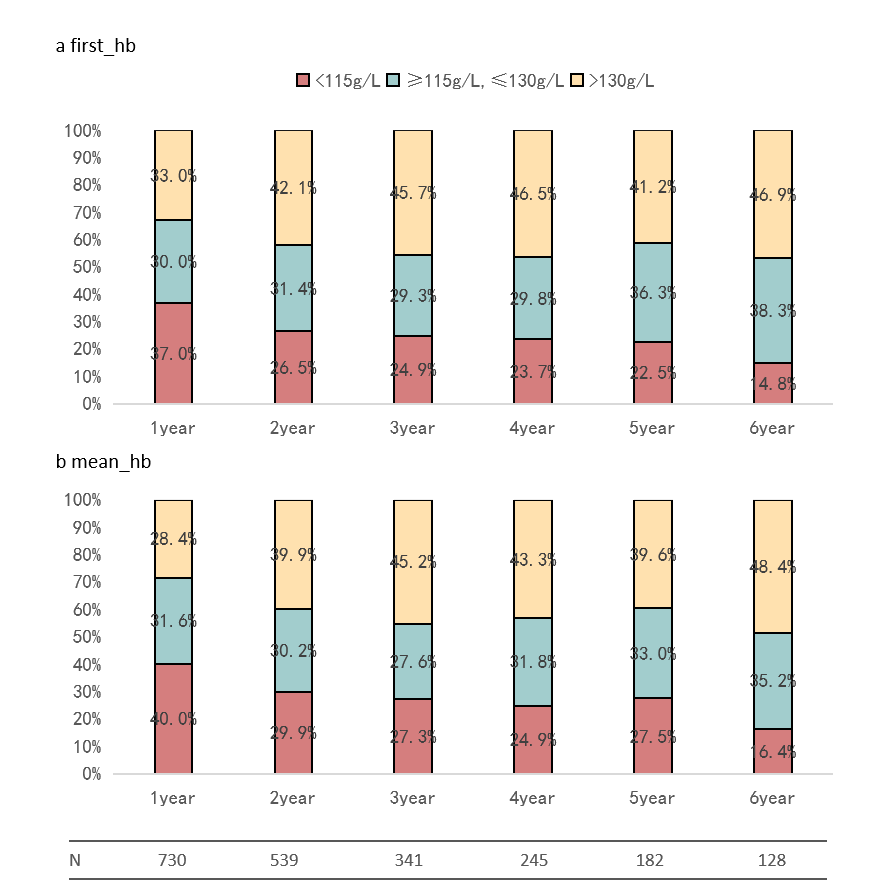


Figure 2 Follow-up characteristics of Hb
