# Supplementary material for: Time-updated patterns of hemoglobin and hematocrit and the risk of CKD progression
Source: Front Endocrinol (Lausanne). 2025 Oct 30;16:1642307. doi: 10.3389/fendo.2025.1642307 (PMC12611651; doi:10.3389/fendo.2025.1642307)
Supplement: Supplementary file 3 [file DataSheet3.docx]

**Supplementary file 3**

Supplementary file 3.1 Test for assumption and Univariate Cox regression

Table 1 Test for assumption, Univariate Cox regression of demographic information, clinical diagnosis and medication data

| Variables |  | HR(95%CI) | *P* for univariate Cox regression | *P* for Schoenfeld Individual Test |
| --- | --- | --- | --- | --- |
| Age, year |  | 0.989(0.981,0.996) | 0.011 | 0.806 |
| eGFR, ml/min/1.73 m^2^ |  | 0.941(0.932,0.951) | <0.000 | 0.059 |
| Sex | Female | Ref |  |  |
|  | Male | 0.871(0.682,1.114) | 0.272 | 0.771 |
| With Hypertension | No | Ref |  |  |
|  | Yes | 1.861(1.356,2.554) | 0.000 | 0.435 |
| With Diabetes | No | Ref |  |  |
|  | Yes | 1.815(1.416,2.326) | 0.000 | 0.100 |
| With Hyperuricemia | No | Ref |  |  |
|  | Yes | 1.120(0.735,1.705) | 0.599 | 0.220 |
| With Hyperlipidemia | No | Ref |  |  |
|  | Yes | 1.166(0.840,1.617) | 0.359 | 0.707 |
| With Anemia | No | Ref |  |  |
|  | Yes | 1.465(0.838,2.561) | 0.180 | 0.088 |
| Protopathy | Primary Glomerulonephritides | Ref |  | 0.494 |
|  | Hypertensive Renal Disease | 1.002(0.459,2.185) | 0.996 |  |
|  | Diabetic nephropathy | 2.600(1.649,4.100) | 0.000 |  |
|  | Others | 0.556(0.306,1.009) | 0.053 |  |
|  | Unknown | 1.136(0.848,1.522) | 0.392 |  |
| With ACEI/ARB | No | Ref |  |  |
|  | Yes | 0.995(0.777,1.275) | 0.970 | 0.907 |
| With Calcium Supplements | No | Ref |  |  |
|  | Yes | 0.786(0.573,1.078) | 0.135 | 0.189 |
| With Sodium Bicarbonate | No | Ref |  |  |
|  | Yes | 1.418(1.108,1.815) | 0.006 | 0.697 |
| With Ketoacid Tablets | No | Ref |  |  |
|  | Yes | 1.459(1.134,1.878) | 0.003 | 0.170 |
| With Diuretics | No | Ref |  |  |
|  | Yes | 1.396(1.058,1.842) | 0.019 | 0.050 |
| With ESAs or Iron | No | Ref |  |  |
|  | Yes | 1.970(1.496,2.595) | 0.000 | 0.273 |

Note: angiotensin converting enzyme inhibitors, ACEI; Angiotensin receptor blocker, ARB;Primary Glomerulonephritides included chronic nephritis, nephropathy syndrome and IgA nephropathy.Other secondary nephrosis included systemic lupus erythematosus nephritis, Henoch-Schonlein purpura,Hepatitis B virus-associated nephritis and obstructive nephropathy, etc.

Table 2 Test for assumption, Univariate Cox regression of laboratory parameters

| Variables | HR(95%CI) | *P* for univariate Cox regression | *P* for Schoenfeld Individual Test |
| --- | --- | --- | --- |
| Dataset1 |  |  |  |
| ALB, g/L | 0.978(0.963,0.994) | 0.006 | 0.046 |
| Urea, mmol/L | 1.088(1.071,1.105) | <0.000 | 0.050 |
| UA, mmol/L | 1.001(1.000,1.002) | 0.237 | 0.594 |
| TCO2, mmol/L | 0.928(0.897,0.961) | 0.000 | 0.249 |
| LDL-C, mmol/L | 1.001(0.933,1.073) | 0.984 | 0.733 |
| TC, mmol/L | 1.018(0.957,1.081) | 0.576 | 0.782 |
| HDL-C, mmol/L | 0.890(0.648,1.222) | 0.470 | 0.137 |
| AST, mmol/L | 1.002(0.993,1.012) | 0.654 | 0.046 |
| ALT, mmol/L | 0.998(0.991,1.006) | 0.660 | 0.292 |
| Dataset2 |  |  |  |
| ALB, g/L | 0.973(0.959,0.988) | 0.001 | 0.113 |
| Urea, mmol/L | 1.086(1.069,1.104) | <0.000 | 0.059 |
| UA, mmol/L | 1.001(1.000,1.002) | 0.071 | 0.941 |
| TCO2, mmol/L | 0.927(0.893,0.961) | 0.000 | 0.182 |
| LDL-C, mmol/L | 1.026(0.959,1.097) | 0.458 | 0.764 |
| TC, mmol/L | 1.023(0.963,1.087) | 0.460 | 0.796 |
| HDL-C, mmol/L | 0.818(0.590,1.134) | 0.227 | 0.222 |
| AST, mmol/L | 1.134(0.996,1.013) | 0.302 | 0.048 |
| ALT, mmol/L | 1.000(0.993,1.007) | 0.926 | 0.213 |
| Dataset3 |  |  |  |
| ALB, g/L | 0.975(0.960,0.990) | 0.001 | 0.124 |
| Urea, mmol/L | 1.088(1.071,1.105) | <0.000 | 0.052 |
| UA, mmol/L | 1.001(1.000,1.002) | 0.239 | 0.660 |
| TCO2, mmol/L | 0.927(0.893,0.962) | 0.000 | 0.040 |
| LDL-C, mmol/L | 1.011(0.945,1.082) | 0.748 | 0.922 |
| TC, mmol/L | 1.019(0.959,1.082) | 0.552 | 0.944 |
| HDL-C, mmol/L | 0.912(0.668,1.244) | 0.561 | 0.563 |
| AST, mmol/L | 1.004(0.995,1.013) | 0.380 | 0.030 |
| ALT, mmol/L | 1.000(0.993,1.007) | 0.995 | 0.180 |
| Dataset4 |  |  |  |
| ALB, g/L | 0.978(0.962,0.993) | 0.005 | 0.050 |
| Urea, mmol/L | 1.088(1.071,1.105) | <0.000 | 0.044 |
| UA, mmol/L | 1.001(1.000,1.002) | 0.214 | 0.797 |
| TCO2, mmol/L | 0.919(0.886,0.953) | 0.000 | 0.102 |
| LDL-C, mmol/L | 1.031(0.966,1.100) | 0.360 | 0.481 |
| TC, mmol/L | 1.040(0.982,1.102) | 0.181 | 0.501 |
| HDL-C, mmol/L | 0.915(0.671,1.247) | 0.572 | 0.374 |
| AST, mmol/L | 1.003(0.994,1.013) | 0.483 | 0.019 |
| ALT, mmol/L | 0.999(0.992,1.007) | 0.884 | 0.187 |
| Dataset5 |  |  |  |
| ALB, g/L | 0.976(0.960,0.991) | 0.002 | 0.022 |
| Urea, mmol/L | 1.087(1.070,1.105) | <0.000 | 0.046 |
| UA, mmol/L | 1.001(1.000,1.002) | 0.230 | 0.500 |
| TCO2, mmol/L | 0.924(0.890,0.959) | 0.000 | 0.189 |
| LDL-C, mmol/L | 1.015(0.949,1.086) | 0.661 | 0.842 |
| TC, mmol/L | 1.011(0.950,1.075) | 0.736 | 0.721 |
| HDL-C, mmol/L | 0.903(0.661,1.232) | 0.519 | 0.360 |
| AST, mmol/L | 1.006(0.998,1.014) | 0.170 | 0.152 |
| ALT, mmol/L | 1.000(0.993,1.007) | 0.916 | 0.396 |

Note:estimated glomerular filtration rate, eGFR; albumin, ALB; uric acid, UA; total carbon dioxide, TCO2; low-density lipoprotein cholesterol, LDL-C; total cholesterol, TC; high-density lipoprotein cholesterol, HDL-C; aspartate transaminase, AST; alanine aminotransferase, ALT.

Table 3 Test for assumption, Univariate Cox regression of exposures

| Variables | HR(95%CI) | *P* for univariate Cox regression | *P* for Schoenfeld Individual Test | *P* for Non-linear |
| --- | --- | --- | --- | --- |
| Exposure 1 |  |  |  |  |
| baseline_Hb, g/L | 0.977(0.972,0.983) | 0.000 | 0.316 | 0.158 |
| mean_Hb, g/L | 0.958(0.951,0.965) | <0.000 | 0.001 | 0.618 |
| Exposure 2 |  |  |  |  |
| baseline_HCT, % | 0.918(0.900,0.937) | <0.000 | 0.380 | 0.046* |
| mean_HCT, % | 0.858(0.837,0.880) | <0.000 | 0.001 | 0.743 |

Note: hemoglobin, Hb; hematocrit, HCT.

Supplementary file 3.2 Lasso selection

| Dataset 1 |  |
| --- | --- |
| 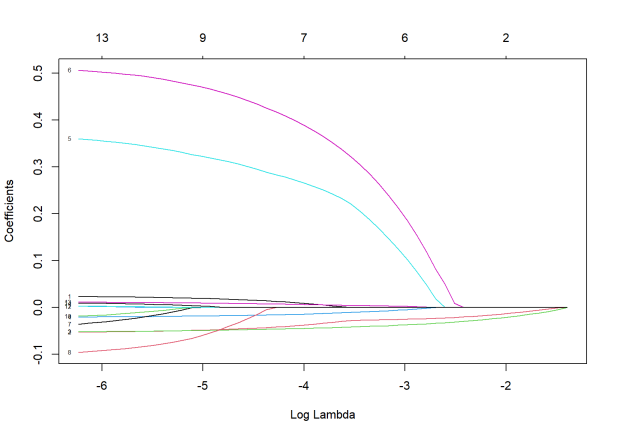 | 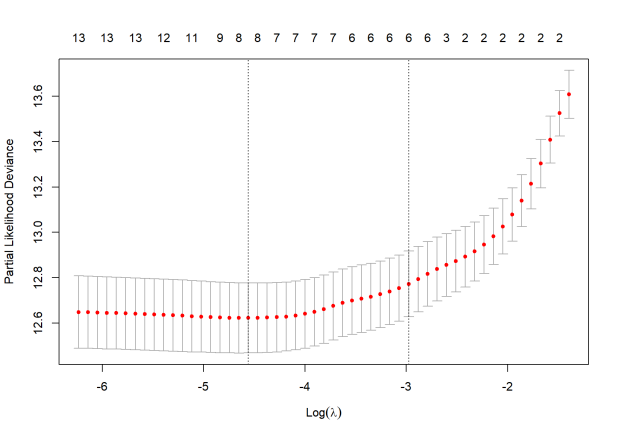 |
| Dataset 2 |  |
| 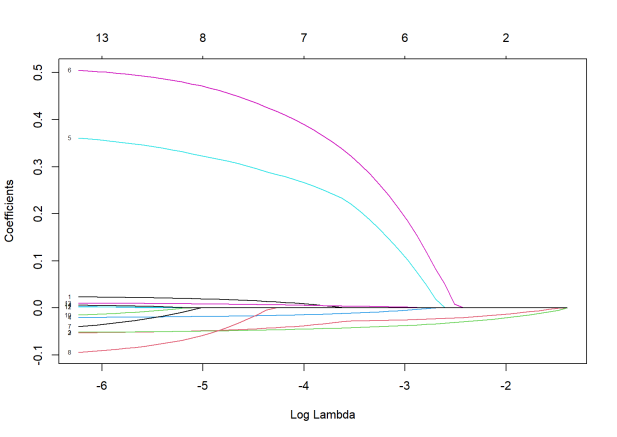 | 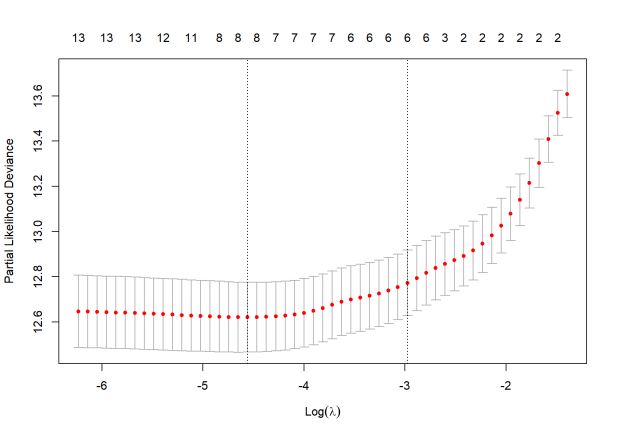 |
| Dataset 3 |  |
| 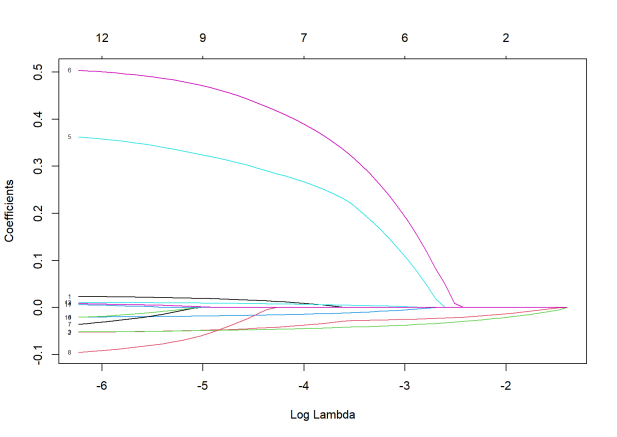 | 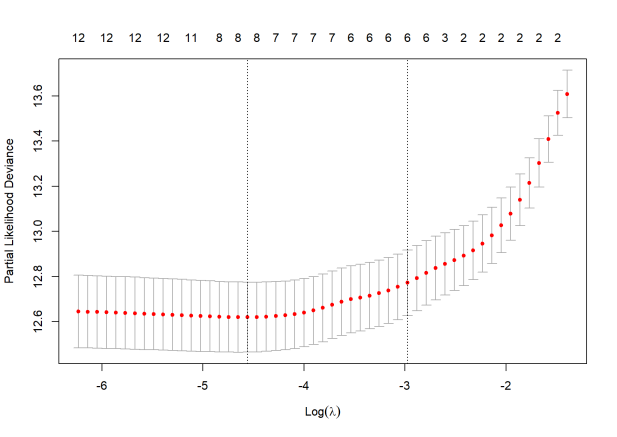 |
| Dataset 4 |  |
| 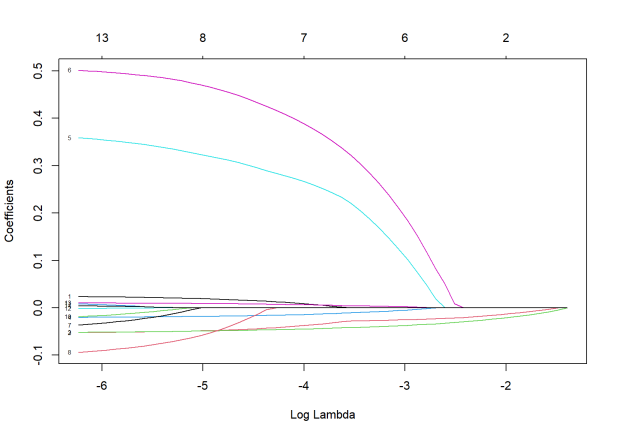 | 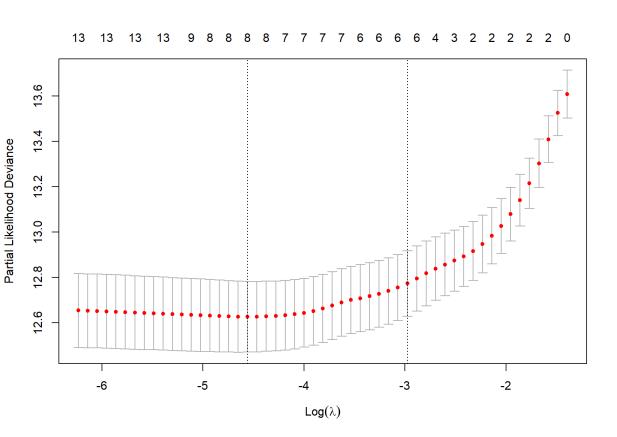 |
| Dataset 5 |  |
| 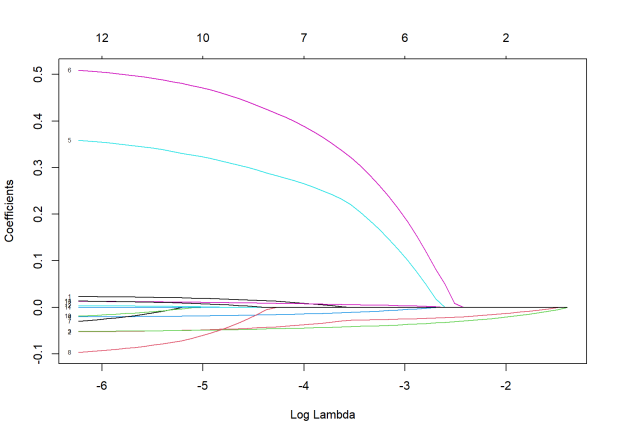 | 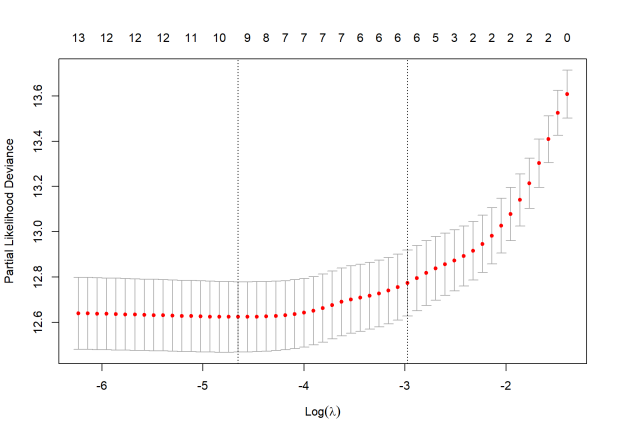 |

Figure1 Lasso selection of Exposure 1

| Dataset 1 |  |
| --- | --- |
| 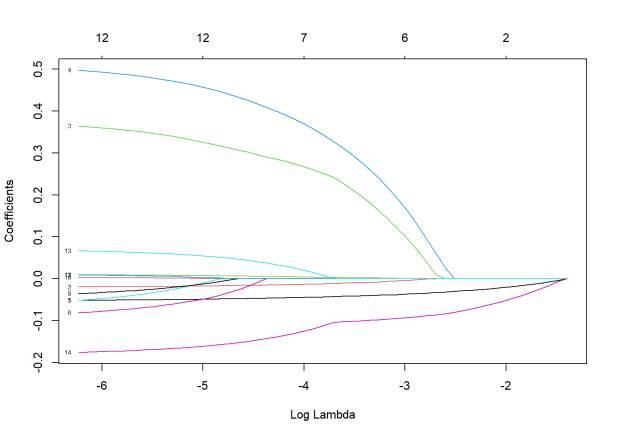 | 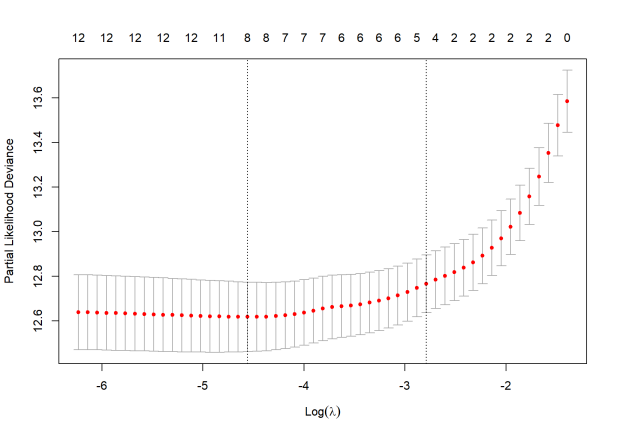 |
| Dataset 2 |  |
| 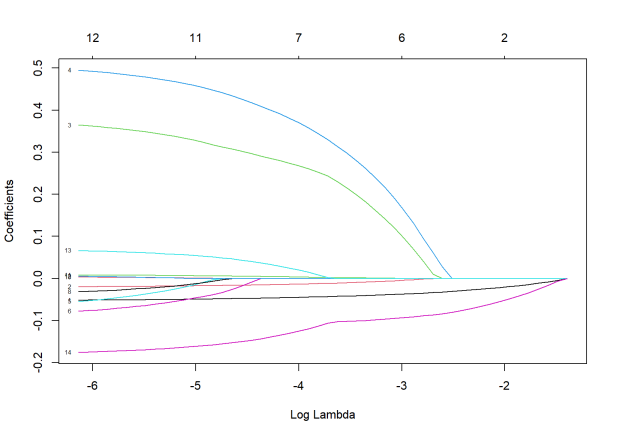 | 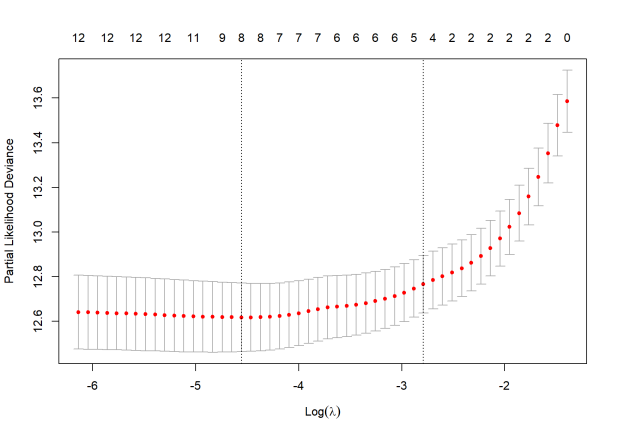 |
| Dataset 3 |  |
| 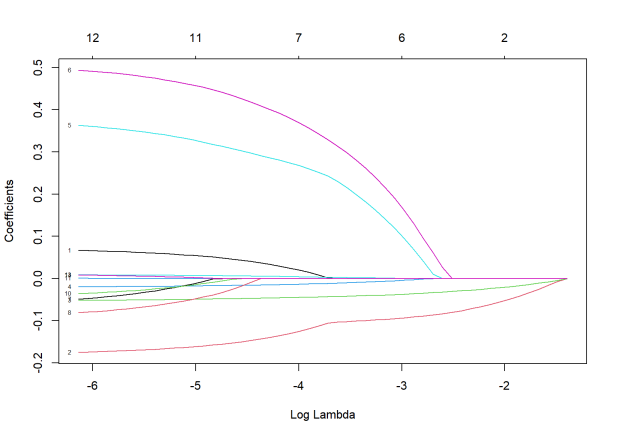 | 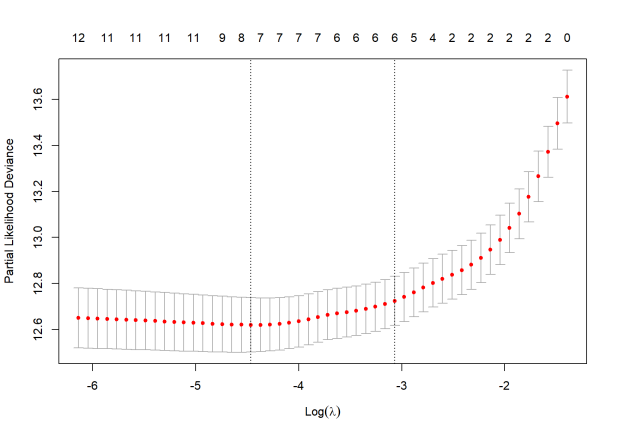 |
| Dataset 4 |  |
| 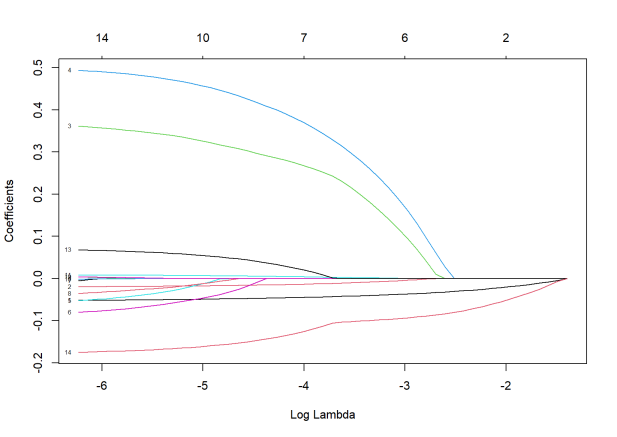 | 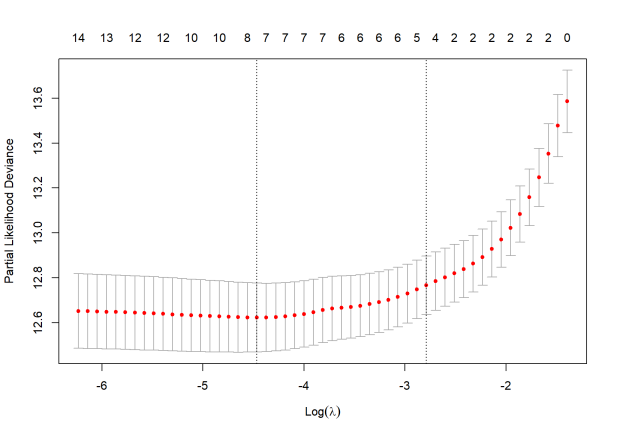 |
| Dataset 5 |  |
| 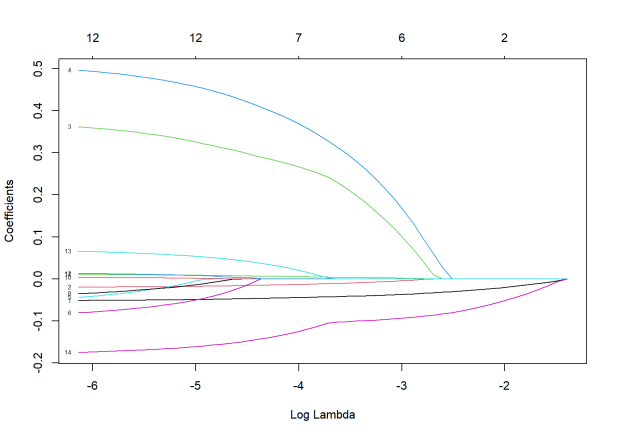 | 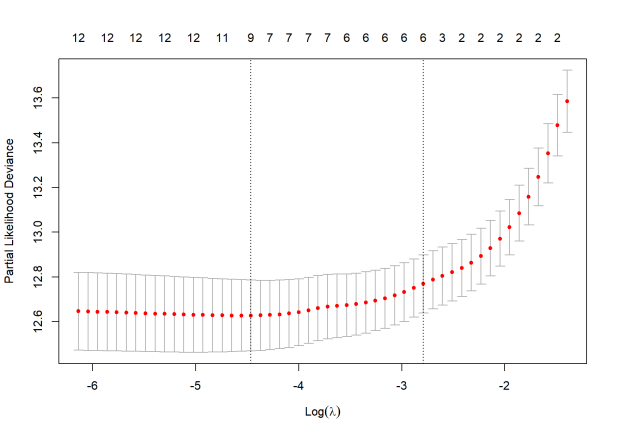 |

Figure2 Lasso selection of Exposure 2

Supplementary file 3.3 Results of Multivariate Cox regression in Datasets

Table 4 Multivariate Cox regression of Exposure 1

| Variables | HR(95%CI) | *P* | AIC |
| --- | --- | --- | --- |
| Dataset 1 |  |  |  |
| mean_hb, g/L | 0.851(0.786,0.921) | 0.000 | 2878.773 |
| Age, year | 0.981(0.973,0.990) | 0.000 |  |
| eGFR, ml/min/1.73 m^2^ | 0.952(0.941,0.963) | <0.000 |  |
| Urea, mmol/L | 1.002(0.976,1.028) | 0.897 |  |
| With Hypertension | 1.525(1.098,2.119) | 0.012 |  |
| With Diabetes | 1.651(1.255,2.172) | 0.000 |  |
| Dataset 2 |  |  |  |
| mean_hb, g/L | 0.851(0.786,0.921) | 0.000 | 2878.781 |
| Age, year | 0.981(0.973,0.990) | 0.000 |  |
| eGFR, ml/min/1.73 m^2^ | 0.952(0.941,0.963) | <0.000 |  |
| Urea, mmol/L | 1.001(0.976,1.028) | 0.925 |  |
| With Hypertension | 1.526(1.098,2.120) | 0.012 |  |
| With Diabetes | 1.651(1.255,2.172) | 0.000 |  |
| Dataset 3 |  |  |  |
| mean_hb, g/L | 0.851(0.786,0.921) | 0.000 | 2878.762 |
| Age, year | 0.981(0.973,0.990) | 0.000 |  |
| eGFR, ml/min/1.73 m^2^ | 0.952(0.941,0.963) | <0.000 |  |
| Urea, mmol/L | 1.002(0.977,1.029) | 0.866 |  |
| With Hypertension | 1.525(1.098,2.119) | 0.012 |  |
| With Diabetes | 1.651(1.255,2.172) | 0.000 |  |
| Dataset 4 |  |  |  |
| mean_hb, g/L | 0.851(0.786,0.921) | 0.000 | 2878.777 |
| Age, year | 0.981(0.973,0.990) | 0.000 |  |
| eGFR, ml/min/1.73 m^2^ | 0.952(0.941,0.963) | <0.000 |  |
| Urea, mmol/L | 1.002(0.976,1.028) | 0.908 |  |
| With Hypertension | 1.526(1.098,2.120) | 0.012 |  |
| With Diabetes | 1.651(1.255,2.171) | 0.000 |  |
| Dataset 5 |  |  |  |
| mean_hb, g/L | 0.851(0.786,0.921) | 0.000 | 2878.756 |
| Age, year | 0.981(0.973,0.990) | 0.000 |  |
| eGFR, ml/min/1.73 m^2^ | 0.952(0.941,0.963) | <0.000 |  |
| Urea, mmol/L | 1.002(0.977,1.029) | 0.853 |  |
| With Hypertension | 1.524(1.097,2.118) | 0.012 |  |
| With Diabetes | 1.651(1.255,2.171) | 0.000 |  |

Note:hemoglobin, hb; estimated glomerular filtration rate, eGFR.

Table 5 Multivariate Cox regression of exposure 2

|  | HR(95%CI) | *P* | AIC |
| --- | --- | --- | --- |
| Dataset 1 |  |  |  |
| mean_hct, % | 0.578(0.442,0.758) | 0.000 | 2870.690 |
| Age, year | 0.982(0.974,0.991) | 0.000 |  |
| eGFR, ml/min/1.73 m^2^ | 0.952(0.941,0.962) | <0.000 |  |
| With Hypertension | 1.519(1.094,2.109) | 0.013 |  |
| With Diabetes | 1.611(1.223,2.123) | 0.001 |  |
| Dataset 2 |  |  |  |
| mean_hct, % | 0.578(0.442,0.758) | 0.000 | 2870.690 |
| Age, year | 0.982(0.974,0.991) | 0.000 |  |
| eGFR, ml/min/1.73 m^2^ | 0.952(0.941,0.962) | <0.000 |  |
| With Hypertension | 1.519(1.094,2.109) | 0.013 |  |
| With Diabetes | 1.611(1.223,2.123) | 0.001 |  |
| Dataset 3 |  |  |  |
| mean_hct, % | 0.577(0.439,0.759) | 0.000 | 2872.681 |
| Age, year | 0.982(0.974,0.991) | 0.000 |  |
| eGFR, ml/min/1.73 m^2^ | 0.951(0.940,0.963) | <0.000 |  |
| Urea, mmol/L | 0.999(0.973,1.025) | 0.928 |  |
| With Hypertension | 1.520(1.094,2.112) | 0.013 |  |
| With Diabetes | 1.611(1.223,2.122) | 0.001 |  |
| Dataset 4 |  |  |  |
| mean_hct, % | 0.578(0.442,0.758) | 0.000 | 2870.690 |
| Age, year | 0.982(0.974,0.991) | 0.000 |  |
| eGFR, ml/min/1.73 m^2^ | 0.952(0.941,0.962) | <0.000 |  |
| With Hypertension | 1.519(1.094,2.109) | 0.013 |  |
| With Diabetes | 1.611(1.223,2.123) | 0.001 |  |
| Dataset 5 |  |  |  |
| mean_hct, % | 0.578(0.440,0.759) | 0.000 | 2872.685 |
| Age, year | 0.982(0.974,0.991) | 0.000 |  |
| eGFR, ml/min/1.73 m^2^ | 0.951(0.941,0.963) | <0.000 |  |
| Urea, mmol/L | 0.999(0.974,1.025) | 0.947 |  |
| With Hypertension | 1.520(1.094,2.112) | 0.013 |  |
| With Diabetes | 1.611(1.223,2.123) | 0.001 |  |

Note:hematocrit, hct; estimated glomerular filtration rate, eGFR.

Supplementary file 3.4 Pooled results of Multivariate Cox regression

Table 6 Pooled results of exposure 1

| Variables | HR(95%CI) | *P* |
| --- | --- | --- |
| mean_hb, g/L | 0.851(0.786,0.921) | 0.000 |
| Age, year | 0.981(0.973,0.990) | 0.000 |
| eGFR, ml/min/1.73 m^2^ | 0.952(0.941,0.963) | 0.000 |
| Urea, mmol/L | 1.002(0.976,1.028) | 0.890 |
| With Hypertension | 1.525(1.098,2.119) | 0.012 |
| With Diabetes | 1.651(1.255,2.172) | 0.000 |

Note:hemoglobin, hb; estimated glomerular filtration rate, eGFR.

Table 7 Pooled results of exposure 2

| Variables | HR(95%CI) | *P* |
| --- | --- | --- |
| mean_hct, % | 0.578(0.441,0.758) | 0.000 |
| Age, year | 0.982(0.974,0.991) | 0.000 |
| eGFR, ml/min/1.73 m^2^ | 0.952(0.941,0.962) | 0.000 |
| Urea, mmol/L | 0.999(0.974,1.025) | 0.938 |
| With Hypertension | 1.519(1.094,2.110) | 0.013 |
| With Diabetes | 1.611(1.259,2.062) | 0.000 |

Note:hematocrit, hct; estimated glomerular filtration rate, eGFR.
