# Supplementary material for: Time-updated patterns of hemoglobin and hematocrit and the risk of CKD progression
Source: Front Endocrinol (Lausanne). 2025 Oct 30;16:1642307. doi: 10.3389/fendo.2025.1642307 (PMC12611651; doi:10.3389/fendo.2025.1642307)
Supplement: Supplementary file 4 [file DataSheet4.docx]

**Supplementary file 4**

The statistical criteria and model fit indices included Bayesian information criterion (BIC), average posterior probability (AvepP), probability of group membership (πj), posterior probability of group membership (Pj), odds of correct classification (OCC) and relative entropy (Ek). A BIC closer to zero indicates a better model fitness. An AvepP of more than 70% over all the subgroups of the trajectories is considered as an indication of an adequate classification. The closer πj(%) is to Pj(%), the better the model is generally considered to fit the data. A minimum membership of 5% is required in each specific trajectory group. An OCC greater than 5 indicates high model classification accuracy. An Ek greater than 0.8 indicates lower classification uncertainty.

Supplementary file 4.1 Selection of trajectory clusters of Hb

| Group | Parameter | *Avepp(%)* | *OCC* | *P_j_(%)* | *π_j_(%)* | *BIC^#2^* | *△BIC^#2^* | *E_k_* |
| --- | --- | --- | --- | --- | --- | --- | --- | --- |
| *1Group* | *Linear* | 100.00 | . | 100.00 | 100.00 | -18965.99 |  | 0.000 |
|  | *Quadratic* | 100.00 | . | 100.00 | 100.00 | -18968.91 | -2.92 | 0.000 |
|  | *Cubic* | 100.00 | . | 100.00 | 100.00 | -18971.26 | -2.35 | 0.000 |
| *2Group* | *Linear* | 95.62-95.75 | 15.1-32.7 | 60.14-39.86 | 59.20-40.80 | -18027.98 | 943.28 | 0.851 |
|  | *Quadratic* | 95.54-95.75 | 15.0-32.3 | 59.86-40.14 | 58.90-41.10 | -18030.96 | -2.98 | 0.850 |
|  | *Cubic* | 95.74-95.39 | 15.6-29.8 | 59.73-40.27 | 59.04-40.96 | -18033.31 | -2.36 | 0.850 |
| *3Group* | *Linear* | 93.44-92.89-94.78 | 30.1-14.7-69.1 | 32.19-47.26-20.55 | 32.09-47.08-20.83 | -17630.06 | 403.25 | 0.854 |
|  | *Quadratic* | 93.63-92.47-95.34 | 30.8-13.8-78.8 | 32.19-47.67-20.14 | 32.34-47.07-20.59 | -17634.11 | -4.05 | 0.853 |
|  | *Cubic* | 93.39-92.63-95.43 | 29.5-14.3-79.8 | 32.47-47.26-20.27 | 32.41-46.85-20.74 | -17638.25 | -4.14 | 0.854 |
| *4Group* | ***Linear*** | **90.07-85.35-87.86-93.84** | **40.9-11.4-15.2-81.2** | **17.67-34.38-32.47-15.48** | **18.15-33.77-32.29-15.79** | **-17484.10** | **154.15** | **0.806** |
|  | *Quadratic* | 90.23-84.97-87.78-94.01 | 41.5-11.2-15.0-82.2 | 17.67-34.11-32.47-15.75 | 18.19-33.45-32.33-16.03 | -17494.67 | -10.58 | 0.805 |
|  | *Cubic* | 90.20-84.58-88.59-94.25 | 40.8-10.8-16.3-88.6 | 17.95-34.93-31.92-15.21 | 18.39-33.66-32.34-15.61 | -17501.14 | -6.47 | 0.806 |
| *5Group* | *Linear* | 81.27-82.29-84.56-87.08-93.39 | 50.3-15.7-11.4-21.3-96.8 | 7.95-22.88-33.15-23.70-12.33 | 7.94-22.83-32.44-24.05-12.74 | -17426.05 | 75.09 | 0.785 |
|  | *Quadratic* | 82.71-81.56-85.16-87.05-93.00 | 54.3-15.1-12.0-20.7-93.2 | 8.08-23.15-32.19-24.38-12.19 | 8.09-22.64-32.29-24.49-12.49 | -17434.22 | -8.17 | 0.786 |
|  | *Cubic* | 89.14-85.52-86.08-89.19-87.31 | 46.6-13.2-13.1-38.9-144.7 | 14.52-30.96-32.88-17.12-4.52 | 14.99-30.97-32.01-17.50-4.54 | -17446.57 | -12.35 | 0.810 |
| *6Group* | *Linear* | 80.83-81.39-81.90-84.65-89.67-92.77 | 50.1-15.9-10.6-17.9-51.0-477.2 | 7.95-21.64-30.68-22.74-14.52-2.47 | 7.76-21.56-29.98-23.54-14.56-2.62 | -17379.50 | 67.07 | 0.788 |
|  | *Quadratic* | 83.34-79.54-81.31-83.97-87.43-89.71 | 57.7-14.6-10.8-16.8-39.4-247.3 | 7.95-22.05-28.36-23.01-15.48-3.15 | 7.97-21.07-28.76-23.79-15.00-3.40 | -17390.10 | -10.60 | 0.780 |
|  | *Cubic* | 81.41-80.39-81.08-83.69-87.48-92.34 | 53.2-15.2-10.8-16.3-39.2-326.0 | 7.95-21.92-28.08-23.29-15.62-3.15 | 7.61-21.26-28.47-23.96-15.14-3.57 | -17407.07 | -16.97 | 0.780 |

Supplementary file 4.2 Selection of trajectory clusters of HCT

| Group | Parameter | *Avepp(%)* | *OCC* | *P_j_(%)* | *π_j_(%)* | *BIC^#2^* | *△BIC^#2^* | *E_k_* |
| --- | --- | --- | --- | --- | --- | --- | --- | --- |
| *1Group* | *Linear* | 100.00 | . | 100.00 | 100.00 | -13672.16 |  | 0.000 |
|  | *Quadratic* | 100.00 | . | 100.00 | 100.00 | -13674.88 | -2.72 | 0.000 |
|  | *Cubic* | 100.00 | . | 100.00 | 100.00 | -13677.94 | -3.06 | 0.000 |
| *2Group* | *Linear* | 95.00-95.77 | 15.6-27.5 | 55.75-44.25 | 54.84-45.16 | -12739.41 | 938.53 | 0.847 |
|  | *Quadratic* | 95.24-95.51 | 16.7-25.5 | 55.07-44.93 | 54.46-45.54 | -12741.92 | -2.51 | 0.847 |
|  | *Cubic* | 95.37-95.25 | 17.1-24.2 | 55.07-44.93 | 54.66-45.34 | -12746.40 | -4.48 | 0.847 |
| *3Group* | *Linear* | 93.75-91.41-93.81 | 31.7-12.7-53.1 | 31.51-46.30-22.19 | 32.13-45.67-22.21 | -12376.66 | 369.74 | 0.842 |
|  | *Quadratic* | 93.68-90.95-94.50 | 31.2-12.0-60.5 | 31.51-46.71-21.78 | 32.22-45.68-22.11 | -12381.96 | -5.30 | 0.841 |
|  | *Cubic* | 93.61-90.97-94.60 | 30.7-12.0-61.5 | 31.64-46.58-21.78 | 32.27-45.57-22.17 | -12390.20 | -8.24 | 0.841 |
| *4Group* | ***Linear*** | **91.11-85.62-87.60-92.01** | **33.5-11.3-17.4-76.2** | **23.56-35.48-28.22-12.74** | **23.43-34.56-28.89-13.13** | **-12237.32** | **152.87** | **0.804** |
|  | *Quadratic* | 91.10-85.19-87.32-92.11 | 33.3-11.2-16.8-75.9 | 23.70-34.93-28.49-12.88 | 23.53-34.00-29.14-13.33 | -12247.05 | -9.72 | 0.802 |
|  | *Cubic* | 91.18-85.19-87.90-92.19 | 33.6-11.2-17.5-77.3 | 23.70-34.93-28.49-12.88 | 23.55-33.91-29.29-13.25 | -12257.08 | -10.03 | 0.804 |
| *5Group* | *Linear* | 83.83-80.75-82.23-86.13-92.87 | 50.3-14.5-10.2-18.5-96.5 | 8.90-22.05-32.47-25.21-11.37 | 9.34-22.40-31.25-25.12-11.89 | -12187.80 | 69.29 | 0.772 |
|  | *Quadratic* | 84.82-81.13-81.45-86.09-92.65 | 53.7-14.9-9.8-18.3-93.6 | 9.04-21.78-32.47-25.34-11.37 | 9.43-22.41-30.98-25.29-11.88 | -12200.39 | -12.60 | 0.771 |
|  | *Cubic* | 85.55-81.18-81.64-86.65-92.00 | 58.6-14.7-9.9-19.2-85.9 | 8.63-22.47-32.33-25.07-11.51 | 9.17-22.74-30.98-25.31-11.81 | -12214.25 | -13.86 | 0.774 |
| *6Group* | *Linear* | 83.93-78.88-79.41-83.83-83.29-94.26 | 54.7-14.8-9.5-15.7-31.2-431.7 | 8.22-20.14-30.68-23.56-14.25-3.15 | 8.72-20.13-28.92-24.80-13.76-3.66 | -12160.05 | 54.20 | 0.766 |
|  | *Quadratic* | 85.36-86.30-83.29-87.59-84.43-92.07 | 129.2-21.2-10.6-19.3-377.0-80.5 | 3.97-23.01-33.42-26.30-0.96-12.33 | 4.32-22.87-31.98-26.81-1.42-12.61 | -12158.67 | 1.38 | 0.816 |
|  | *Cubic* | 85.06-79.70-79.79-82.73-84.55-95.31 | 66.5-16.1-10.2-14.1-29.8-524.1 | 7.26-19.73-28.77-24.93-16.16-3.15 | 7.88-19.60-27.83-25.42-15.53-3.73 | -12191.71 | -33.04 | 0.770 |

For trajectory group modelling of Hb, the trajectories clustered into 5 to 6 groups exhibited better BIC values but undesirable E_k_ (less than 0.80) in linear and squared form in 5 groups and all forms in 6 groups. One of the trajectories of cubic form in 5 groups and all forms in 6 groups exhibited the P_j_(%) and π_j_(%) less than 5. For trajectory group modelling of HCT, the trajectories clustered into 5 to 6 groups also exhibited better BIC values. The E_k_ in all forms in 5 groups and linear and cubic forms in 6 groups were less than 0.80. All forms of trajectories clustered into 6 groups had one trajectory exhibiting a P_j_(%) and π_j_(%) below 5. Compare to other forms of 1 to 4 groups, the linear form of the trajectories clustered into 4 groups in GBTM models of Hb and HCT exhibited superior BIC with an E_k_ greater than 0.80 and the P_j_(%) and π_j_(%) greater than 5. Compare to other forms of 1 to 4 groups, the linear form of the trajectories clustered into 4 groups in GBTM models of Hb and HCT exhibited superior BIC with an E_k_ greater than 0.80 and the P_j_(%) and π_j_(%) greater than 5. It indicated that the linear form of 4 groups had desirable fitness, a reasonable distribution and an accurate classification. Hence, the linear form of 4 groups was selected as the optimal model. The optimal model's trajectories were visualised in Figure 4.

Supplementary file 4.3 Characteristics of trajectories

Table 1 Baseline characteristics of the total sample and the sample by Hb trajectory group

|  |  | lower and decreasing (N=129) | lower and growing slightly (N=251) | higher and growing slightly (N=237) | | higher and growing steadily (N=113) | *P* |
| --- | --- | --- | --- | --- | --- | --- | --- |
| Variables |  | Mean (SD)/Median (IQR)/N(%) | | |  | | |
| baseline_Hb, g/L |  | 98.00 (90.00, 107.00) | 114.00 (106.00, 121.00) | 130.00 (121.00, 136.00) | | 150.00 (140.00, 157.00) | <0.001 |
| mean_Hb, g/L |  | 95.29 (87.33, 101.00) | 111.54 (105.73, 118.45) | 128.00 (123.33, 134.33) | | 148.22 (142.29, 155.67) | <0.001 |
| baseline_HCT, % |  | 30.30 (27.50, 32.70) | 34.80 (32.30, 36.75) | 39.40 (37.20, 41.30) | | 44.40 (42.30, 46.80) | <0.001 |
| mean_HCT, % |  | 29.09 (26.77, 31.08) | 34.12 (32.12, 36.01) | 38.83 (37.33, 40.67) | | 44.50 (42.82, 46.26) | <0.001 |
| Age, year |  | 60.44 (50.67, 70.62) | 60.57 (49.52, 68.32) | 58.76 (49.18, 69.11) | | 57.13 (44.52, 65.65) | 0.046 |
| Sex | Female | 79 (61.24) | 138 (54.98) | 84 (35.44) | | 5 (4.42) | <0.001 |
|  | Male | 50 (38.76) | 113 (45.02) | 153 (64.56) | | 108 (95.58) |  |
| eGFR, ml/min/1.73 m^2^ |  | 28.57 (22.09, 44.34) | 34.56 (23.44, 47.16) | 43.03 (29.33, 51.54) | | 47.72 (39.28, 55.43) | <0.001 |
| ALB, g/L |  | 37.40 (31.55, 41.20) | 39.70 (33.73, 43.05) | 42.20 (38.85, 44.90) | | 44.80 (41.00, 46.95) | <0.001 |
| Urea, mmol/L |  | 11.01 (8.17, 14.16) | 9.84 (8.08, 12.42) | 8.91 (6.94, 10.84) | | 7.45 (6.34, 8.89) | <0.001 |
| UA, mmol/L |  | 469.50 (393.75, 538.00) | 449.95 (369.50, 544.25) | 451.00 (384.50, 516.00) | | 470.40 (393.00, 529.00) | 0.487 |
| TCO2, mmol/L |  | 22.30 (20.80, 25.02) | 23.40 (21.30, 26.30) | 23.90 (22.10, 26.05) | | 24.50 (22.70, 26.05) | <0.001 |
| LDL-C, mmol/L |  | 3.08 (2.25, 4.05) | 3.40 (2.46, 4.56) | 3.12 (2.51, 4.14) | | 3.21 (2.46, 3.99) | 0.243 |
| TC, mmol/L |  | 4.83 (3.81, 6.21) | 5.10 (4.12, 6.60) | 5.08 (4.30, 6.15) | | 4.86 (4.34, 5.68) | 0.414 |
| HDL-C, mmol/L |  | 1.23 (0.93, 1.52) | 1.18 (0.96, 1.44) | 1.14 (0.96, 1.44) | | 1.12 (0.94, 1.38) | 0.424 |
| AST, mmol/L |  | 18.00 (15.00, 22.00) | 18.00 (14.00, 23.00) | 19.00 (16.00, 25.00) | | 20.00 (17.00, 25.00) | 0.009 |
| ALT, mmol/L |  | 13.00 (9.00, 17.00) | 12.00 (10.00, 17.00) | 16.00 (12.00, 21.00) | | 20.00 (14.00, 28.00) | <0.001 |
| Protopathy | Primary Glomerulonephritides | 31 (24.03) | 71 (28.29) | 66 (27.85) | | 36 (31.86) | 0.022 |
|  | Hypertensive Renal Disease | 2 (1.55) | 7 (2.79) | 5 (2.11) | | 5 (4.42) |  |
|  | Diabetic nephropathy | 11 (8.53) | 16 (6.37) | 12 (5.06) | | 1 (0.88) |  |
|  | Others | 6 (4.65) | 13 (5.18) | 30 (12.66) | | 13 (11.50) |  |
|  | Unknown | 79 (61.24) | 144 (57.37) | 124 (52.32) | | 58 (51.33) |  |
| With Hypertension | No | 23 (17.83) | 53 (21.12) | 78 (32.91) | | 40 (35.40) | <0.001 |
|  | Yes | 106 (82.17) | 198 (78.88) | 159 (67.09) | | 73 (64.60) |  |
| With Diabetes | No | 66 (51.16) | 162 (64.54) | 175 (73.84) | | 93 (82.30) | <0.001 |
|  | Yes | 63 (48.84) | 89 (35.46) | 62 (26.16) | | 20 (17.70) |  |
| With Hyperuricemia | No | 120 (93.02) | 221 (88.05) | 221 (93.25) | | 99 (87.61) | 0.116 |
|  | Yes | 9 (6.98) | 30 (11.95) | 16 (6.75) | | 14 (12.39) |  |
| With Hyperlipidemia | No | 102 (79.07) | 211 (84.06) | 210 (88.61) | | 96 (84.96) | 0.109 |
|  | Yes | 27 (20.93) | 40 (15.94) | 27 (11.39) | | 17 (15.04) |  |
| With Anemia | No | 114 (88.37) | 242 (96.41) | 229 (96.62) | | 112 (99.12) | <0.001 |
|  | Yes | 15 (11.63) | 9 (3.59) | 8 (3.38) | | 1 (0.88) |  |
| With ACEI/ARB | No | 75 (58.14) | 141 (56.18) | 147 (62.03) | | 68 (60.18) | 0.609 |
|  | Yes | 54 (41.86) | 110 (43.82) | 90 (37.97) | | 45 (39.82) |  |
| With Calcium Supplements | No | 84 (65.12) | 191 (76.10) | 197 (83.12) | | 92 (81.42) | 0.001 |
|  | Yes | 45 (34.88) | 60 (23.90) | 40 (16.88) | | 21 (18.58) |  |
| With Sodium Bicarbonate | No | 76 (58.91) | 163 (64.94) | 160 (67.51) | | 67 (59.29) | 0.274 |
|  | Yes | 53 (41.09) | 88 (35.06) | 77 (32.49) | | 46 (40.71) |  |
| With Ketoacid Tablets | No | 80 (62.02) | 175 (69.72) | 170 (71.73) | | 82 (72.57) | 0.215 |
|  | Yes | 49 (37.98) | 76 (30.28) | 67 (28.27) | | 31 (27.43) |  |
| With Diuretics | No | 81 (62.79) | 182 (72.51) | 196 (82.70) | | 100 (88.50) | <0.001 |
|  | Yes | 48 (37.21) | 69 (27.49) | 41 (17.30) | | 13 (11.50) |  |
| With ESAs or Iron | No | 76(58.91) | 194(77.29) | 210(88.61) | | 107(94.69) | <0.001 |
|  | Yes | 53(41.09) | 57(22.71) | 27(11.39) | | 6(5.31) |  |
| Follow-up duration |  | 17.44 (11.08, 31.74) | 32.82 (14.48, 54.38) | 44.36 (24.79, 64.95) | | 47.28 (28.75, 62.26) | <0.001 |
| Composite outcomes (%) | No | 46 (35.66) | 143 (56.97) | 182 (76.79) | | 102 (90.27) | <0.001 |
|  | Yes | 83 (64.34) | 108 (43.03) | 55 (23.21) | | 11 (9.73) |  |

Note: estimated glomerular filtration rate, eGFR; albumin, ALB; uric acid, UA; total carbon dioxide, TCO2; low-density lipoprotein cholesterol, LDL-C; total cholesterol, TC; high-density lipoprotein cholesterol, HDL-C; aspartate transaminase, AST; alanine aminotransferase, ALT; angiotensin converting enzyme inhibitors, ACEI; Angiotensin receptor blocker, ARB;Primary Glomerulonephritides included chronic nephritis, nephropathy syndrome and IgA nephropathy.Other secondary nephrosis included systemic lupus erythematosus nephritis, Henoch-Schonlein purpura,Hepatitis B virus-associated nephritis and obstructive nephropathy, etc.; hemoglobin, Hb; hematocrit, HCT.

Table 2 Baseline characteristics of the total sample and the sample by HCT trajectory group

|  |  | lower and decreasing (N=129) | lower and growing slightly (N=251) | higher and growing slightly (N=237) | | higher and growing steadily (N=113) | *P* |
| --- | --- | --- | --- | --- | --- | --- | --- |
| Variables |  | Mean (SD)/Median (IQR)/N(%) | | |  | | |
| baseline_Hb, g/L |  | 98.00 (90.00, 107.00) | 114.00 (106.00, 121.00) | 130.00 (121.00, 136.00) | | 150.00 (140.00, 157.00) | <0.001 |
| mean_Hb, g/L |  | 95.29 (87.33, 101.00) | 111.54 (105.73, 118.45) | 128.00 (123.33, 134.33) | | 148.22 (142.29, 155.67) | <0.001 |
| baseline_HCT, % |  | 30.30 (27.50, 32.70) | 34.80 (32.30, 36.75) | 39.40 (37.20, 41.30) | | 44.40 (42.30, 46.80) | <0.001 |
| mean_HCT, % |  | 29.09 (26.77, 31.08) | 34.12 (32.12, 36.01) | 38.83 (37.33, 40.67) | | 44.50 (42.82, 46.26) | <0.001 |
| Age, year |  | 60.44 (50.67, 70.62) | 60.57 (49.52, 68.32) | 58.76 (49.18, 69.11) | | 57.13 (44.52, 65.65) | 0.046 |
| Sex | Female | 79 (61.24) | 138 (54.98) | 84 (35.44) | | 5 (4.42) | <0.001 |
|  | Male | 50 (38.76) | 113 (45.02) | 153 (64.56) | | 108 (95.58) |  |
| eGFR, ml/min/1.73 m^2^ |  | 28.57 (22.09, 44.34) | 34.56 (23.44, 47.16) | 43.03 (29.33, 51.54) | | 47.72 (39.28, 55.43) | <0.001 |
| ALB, g/L |  | 37.40 (31.55, 41.20) | 39.70 (33.73, 43.05) | 42.20 (38.85, 44.90) | | 44.80 (41.00, 46.95) | <0.001 |
| Urea, mmol/L |  | 11.01 (8.17, 14.16) | 9.84 (8.08, 12.42) | 8.91 (6.94, 10.84) | | 7.45 (6.34, 8.89) | <0.001 |
| UA, mmol/L |  | 469.50 (393.75, 538.00) | 449.95 (369.50, 544.25) | 451.00 (384.50, 516.00) | | 470.40 (393.00, 529.00) | 0.487 |
| TCO2, mmol/L |  | 22.30 (20.80, 25.02) | 23.40 (21.30, 26.30) | 23.90 (22.10, 26.05) | | 24.50 (22.70, 26.05) | <0.001 |
| LDL-C, mmol/L |  | 3.08 (2.25, 4.05) | 3.40 (2.46, 4.56) | 3.12 (2.51, 4.14) | | 3.21 (2.46, 3.99) | 0.243 |
| TC, mmol/L |  | 4.83 (3.81, 6.21) | 5.10 (4.12, 6.60) | 5.08 (4.30, 6.15) | | 4.86 (4.34, 5.68) | 0.414 |
| HDL-C, mmol/L |  | 1.23 (0.93, 1.52) | 1.18 (0.96, 1.44) | 1.14 (0.96, 1.44) | | 1.12 (0.94, 1.38) | 0.424 |
| AST, mmol/L |  | 18.00 (15.00, 22.00) | 18.00 (14.00, 23.00) | 19.00 (16.00, 25.00) | | 20.00 (17.00, 25.00) | 0.009 |
| ALT, mmol/L |  | 13.00 (9.00, 17.00) | 12.00 (10.00, 17.00) | 16.00 (12.00, 21.00) | | 20.00 (14.00, 28.00) | <0.001 |
| Protopathy | Primary Glomerulonephritides | 31 (24.03) | 71 (28.29) | 66 (27.85) | | 36 (31.86) | 0.022 |
|  | Hypertensive Renal Disease | 2 (1.55) | 7 (2.79) | 5 (2.11) | | 5 (4.42) |  |
|  | Diabetic nephropathy | 11 (8.53) | 16 (6.37) | 12 (5.06) | | 1 (0.88) |  |
|  | Others | 6 (4.65) | 13 (5.18) | 30 (12.66) | | 13 (11.50) |  |
|  | Unknown | 79 (61.24) | 144 (57.37) | 124 (52.32) | | 58 (51.33) |  |
| With Hypertension | No | 23 (17.83) | 53 (21.12) | 78 (32.91) | | 40 (35.40) | <0.001 |
|  | Yes | 106 (82.17) | 198 (78.88) | 159 (67.09) | | 73 (64.60) |  |
| With Diabetes | No | 66 (51.16) | 162 (64.54) | 175 (73.84) | | 93 (82.30) | <0.001 |
|  | Yes | 63 (48.84) | 89 (35.46) | 62 (26.16) | | 20 (17.70) |  |
| With Hyperuricemia | No | 120 (93.02) | 221 (88.05) | 221 (93.25) | | 99 (87.61) | 0.116 |
|  | Yes | 9 (6.98) | 30 (11.95) | 16 (6.75) | | 14 (12.39) |  |
| With Hyperlipidemia | No | 102 (79.07) | 211 (84.06) | 210 (88.61) | | 96 (84.96) | 0.109 |
|  | Yes | 27 (20.93) | 40 (15.94) | 27 (11.39) | | 17 (15.04) |  |
| With Anemia | No | 114 (88.37) | 242 (96.41) | 229 (96.62) | | 112 (99.12) | <0.001 |
|  | Yes | 15 (11.63) | 9 (3.59) | 8 (3.38) | | 1 (0.88) |  |
| With ACEI/ARB | No | 75 (58.14) | 141 (56.18) | 147 (62.03) | | 68 (60.18) | 0.609 |
|  | Yes | 54 (41.86) | 110 (43.82) | 90 (37.97) | | 45 (39.82) |  |
| With Calcium Supplements | No | 84 (65.12) | 191 (76.10) | 197 (83.12) | | 92 (81.42) | 0.001 |
|  | Yes | 45 (34.88) | 60 (23.90) | 40 (16.88) | | 21 (18.58) |  |
| With Sodium Bicarbonate | No | 76 (58.91) | 163 (64.94) | 160 (67.51) | | 67 (59.29) | 0.274 |
|  | Yes | 53 (41.09) | 88 (35.06) | 77 (32.49) | | 46 (40.71) |  |
| With Ketoacid Tablets | No | 80 (62.02) | 175 (69.72) | 170 (71.73) | | 82 (72.57) | 0.215 |
|  | Yes | 49 (37.98) | 76 (30.28) | 67 (28.27) | | 31 (27.43) |  |
| With Diuretics | No | 81 (62.79) | 182 (72.51) | 196 (82.70) | | 100 (88.50) | <0.001 |
|  | Yes | 48 (37.21) | 69 (27.49) | 41 (17.30) | | 13 (11.50) |  |
| With ESAs or Iron | No | 76(58.91) | 194(77.29) | 210(88.61) | | 107(94.69) | <0.001 |
|  | Yes | 53(41.09) | 57(22.71) | 27(11.39) | | 6(5.31) |  |
| Follow-up duration |  | 17.44 (11.08, 31.74) | 32.82 (14.48, 54.38) | 44.36 (24.79, 64.95) | | 47.28 (28.75, 62.26) | <0.001 |
| Composite outcomes (%) | No | 46 (35.66) | 143 (56.97) | 182 (76.79) | | 102 (90.27) | <0.001 |
|  | Yes | 83 (64.34) | 108 (43.03) | 55 (23.21) | | 11 (9.73) |  |

Note: estimated glomerular filtration rate, eGFR; albumin, ALB; uric acid, UA; total carbon dioxide, TCO2; low-density lipoprotein cholesterol, LDL-C; total cholesterol, TC; high-density lipoprotein cholesterol, HDL-C; aspartate transaminase, AST; alanine aminotransferase, ALT; angiotensin converting enzyme inhibitors, ACEI; Angiotensin receptor blocker, ARB;Primary Glomerulonephritides included chronic nephritis, nephropathy syndrome and IgA nephropathy.Other secondary nephrosis included systemic lupus erythematosus nephritis, Henoch-Schonlein purpura,Hepatitis B virus-associated nephritis and obstructive nephropathy, etc.; hemoglobin, Hb; hematocrit, HCT.

In comparison of baseline characteristics, it indicated that exposure 1, exposure 2, age, sex, eGFR, ALB, Urea, TCO2, AST, ALT, etiology, with hypertension, with diabetes mellitus, with anaemia, use of calcium supplements and use of diuretics were statistically significant between trajectories in both GBTM models of Hb and HCT (P< 0. 05)

Supplementary file 4.4 Cox regression of trajectory clusters

Table 3 Test for assumption, Univariate Cox regression of trajectory clusters

| Variables | Group | HR(95%CI) | *P* for univariate Cox regression | *P* for trend | *P* for Schoenfeld Individual Test |
| --- | --- | --- | --- | --- | --- |
| hb_group | Lower and decreasing | Ref |  | <0.000 | 0.129 |
|  | Lower and growing slightly | 0.429(0.321,0.573) | 0.000 |  |  |
|  | Higher and growing slightly | 0.184(0.130,0.261) | <0.000 |  |  |
|  | Higher and growing steadily | 0.075(0.040,0.142) | 0.000 |  |  |
| hct_group | Lower and decreasing | Ref |  | <0.000 | 0.100 |
|  | Lower and growing slightly | 0.390(0.297,0.512) | 0.000 |  |  |
|  | Higher and growing slightly | 0.143(0.098,0.208) | <0.000 |  |  |
|  | Higher and growing steadily | 0.059(0.027,0.126) | 0.000 |  |  |

| Dataset 1 |  |
| --- | --- |
| 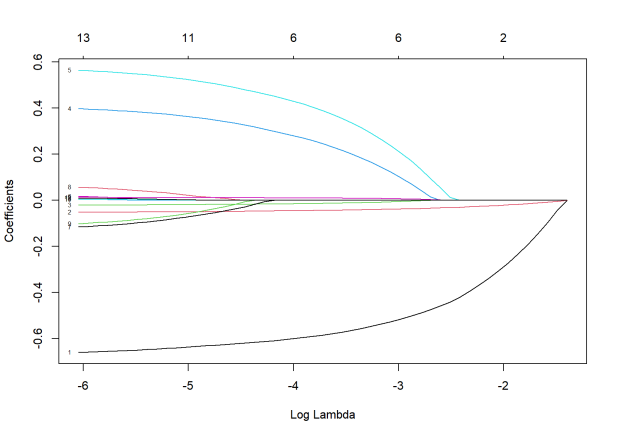 | 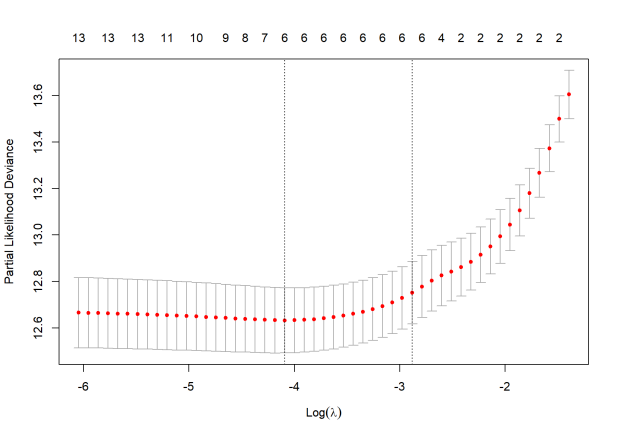 |
| Dataset 2 |  |
| 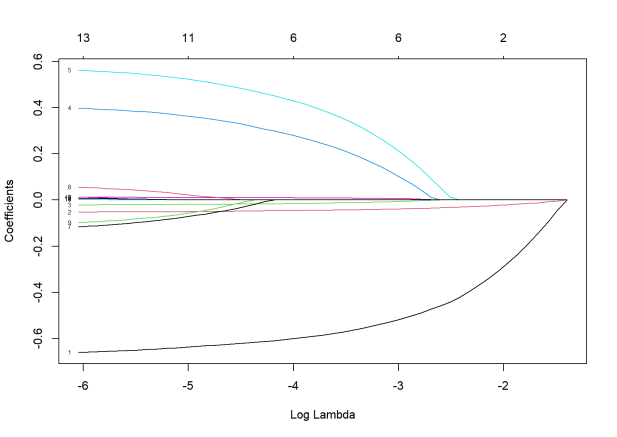 | 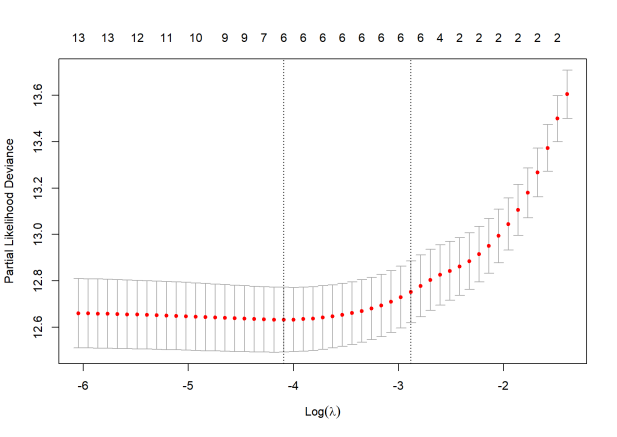 |
| Dataset 3 |  |
| 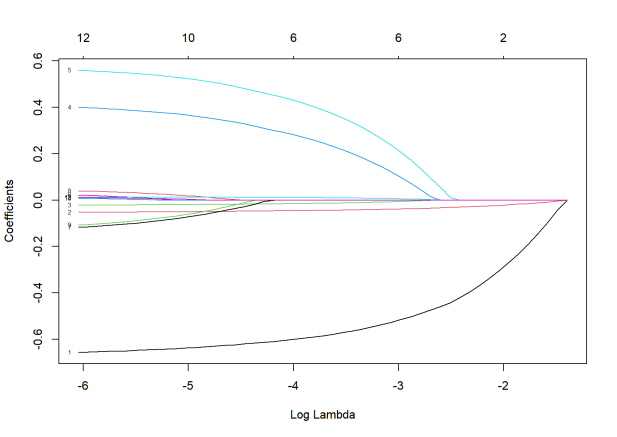 | 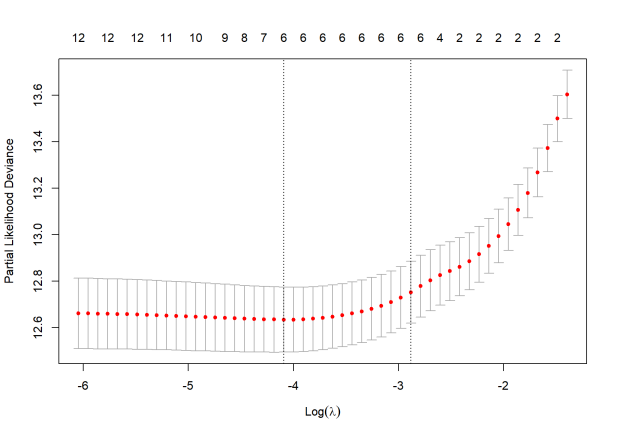 |
| Dataset 4 |  |
| 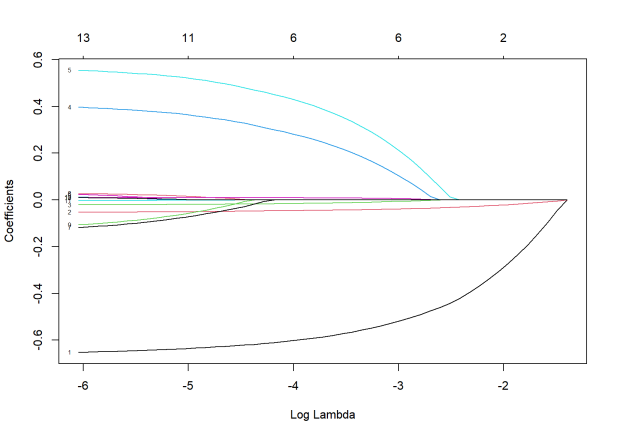 | 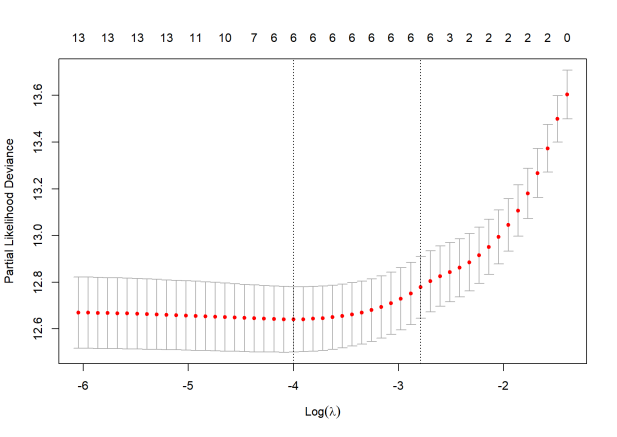 |
| Dataset 5 |  |
| 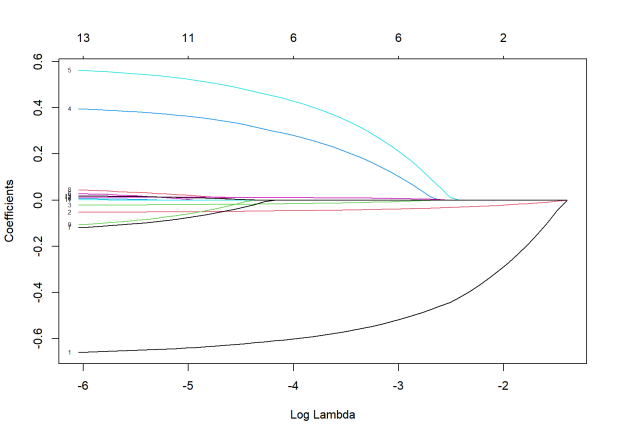 | 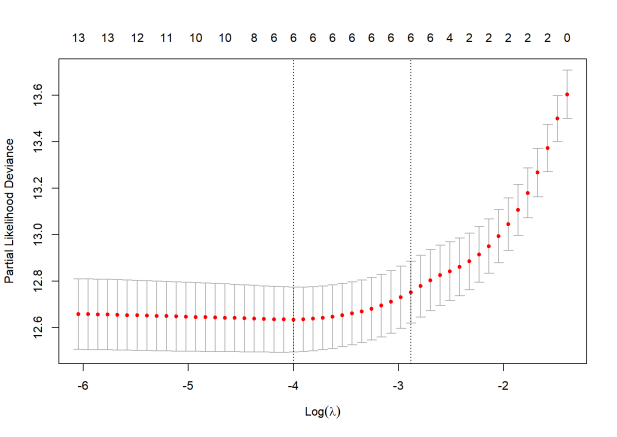 |

Figure1 Lasso selection of trajectories of Hb

| Dataset 1 |  |
| --- | --- |
| 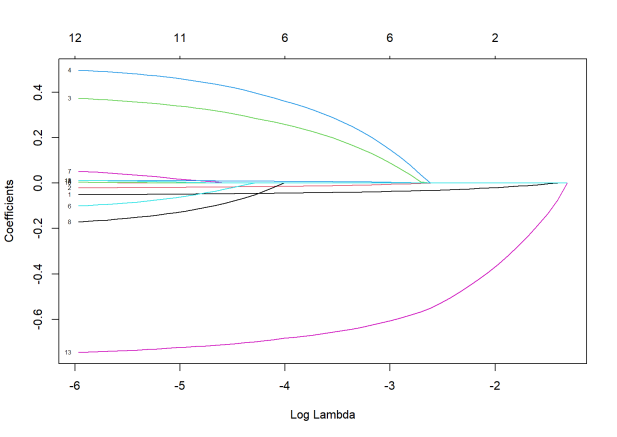 | 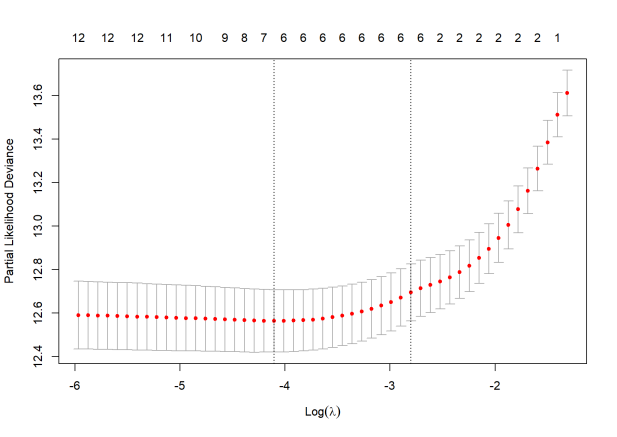 |
| Dataset 2 |  |
| 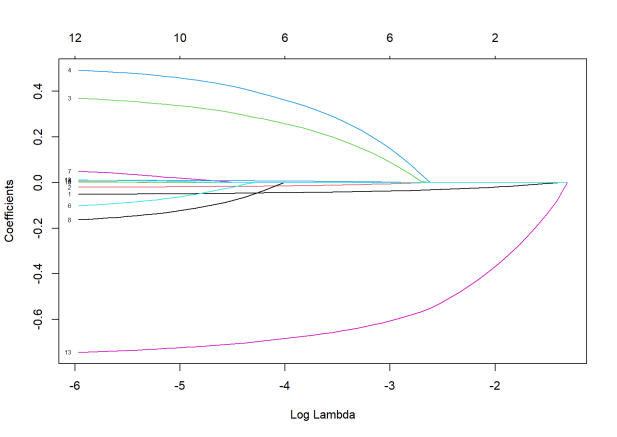 | 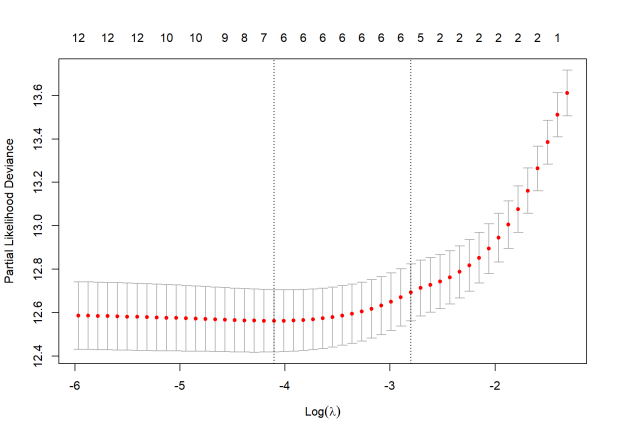 |
| Dataset 3 |  |
| 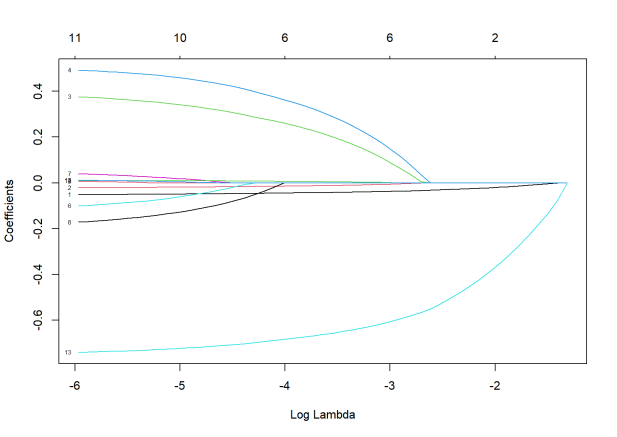 | 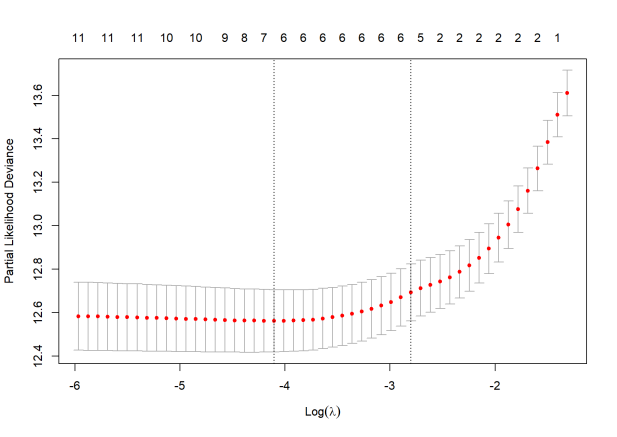 |
| Dataset 4 |  |
| 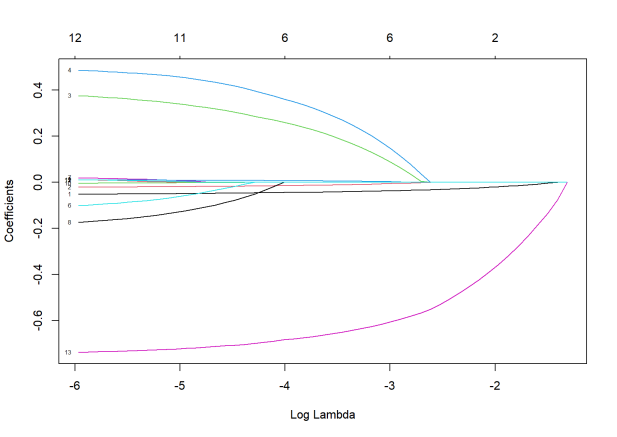 | 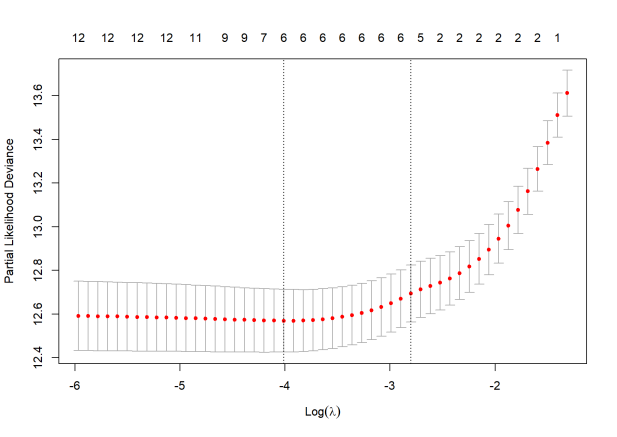 |
| Dataset 5 |  |
| 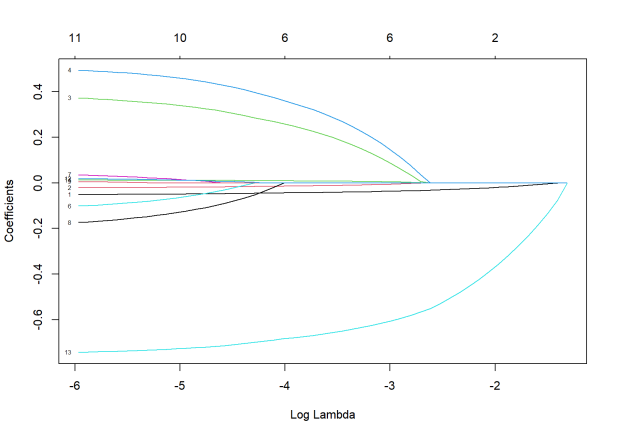 | 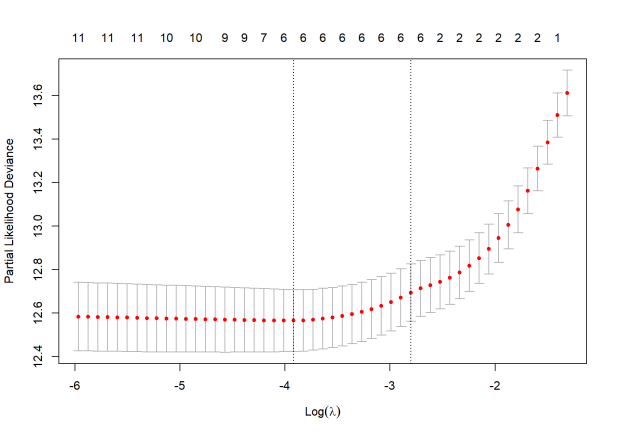 |

Figure2 Lasso selection of trajectories of HCT

Table 4 Multivariate Cox regression of trajectories of Hb

|  |  | HR(95%CI) | *P* | *P* for trend | AIC |
| --- | --- | --- | --- | --- | --- |
| Dataset 1 |  |  |  |  |  |
| hb_group | Lower and decreasing | Ref |  | <0.000 | 2877.589 |
|  | Lower and growing slightly | 0.545(0.407,0.731) | 0.000 |  |  |
|  | Higher and growing slightly | 0.268(0.187,0.383) | 0.000 |  |  |
|  | Higher and growing steadily | 0.143(0.075,0.276) | 0.000 |  |  |
| eGFR, ml/min/1.73 m^2^ |  | 0.950(0.939,0.962) | <0.000 |  |  |
| Age ,year |  | 0.979(0.971,0.988) | 0.000 |  |  |
| With Hypertension |  | 1.485(1.071,2.061) | 0.018 |  |  |
| With Diabetes |  | 1.753(1.333,2.305) | 0.000 |  |  |
| Urea, mmol/L |  | 1.011(0.985,1.039) | 0.407 |  |  |
| Dataset 2 |  |  |  |  |  |
| hb_group | Lower and decreasing | Ref |  | <0.000 | 2877.627 |
|  | Lower and growing slightly | 0.545(0.406,0.731) | 0.000 |  |  |
|  | Higher and growing slightly | 0.268(0.187,0.383) | 0.000 |  |  |
|  | Higher and growing steadily | 0.143(0.075,0.276) | 0.000 |  |  |
| eGFR, ml/min/1.73 m^2^ |  | 0.950(0.939,0.962) | <0.000 |  |  |
| Age, year |  | 0.979(0.971,0.988) | 0.000 |  |  |
| With Hypertension |  | 1.486(1.071,2.061) | 0.018 |  |  |
| With Diabetes |  | 1.754(1.334,2.307) | 0.000 |  |  |
| Urea, mmol/L |  | 1.011(0.984,1.039) | 0.422 |  |  |
| Dataset 3 |  |  |  |  |  |
| hb_group | Lower and decreasing | Ref |  | <0.000 | 2877.505 |
|  | Lower and growing slightly | 0.545(0.407,0.732) | 0.000 |  |  |
|  | Higher and growing slightly | 0.268(0.188,0.384) | 0.000 |  |  |
|  | Higher and growing steadily | 0.144(0.075,0.276) | 0.000 |  |  |
| eGFR, ml/min/1.73 m^2^ |  | 0.951(0.939,0.962) | <0.000 |  |  |
| Age, year |  | 0.979(0.971,0.988) | 0.000 |  |  |
| With Hypertension |  | 1.488(1.073,2.064) | 0.017 |  |  |
| With Diabetes |  | 1.754(1.334,2.307) | 0.000 |  |  |
| Urea, mmol/L |  | 1.012(0.985,1.040) | 0.377 |  |  |
| Dataset 4 |  |  |  |  |  |
| hb_group | Lower and decreasing | Ref |  |  | 2877.701 |
|  | Lower and growing slightly | 0.545(0.406,0.731) | 0.000 | <0.000 |  |
|  | Higher and growing slightly | 0.268(0.187,0.383) | 0.000 |  |  |
|  | Higher and growing steadily | 0.143(0.074,0.276) | 0.000 |  |  |
| eGFR, ml/min/1.73 m^2^ |  | 0.950(0.939,0.962) | <0.000 |  |  |
| Age, year |  | 0.979(0.971,0.988) | 0.000 |  |  |
| With Hypertension |  | 1.488(1.073,2.064) | 0.017 |  |  |
| With Diabetes |  | 1.752(1.332,2.304) | 0.000 |  |  |
| Urea, mmol/L |  | 1.011(0.983,1.039) | 0.452 |  |  |
| Dataset 5 |  |  |  |  |  |
| hb_group | Lower and decreasing | Ref |  | <0.000 | 2877.541 |
|  | Lower and growing slightly | 0.545(0.407,0.732) | 0.000 |  |  |
|  | Higher and growing slightly | 0.268(0.187,0.383) | 0.000 |  |  |
|  | Higher and growing steadily | 0.143(0.075,0.276) | 0.000 |  |  |
| eGFR, ml/min/1.73 m^2^ |  | 0.95(0.939,0.962) | <0.000 |  |  |
| Age, year |  | 0.979(0.971,0.988) | 0.000 |  |  |
| With Hypertension |  | 1.485(1.070,2.060) | 0.018 |  |  |
| With Diabetes |  | 1.751(1.332,2.303) | 0.000 |  |  |
| Urea, mmol/L |  | 1.012(0.985,1.039) | 0.390 |  |  |

Table 5 Multivariate Cox regression of trajectories of HCT

|  |  | HR(95%CI) | *P* | *P* for trend | AIC |
| --- | --- | --- | --- | --- | --- |
| Dataset 1 |  |  |  |  |  |
| hct_group | Lower and decreasing | Ref |  | <0.000 | 2858.577 |
|  | Lower and growing slightly | 0.568(0.429,0.753) | 0.000 |  |  |
|  | Higher and growing slightly | 0.217(0.147,0.319) | 0.000 |  |  |
|  | Higher and growing steadily | 0.114(0.052,0.249) | 0.000 |  |  |
| eGFR, ml/min/1.73 m^2^ |  | 0.951(0.940,0.963) | 0.000 |  |  |
| Age, year |  | 0.980(0.971,0.989) | 0.000 |  |  |
| With Hypertension |  | 1.469(1.056,2.042) | 0.022 |  |  |
| With Diabetes |  | 1.640(1.242,2.167) | 0.000 |  |  |
| Urea, mmol/L |  | 1.009(0.982,1.038) | 0.510 |  |  |
| Dataset 2 |  |  |  |  |  |
| hct_group | Lower and decreasing | Ref |  | <0.000 | 2856.990 |
|  | Lower and growing slightly | 0.564(0.426,0.747) | 0.000 |  |  |
|  | Higher and growing slightly | 0.214(0.146,0.314) | 0.000 |  |  |
|  | Higher and growing steadily | 0.112(0.051,0.246) | 0.000 |  |  |
| eGFR, ml/min/1.73 m^2^ |  | 0.950(0.939,0.961) | <0.000 |  |  |
| Age, year |  | 0.980(0.971,0.989) | 0.000 |  |  |
| With Hypertension |  | 1.476(1.062,2.051) | 0.020 |  |  |
| With Diabetes |  | 1.65(1.250,2.178) | 0.000 |  |  |
| Dataset 3 |  |  |  |  |  |
| hct_group | Lower and decreasing | Ref |  | <0.000 | 2856.990 |
|  | Lower and growing slightly | 0.564(0.426,0.747) | 0.000 |  |  |
|  | Higher and growing slightly | 0.214(0.146,0.314) | 0.000 |  |  |
|  | Higher and growing steadily | 0.112(0.051,0.246) | 0.000 |  |  |
| eGFR, ml/min/1.73 m^2^ |  | 0.950(0.939,0.961) | <0.000 |  |  |
| Age, year |  | 0.980(0.971,0.989) | 0.000 |  |  |
| With Hypertension |  | 1.476(1.062,2.051) | 0.020 |  |  |
| With Diabetes |  | 1.650(1.250,2.178) | 0.000 |  |  |
| Dataset 4 |  |  |  |  |  |
| hct_group | Lower and decreasing | Ref |  | <0.000 | 2858.628 |
|  | Lower and growing slightly | 0.568(0.429,0.752) | 0.000 |  |  |
|  | Higher and growing slightly | 0.216(0.147,0.319) | 0.000 |  |  |
|  | Higher and growing steadily | 0.113(0.052,0.248) | 0.000 |  |  |
| eGFR, ml/min/1.73 m^2^ |  | 0.951(0.940,0.963) | 0.000 |  |  |
| Age, year |  | 0.980(0.971,0.989) | 0.000 |  |  |
| With Hypertension |  | 1.470(1.057,2.044) | 0.022 |  |  |
| With Diabetes |  | 1.639(1.241,2.166) | 0.001 |  |  |
| Urea, mmol/L |  | 1.009(0.981,1.037) | 0.538 |  |  |
| Dataset 5 |  |  |  |  |  |
| hct_group | Lower and decreasing | Ref |  | <0.000 | 2858.476 |
|  | Lower and growing slightly | 0.568(0.429,0.753) | 0.000 |  |  |
|  | Higher and growing slightly | 0.217(0.147,0.319) | 0.000 |  |  |
|  | Higher and growing steadily | 0.114(0.052,0.249) | 0.000 |  |  |
| eGFR, ml/min/1.73 m^2^ |  | 0.952(0.940,0.963) | 0.000 |  |  |
| Age, year |  | 0.980(0.971,0.989) | 0.000 |  |  |
| With Hypertension |  | 1.468(1.056,2.041) | 0.023 |  |  |
| With Diabetes |  | 1.638(1.240,2.164) | 0.001 |  |  |
| Urea, mmol/L |  | 1.010(0.983,1.038) | 0.461 |  |  |

Table 6 Pooled results of trajectories of Hb

|  |  | HR(95%CI) | *P* | *P* for trend |
| --- | --- | --- | --- | --- |
| hb_group | Lower and decreasing | Ref |  |  |
|  | Lower and growing slightly | 0.545(0.406,0.731) | 0.000 | <0.000 |
|  | Higher and growing slightly | 0.268(0.187,0.383) | 0.000 |  |
|  | Higher and growing steadily | 0.143(0.075,0.276) | 0.000 |  |

Table 7 Pooled results of trajectories of HCT

|  |  | HR(95%CI) | *P* | *P* for trend |
| --- | --- | --- | --- | --- |
| hct_group | Lower and decreasing | Ref |  |  |
|  | Lower and growing slightly | 0.566(0.428,0.75) | 0.000 | <0.000 |
|  | Higher and growing slightly | 0.216(0.147,0.317) | 0.000 |  |
|  | Higher and growing steadily | 0.113(0.052,0.248) | 0.000 |  |

Supplementary file 4.5 Baseline characteristics of the Group 1 and Group 4

Table 8 Baseline characteristics of the Group 1 and Group 4

|  |  | Group 1 (“lower and decreasing” , Hb<100 g/L, HCT approximately 30%)  (N=129) | Group 4 ("higher and growing steadily" , Hb 145-160 g/L, HCT approximately 45%)  (N=113) | *P* |
| --- | --- | --- | --- | --- |
| Variables |  | Mean (SD)/Median (IQR)/N(%) | |  |
| baseline_Hb, g/L |  | 98.00 (90.00, 107.00) | 150.00 (140.00, 157.00) | <0.001 |
| mean_Hb, g/L |  | 95.29 (87.33, 101.00) | 148.22 (142.29, 155.67) | <0.001 |
| baseline_HCT, % |  | 30.30 (27.50, 32.70) | 44.40 (42.30, 46.80) | <0.001 |
| mean_HCT, % |  | 29.09 (26.77, 31.08) | 44.50 (42.82, 46.26) | <0.001 |
| Age, year |  | 60.44 (50.67, 70.62) | 57.13 (44.52, 65.65) | 0.009 |
| Sex | Female | 79 (61.24) | 5 (4.42) | <0.001 |
|  | Male | 50 (38.76) | 108 (95.58) |  |
| eGFR, ml/min/1.73 m2 |  | 28.57 (22.09, 44.34) | 47.72 (39.28, 55.43) | <0.001 |
| ALB, g/L |  | 37.40 (31.55, 41.20) | 44.80 (41.00, 46.95) | <0.001 |
| Urea, mmol/L |  | 11.01 (8.18, 14.16) | 7.45 (6.34, 8.89) | <0.001 |
| UA, mmol/L |  | 468.58 (123.35) | 474.09 (105.73) | 0.723 |
| TCO2, mmol/L |  | 22.79 (3.50) | 24.23 (2.49) | 0.001 |
| LDL-C, mmol/L |  | 3.08 (2.25, 4.05) | 3.21 (2.46, 3.99) | 0.531 |
| TC, mmol/L |  | 4.83 (3.81, 6.21) | 4.86 (4.34, 5.68) | 0.848 |
| HDL-C, mmol/L |  | 1.23 (0.93, 1.52) | 1.12 (0.94, 1.38) | 0.167 |
| AST, mmol/L |  | 18.00 (15.00, 22.00) | 20.00 (17.00, 25.00) | 0.012 |
| ALT, mmol/L |  | 13.00 (9.00, 17.00) | 20.00 (14.00, 28.00) | <0.001 |
| Protopathy | Primary Glomerulonephritides | 31 (24.03) | 36 (31.86) | 0.005 |
|  | Hypertensive Renal Disease | 2 (1.55) | 5 (4.42) |  |
|  | Diabetic nephropathy | 11 (8.53) | 1 (0.88) |  |
|  | Others | 6 (4.65) | 13 (11.50) |  |
|  | Unknown | 79 (61.24) | 58 (51.33) |  |
| With Hypertension | No | 23 (17.83) | 40 (35.40) | 0.003 |
|  | Yes | 106 (82.17) | 73 (64.60) |  |
| With Cardiovascular Disease | No | 101 (78.29) | 98 (86.73) | 0.123 |
|  | Yes | 28 (21.71) | 15 (13.27) |  |
| With Diabetes | No | 66 (51.16) | 93 (82.30) | <0.001 |
|  | Yes | 63 (48.84) | 20 (17.70) |  |
| With Hyperuricemia | No | 120 (93.02) | 99 (87.61) | 0.225 |
|  | Yes | 9 (6.98) | 14 (12.39) |  |
| With Hyperlipidemia | No | 102 (79.07) | 96 (84.96) | 0.309 |
|  | Yes | 27 (20.93) | 17 (15.04) |  |
| With Anemia | No | 114 (88.37) | 112 (99.12) | 0.002 |
|  | Yes | 15 (11.63) | 1 (0.88) |  |
| With ACEI/ARB | No | 75 (58.14) | 68 (60.18) | 0.849 |
|  | Yes | 54 (41.86) | 45 (39.82) |  |
| With Calcium Supplements | No | 84 (65.12) | 92 (81.42) | 0.007 |
|  | Yes | 45 (34.88) | 21 (18.58) |  |
| With Sodium Bicarbonate | No | 76 (58.91) | 67 (59.29) | 1.000 |
|  | Yes | 53 (41.09) | 46 (40.71) |  |
| With Ketoacid Tablets | No | 80 (62.02) | 82 (72.57) | 0.109 |
|  | Yes | 49 (37.98) | 31 (27.43) |  |
| With Diuretics | No | 81 (62.79) | 100 (88.50) | <0.001 |
|  | Yes | 48 (37.21) | 13 (11.50) |  |
| With ESAs or Iron | No | 76(58.91) | 107(94.69) | <0.001 |
|  | Yes | 53(41.09) | 6(5.31) |  |
| Follow-up duration |  | 17.44 (11.08, 31.74) | 47.28 (28.75, 62.26) | <0.001 |
| Composite outcomes (%) | No | 46 (35.66) | 102 (90.27) | <0.001 |
|  | Yes | 83 (64.34) | 11 (9.73) |  |

Note: estimated glomerular filtration rate, eGFR; albumin, ALB; uric acid, UA; total carbon dioxide, TCO2; low-density lipoprotein cholesterol, LDL-C; total cholesterol, TC; high-density lipoprotein cholesterol, HDL-C; aspartate transaminase, AST; alanine aminotransferase, ALT; angiotensin converting enzyme inhibitors, ACEI; angiotensin receptor blocker, ARB;Primary Glomerulonephritides included chronic nephritis, nephropathy syndrome and IgA nephropathy.Other secondary nephrosis included systemic lupus erythematosus nephritis, Henoch-Schonlein purpura,Hepatitis B virus-associated nephritis and obstructive nephropathy, etc.; hemoglobin, Hb; hematocrit, HCT.
