# Supplementary material for: Time-updated patterns of hemoglobin and hematocrit and the risk of CKD progression
Source: Front Endocrinol (Lausanne). 2025 Oct 30;16:1642307. doi: 10.3389/fendo.2025.1642307 (PMC12611651; doi:10.3389/fendo.2025.1642307)
Supplement: Supplementary file 5 [file DataSheet5.docx]

**Supplementary file 5**

|  |  | Dataset 1 | | | Dataset 2 | | | Dataset 3 | | | Dataset 4 | | | Dataset 5 | | | Pooled results | | |
| --- | --- | --- | --- | --- | --- | --- | --- | --- | --- | --- | --- | --- | --- | --- | --- | --- | --- | --- | --- |
|  |  | HR(95%CI) | *P* | *P* for interaction | HR(95%CI) | *P* | *P* for interaction | HR(95%CI) | *P* | *P* for interaction | HR(95%CI) | *P* | *P* for interaction | HR(95%CI) | *P* | *P* for interaction | HR(95%CI) | *P* | *P* for interaction |
| Age<59.3 year old |  |  |  |  |  |  |  |  |  |  |  |  |  |  |  |  |  |  |  |
| mean_hb |  | 0.829(0.743,0.926) | 0.001 | 0.555 | 0.829(0.742,0.925) | 0.001 | 0.557 | 0.829(0.743,0.926) | 0.001 | 0.553 | 0.828(0.743,0.923) | 0.001 | 0.556 | 0.829(0.742,0.925) | 0.001 | 0.552 | 0.829(0.742,0.925) | 0.001 | 0.556 |
| mean_hct |  | 0.522(0.360,0.756) | 0.001 | 0.458 | 0.522(0.360,0.756) | 0.001 | 0.458 | 0.522(0.357,0.763) | 0.001 | 0.459 | 0.522(0.360,0.756) | 0.001 | 0.458 | 0.522(0.360,0.756) | 0.001 | 0.458 | 0.522(0.360,0.757) | 0.001 | 0.458 |
| hb_group | Lower and decreasing | Ref |  |  | Ref |  |  | Ref |  |  | Ref |  |  | Ref |  |  | Ref |  |  |
|  | Lower and growing slightly | 0.654(0.431,0.994) | 0.046 | 0.369 | 0.652(0.430,0.991) | 0.045 | 0.374 | 0.656(0.432,0.995) | 0.048 | 0.361 | 0.649(0.428,0.983) | 0.041 | 0.375 | 0.652(0.430,0.991) | 0.045 | 0.366 | 0.652(0.430,0.991) | 0.045 | 0.368 |
|  | Higher and growing slightly | 0.270(0.164,0.445) | 0.000 | 0.758 | 0.270(0.164,0.444) | 0.000 | 0.754 | 0.271(0.164,0.447) | 0.000 | 0.763 | 0.268(0.163,0.442) | 0.000 | 0.759 | 0.269(0.163,0.443) | 0.000 | 0.763 | 0.270(0.164,0.444) | 0.000 | 0.759 |
|  | Higher and growing steadily | 0.185(0.087,0.398) | 0.000 | 0.450 | 0.185(0.086,0.396) | 0.000 | 0.451 | 0.186(0.087,0.398) | 0.000 | 0.448 | 0.184(0.086,0.394) | 0.000 | 0.451 | 0.185(0.086,0.396) | 0.000 | 0.449 | 0.185(0.086,0.396) | 0.000 | 0.450 |
| hct_group | Lower and decreasing | Ref |  |  | Ref |  |  | Ref |  |  | Ref |  |  | Ref |  |  | Ref |  |  |
|  | Lower and growing slightly | 0.598(0.405,0.882) | 0.010 | 0.881 | 0.591(0.401,0.871) | 0.008 | 0.872 | 0.591(0.401,0.871) | 0.008 | 0.881 | 0.595(0.403,0.877) | 0.009 | 0.880 | 0.596(0.404,0.879) | 0.009 | 0.885 | 0.594(0.403,0.876) | 0.009 | 0.928 |
|  | Higher and growing slightly | 0.199(0.119,0.333) | 0.000 | 0.494 | 0.195(0.117,0.326) | 0.000 | 0.494 | 0.195(0.117,0.326) | 0.000 | 0.681 | 0.198(0.119,0.331) | 0.000 | 0.497 | 0.199(0.119,0.332) | 0.000 | 0.495 | 0.197(0.118,0.330) | 0.000 | 0.534 |
|  | Higher and growing steadily | 0.157(0.066,0.376) | 0.000 | 0.416 | 0.154(0.065,0.368) | 0.000 | 0.419 | 0.154(0.065,0.368) | 0.000 | 0.360 | 0.156(0.065,0.373) | 0.000 | 0.416 | 0.157(0.066,0.375) | 0.000 | 0.417 | 0.156(0.065,0.372) | 0.000 | 0.406 |
| Age≥59.3 year old |  |  |  |  |  |  |  |  |  |  |  |  |  |  |  |  |  |  |  |
| mean_hb |  | 0.872(0.774,0.983) | 0.026 |  | 0.872(0.774,0.983) | 0.026 |  | 0.872(0.774,0.983) | 0.026 |  | 0.873(0.774,0.984) | 0.026 |  | 0.873(0.774,0.984) | 0.027 |  | 0.872(0.774,0.983) | 0.026 |  |
| mean_hct |  | 0.631(0.420,0.947) | 0.026 |  | 0.631(0.420,0.947) | 0.026 |  | 0.632(0.421,0.949) | 0.027 |  | 0.631(0.420,0.947) | 0.026 |  | 0.631(0.420,0.947) | 0.026 |  | 0.631(0.420,0.947) | 0.026 |  |
| hb_group | Lower and decreasing | Ref |  |  | Ref |  |  | Ref |  |  | Ref |  |  | Ref |  |  | Ref |  |  |
|  | Lower and growing slightly | 0.476(0.309,0.734) | 0.001 |  | 0.477(0.309,0.735) | 0.001 |  | 0.474(0.307,0.731) | 0.001 |  | 0.473(0.307,0.73) | 0.001 |  | 0.474(0.307,0.731) | 0.001 |  | 0.475(0.308,0.732) | 0.001 |  |
|  | Higher and growing slightly | 0.271(0.162,0.454) | 0.000 |  | 0.272(0.162,0.455) | 0.000 |  | 0.271(0.162,0.454) | 0.000 |  | 0.27(0.161,0.453) | 0.000 |  | 0.270(0.161,0.452) | 0.000 |  | 0.271(0.162,0.453) | 0.000 |  |
|  | Higher and growing steadily | 0.075(0.018,0.320) | 0.000 |  | 0.075(0.018,0.319) | 0.000 |  | 0.075(0.018,0.319) | 0.000 |  | 0.075(0.018,0.317) | 0.000 |  | 0.074(0.018,0.316) | 0.000 |  | 0.075(0.018,0.318) | 0.000 |  |
| hct_group | Lower and decreasing | Ref |  |  | Ref |  |  | Ref |  |  | Ref |  |  | Ref |  |  | Ref |  |  |
|  | Lower and growing slightly | 0.517(0.344,0.776) | 0.001 |  | 0.510(0.340,0.766) | 0.001 |  | 0.510(0.340,0.766) | 0.001 |  | 0.518(0.344,0.778) | 0.002 |  | 0.518(0.345,0.779) | 0.002 |  | 0.515(0.343,0.773) | 0.001 |  |
|  | Higher and growing slightly | 0.216(0.120,0.39) | 0.000 |  | 0.212(0.118,0.382) | 0.000 |  | 0.212(0.118,0.382) | 0.000 |  | 0.216(0.12,0.391) | 0.000 |  | 0.216(0.12,0.391) | 0.000 |  | 0.214(0.119,0.387) | 0.000 |  |
|  | Higher and growing steadily | 0.043(0.006,0.316) | 0.002 |  | 0.043(0.006,0.318) | 0.002 |  | 0.043(0.006,0.318) | 0.002 |  | 0.043(0.006,0.314) | 0.002 |  | 0.043(0.006,0.314) | 0.002 |  | 0.043(0.006,0.316) | 0.002 |  |
| Female |  |  |  |  |  |  |  |  |  |  |  |  |  |  |  |  |  |  |  |
| mean_hb |  | 0.794(0.683,0.923) | 0.003 | 0.034 | 0.795(0.683,0.924) | 0.003 | 0.034 | 0.795(0.683,0.924) | 0.003 | 0.034 | 0.795(0.683,0.924) | 0.003 | 0.034 | 0.794(0.683,0.923) | 0.003 | 0.034 | 0.794(0.683,0.923) | 0.003 | 0.034 |
| mean_hct |  | 0.480(0.293,0.786) | 0.004 | 0.092 | 0.480(0.293,0.786) | 0.004 | 0.092 | 0.478(0.293,0.782) | 0.003 | 0.096 | 0.480(0.293,0.786) | 0.004 | 0.092 | 0.480(0.293,0.786) | 0.004 | 0.092 | 0.479(0.293,0.785) | 0.004 | 0.092 |
| hb_group | Lower and decreasing | Ref |  |  | Ref |  |  | Ref |  |  | Ref |  |  | Ref |  |  | Ref |  |  |
|  | Lower and growing slightly | 0.514(0.345,0.765) | 0.001 | 0.821 | 0.514(0.345,0.766) | 0.001 | 0.827 | 0.515(0.346,0.766) | 0.001 | 0.820 | 0.515(0.345,0.769) | 0.001 | 0.829 | 0.513(0.345,0.764) | 0.001 | 0.818 | 0.514(0.345,0.766) | 0.001 | 0.823 |
|  | Higher and growing slightly | 0.157(0.082,0.303) | 0.000 | 0.145 | 0.157(0.082,0.303) | 0.000 | 0.147 | 0.157(0.082,0.303) | 0.000 | 0.144 | 0.157(0.082,0.303) | 0.000 | 0.148 | 0.159(0.082,0.306) | 0.000 | 0.145 | 0.158(0.082,0.304) | 0.000 | 0.146 |
|  | Higher and growing steadily | 0.000(0.000,Inf) | 0.994 | 0.992 | 0.000(0.000,Inf) | 0.994 | 0.992 | 0.000(0.000,Inf) | 0.994 | 0.992 | 0.000(0.000,Inf) | 0.994 | 0.992 | 0.000(0.000,Inf) | 0.994 | 0.992 | 0.000(0.000,Inf) | 0.994 | 0.992 |
| hct_group | Lower and decreasing | Ref |  |  | Ref |  |  | Ref |  |  | Ref |  |  | Ref |  |  | Ref |  |  |
|  | Lower and growing slightly | 0.544(0.365,0.810) | 0.003 | 0.736 | 0.544(0.366,0.809) | 0.003 | 0.794 | 0.544(0.366,0.809) | 0.003 | 0.794 | 0.546(0.367,0.813) | 0.003 | 0.741 | 0.542(0.364,0.806) | 0.003 | 0.728 | 0.544(0.366,0.809) | 0.003 | 0.758 |
|  | Higher and growing slightly | 0.139(0.068,0.285) | 0.000 | 0.173 | 0.139(0.068,0.285) | 0.000 | 0.187 | 0.139(0.068,0.285) | 0.000 | 0.187 | 0.139(0.068,0.285) | 0.000 | 0.175 | 0.139(0.068,0.285) | 0.000 | 0.171 | 0.139(0.068,0.285) | 0.000 | 0.179 |
|  | Higher and growing steadily | 0.000(0.000,Inf) | 0.995 | 0.994 | 0.000(0.000,Inf) | 0.995 | 0.994 | 0.000(0.000,Inf) | 0.995 | 0.994 | 0.000(0.000,Inf) | 0.995 | 0.994 | 0.000(0.000,Inf) | 0.995 | 0.994 | 0.000(0.000,Inf) | 0.995 | 0.994 |
| Male |  |  |  |  |  |  |  |  |  |  |  |  |  |  |  |  |  |  |  |
| mean_hb |  | 0.880(0.799,0.969) | 0.009 |  | 0.880(0.799,0.969) | 0.009 |  | 0.881(0.800,0.970) | 0.010 |  | 0.881(0.800,0.970) | 0.010 |  | 0.880(0.799,0.969) | 0.009 |  | 0.880(0.799,0.969) | 0.009 |  |
| mean_hct |  | 0.641(0.461,0.892) | 0.008 |  | 0.641(0.461,0.892) | 0.008 |  | 0.649(0.464,0.908) | 0.012 |  | 0.641(0.461,0.892) | 0.008 |  | 0.641(0.461,0.892) | 0.008 |  | 0.643(0.462,0.895) | 0.009 |  |
| hb_group | Lower and decreasing | Ref |  |  | Ref |  |  | Ref |  |  | Ref |  |  | Ref |  |  | Ref |  |  |
|  | Lower and growing slightly | 0.553(0.349,0.874) | 0.011 |  | 0.552(0.349,0.873) | 0.011 |  | 0.554(0.350,0.876) | 0.011 |  | 0.553(0.350,0.875) | 0.012 |  | 0.551(0.348,0.873) | 0.011 |  | 0.553(0.349,0.874) | 0.011 |  |
|  | Higher and growing slightly | 0.293(0.179,0.479) | 0.000 |  | 0.293(0.179,0.479) | 0.000 |  | 0.294(0.180,0.481) | 0.000 |  | 0.294(0.180,0.480) | 0.000 |  | 0.292(0.178,0.477) | 0.000 |  | 0.293(0.179,0.479) | 0.000 |  |
|  | Higher and growing steadily | 0.123(0.058,0.262) | 0.000 |  | 0.123(0.058,0.26) | 0.000 |  | 0.124(0.059,0.262) | 0.000 |  | 0.124(0.058,0.262) | 0.000 |  | 0.123(0.058,0.260) | 0.000 |  | 0.123(0.058,0.261) | 0.000 |  |
| hct_group | Lower and decreasing | Ref |  |  | Ref |  |  | Ref |  |  | Ref |  |  | Ref |  |  | Ref |  |  |
|  | Lower and growing slightly | 0.586(0.388,0.884) | 0.011 |  | 0.572(0.382,0.855) | 0.006 |  | 0.572(0.382,0.855) | 0.006 |  | 0.585(0.389,0.886) | 0.011 |  | 0.585(0.388,0.882) | 0.010 |  | 0.580(0.386,0.873) | 0.009 |  |
|  | Higher and growing slightly | 0.246(0.149,0.408) | 0.000 |  | 0.239(0.146,0.391) | 0.000 |  | 0.239(0.146,0.391) | 0.000 |  | 0.246(0.149,0.409) | 0.000 |  | 0.245(0.148,0.406) | 0.000 |  | 0.243(0.147,0.401) | 0.000 |  |
|  | Higher and growing steadily | 0.100(0.043,0.232) | 0.000 |  | 0.098(0.042,0.225) | 0.000 |  | 0.098(0.042,0.225) | 0.000 |  | 0.100(0.043,0.233) | 0.000 |  | 0.100(0.043,0.232) | 0.000 |  | 0.099(0.043,0.230) | 0.000 |  |
| CKD stage 3 |  |  |  |  |  |  |  |  |  |  |  |  |  |  |  |  |  |  |  |
| mean_hb |  | 0.730(0.644,0.827) | 0.000 | 0.433 | 0.730(0.644,0.827) | 0.000 | 0.436 | 0.730(0.644,0.827) | 0.000 | 0.431 | 0.731(0.644,0.828) | 0.000 | 0.434 | 0.731(0.645,0.829) | 0.000 | 0.429 | 0.731(0.644,0.828) | 0.000 | 0.432 |
| mean_hct |  | 0.351(0.229,0.538) | 0.000 | 0.449 | 0.351(0.229,0.538) | 0.000 | 0.449 | 0.359(0.235,0.55) | 0.000 | 0.455 | 0.353(0.231,0.539) | 0.000 | 0.449 | 0.351(0.229,0.538) | 0.000 | 0.449 | 0.353(0.231,0.541) | 0.000 | 0.450 |
| hb_group | Lower and decreasing | Ref |  |  | Ref |  |  | Ref |  |  | Ref |  |  | Ref |  |  | Ref |  |  |
|  | Lower and growing slightly | 0.340(0.209,0.552) | 0.000 | 0.014 | 0.340(0.209,0.552) | 0.000 | 0.014 | 0.340(0.210,0.550) | 0.000 | 0.014 | 0.341(0.210,0.554) | 0.000 | 0.015 | 0.343(0.212,0.557) | 0.000 | 0.015 | 0.341(0.210,0.553) | 0.000 | 0.015 |
|  | Higher and growing slightly | 0.228(0.131,0.396) | 0.000 | 0.573 | 0.231(0.133,0.401) | 0.000 | 0.574 | 0.228(0.132,0.396) | 0.000 | 0.567 | 0.228(0.131,0.395) | 0.000 | 0.574 | 0.229(0.132,0.398) | 0.000 | 0.570 | 0.229(0.132,0.397) | 0.000 | 0.571 |
|  | Higher and growing steadily | 0.121(0.053,0.275) | 0.000 | 0.913 | 0.122(0.054,0.278) | 0.000 | 0.913 | 0.122(0.054,0.277) | 0.000 | 0.911 | 0.122(0.053,0.277) | 0.000 | 0.913 | 0.123(0.054,0.281) | 0.000 | 0.914 | 0.122(0.053,0.278) | 0.000 | 0.913 |
| hct_group | Lower and decreasing | Ref |  |  | Ref |  |  | Ref |  |  | Ref |  |  | Ref |  |  | Ref |  |  |
|  | Lower and growing slightly | 0.439(0.281,0.685) | 0.000 | 0.213 | 0.415(0.268,0.642) | 0.000 | 0.222 | 0.415(0.268,0.642) | 0.000 | 0.222 | 0.384(0.249,0.592) | 0.000 | 0.213 | 0.444(0.285,0.694) | 0.000 | 0.212 | 0.430(0.275,0.674) | 0.000 | 0.217 |
|  | Higher and growing slightly | 0.164(0.089,0.303) | 0.000 | 0.335 | 0.148(0.082,0.269) | 0.000 | 0.348 | 0.148(0.082,0.269) | 0.000 | 0.348 | 0.133(0.075,0.237) | 0.000 | 0.333 | 0.167(0.091,0.308) | 0.000 | 0.331 | 0.158(0.085,0.294) | 0.000 | 0.339 |
|  | Higher and growing steadily | 0.097(0.037,0.254) | 0.000 | 0.923 | 0.086(0.033,0.223) | 0.000 | 0.924 | 0.086(0.033,0.223) | 0.000 | 0.924 | 0.079(0.031,0.201) | 0.000 | 0.925 | 0.1(0.038,0.261) | 0.000 | 0.924 | 0.093(0.035,0.246) | 0.000 | 0.924 |
| CKD stage 4 |  |  |  |  |  |  |  |  |  |  |  |  |  |  |  |  |  |  |  |
| mean_hb |  | 0.970(0.861,1.094) | 0.626 |  | 0.969(0.860,1.093) | 0.615 |  | 0.970(0.861,1.094) | 0.621 |  | 0.969(0.860,1.093) | 0.617 |  | 0.970(0.861,1.094) | 0.618 |  | 0.970(0.861,1.094) | 0.619 |  |
| mean_hct |  | 0.884(0.586,1.334) | 0.556 |  | 0.884(0.586,1.334) | 0.556 |  | 0.883(0.582,1.337) | 0.556 |  | 0.902(0.598,1.360) | 0.622 |  | 0.884(0.586,1.334) | 0.556 |  | 0.887(0.587,1.340) | 0.569 |  |
| hb_group | Lower and decreasing | Ref |  |  | Ref |  |  | Ref |  |  | Ref |  |  | Ref |  |  | Ref |  |  |
|  | Lower and growing slightly | 0.733(0.508,1.058) | 0.098 |  | 0.733(0.508,1.057) | 0.096 |  | 0.733(0.508,1.057) | 0.097 |  | 0.733(0.508,1.057) | 0.097 |  | 0.733(0.508,1.058) | 0.097 |  | 0.733(0.508,1.057) | 0.097 |  |
|  | Higher and growing slightly | 0.277(0.170,0.452) | 0.000 |  | 0.276(0.169,0.450) | 0.000 |  | 0.276(0.169,0.451) | 0.000 |  | 0.276(0.169,0.450) | 0.000 |  | 0.276(0.169,0.451) | 0.000 |  | 0.276(0.169,0.451) | 0.000 |  |
|  | Higher and growing steadily | 0.141(0.043,0.465) | 0.001 |  | 0.140(0.042,0.464) | 0.001 |  | 0.141(0.043,0.465) | 0.001 |  | 0.140(0.042,0.464) | 0.001 |  | 0.141(0.043,0.464) | 0.001 |  | 0.141(0.043,0.465) | 0.001 |  |
| hct_group | Lower and decreasing | Ref |  |  | Ref |  |  | Ref |  |  | Ref |  |  | Ref |  |  | Ref |  |  |
|  | Lower and growing slightly | 0.664(0.462,0.955) | 0.027 |  | 0.655(0.457,0.938) | 0.021 |  | 0.655(0.457,0.938) | 0.021 |  | 0.596(0.418,0.849) | 0.025 |  | 0.663(0.461,0.952) | 0.026 |  | 0.660(0.459,0.947) | 0.024 |  |
|  | Higher and growing slightly | 0.272(0.163,0.452) | 0.000 |  | 0.267(0.161,0.443) | 0.000 |  | 0.267(0.161,0.443) | 0.000 |  | 0.244(0.146,0.406) | 0.000 |  | 0.271(0.163,0.451) | 0.000 |  | 0.270(0.162,0.448) | 0.000 |  |
|  | Higher and growing steadily | 0.100(0.024,0.417) | 0.002 |  | 0.099(0.024,0.415) | 0.002 |  | 0.099(0.024,0.415) | 0.002 |  | 0.101(0.025,0.416) | 0.002 |  | 0.100(0.024,0.417) | 0.002 |  | 0.100(0.024,0.416) | 0.002 |  |
| With Hypertension |  |  |  |  |  |  |  |  |  |  |  |  |  |  |  |  |  |  |  |
| mean_hb |  | 0.851(0.779,0.930) | 0.000 | 0.938 | 0.851(0.779,0.930) | 0.000 | 0.936 | 0.852(0.780,0.931) | 0.000 | 0.941 | 0.852(0.780,0.931) | 0.000 | 0.937 | 0.852(0.780,0.931) | 0.000 | 0.943 | 0.852(0.780,0.931) | 0.000 | 0.939 |
| mean_hct |  | 0.582(0.432,0.784) | 0.000 | 0.960 | 0.582(0.432,0.784) | 0.000 | 0.960 | 0.577(0.426,0.780) | 0.000 | 0.953 | 0.582(0.432,0.784) | 0.000 | 0.960 | 0.582(0.432,0.784) | 0.000 | 0.960 | 0.581(0.431,0.783) | 0.000 | 0.958 |
| hb_group | Lower and decreasing | Ref |  |  | Ref |  |  | Ref |  |  | Ref |  |  | Ref |  |  | Ref |  |  |
|  | Lower and growing slightly | 0.568(0.413,0.782) | 0.001 | 0.547 | 0.567(0.412,0.781) | 0.001 | 0.550 | 0.568(0.413,0.782) | 0.001 | 0.549 | 0.567(0.412,0.781) | 0.001 | 0.553 | 0.568(0.413,0.782) | 0.001 | 0.542 | 0.568(0.413,0.782) | 0.001 | 0.548 |
|  | Higher and growing slightly | 0.275(0.185,0.410) | 0.000 | 0.672 | 0.275(0.185,0.410) | 0.000 | 0.677 | 0.276(0.185,0.410) | 0.000 | 0.679 | 0.275(0.185,0.410) | 0.000 | 0.679 | 0.275(0.185,0.410) | 0.000 | 0.667 | 0.275(0.185,0.410) | 0.000 | 0.675 |
|  | Higher and growing steadily | 0.144(0.064,0.321) | 0.000 | 0.883 | 0.144(0.064,0.321) | 0.000 | 0.885 | 0.144(0.064,0.322) | 0.000 | 0.887 | 0.144(0.064,0.321) | 0.000 | 0.888 | 0.144(0.064,0.321) | 0.000 | 0.880 | 0.144(0.064,0.321) | 0.000 | 0.885 |
| hct_group | Lower and decreasing | Ref |  |  | Ref |  |  | Ref |  |  | Ref |  |  | Ref |  |  | Ref |  |  |
|  | Lower and growing slightly | 0.572(0.420,0.780) | 0.000 | 0.693 | 0.568(0.418,0.774) | 0.000 | 0.716 | 0.568(0.418,0.774) | 0.000 | 0.716 | 0.529(0.390,0.719) | 0.000 | 0.701 | 0.573(0.420,0.780) | 0.000 | 0.689 | 0.571(0.419,0.777) | 0.000 | 0.703 |
|  | Higher and growing slightly | 0.230(0.151,0.35) | 0.000 | 0.444 | 0.227(0.149,0.345) | 0.000 | 0.454 | 0.227(0.149,0.345) | 0.000 | 0.454 | 0.212(0.140,0.321) | 0.000 | 0.447 | 0.230(0.151,0.350) | 0.000 | 0.442 | 0.229(0.150,0.348) | 0.000 | 0.448 |
|  | Higher and growing steadily | 0.108(0.039,0.300) | 0.000 | 0.989 | 0.107(0.039,0.297) | 0.000 | 0.999 | 0.107(0.039,0.297) | 0.000 | 0.999 | 0.092(0.033,0.254) | 0.000 | 0.992 | 0.108(0.039,0.301) | 0.000 | 0.987 | 0.108(0.039,0.299) | 0.000 | 0.993 |
| Without Hypertension |  |  |  |  |  |  |  |  |  |  |  |  |  |  |  |  |  |  |  |
| mean_hb |  | 0.849(0.691,1.043) | 0.116 |  | 0.849(0.691,1.043) | 0.116 |  | 0.848(0.690,1.042) | 0.115 |  | 0.848(0.692,1.040) | 0.113 |  | 0.849(0.691,1.043) | 0.116 |  | 0.848(0.690,1.042) | 0.115 |  |
| mean_hct |  | 0.582(0.432,0.784) | 0.000 |  | 0.570(0.283,1.148) | 0.115 |  | 0.580(0.286,1.175) | 0.130 |  | 0.570(0.283,1.148) | 0.115 |  | 0.570(0.283,1.148) | 0.115 |  | 0.574(0.302,1.091) | 0.090 |  |
| hb_group | Lower and decreasing | Ref |  |  | Ref |  |  | Ref |  |  | Ref |  |  | Ref |  |  | Ref |  |  |
|  | Lower and growing slightly | 0.482(0.222,1.05) | 0.066 |  | 0.483(0.222,1.051) | 0.067 |  | 0.482(0.221,1.049) | 0.066 |  | 0.480(0.22,1.048) | 0.065 |  | 0.480(0.221,1.044) | 0.064 |  | 0.481(0.221,1.048) | 0.066 |  |
|  | Higher and growing slightly | 0.240(0.103,0.561) | 0.001 |  | 0.241(0.103,0.565) | 0.001 |  | 0.242(0.103,0.567) | 0.001 |  | 0.240(0.102,0.561) | 0.001 |  | 0.239(0.102,0.559) | 0.001 |  | 0.240(0.103,0.563) | 0.001 |  |
|  | Higher and growing steadily | 0.127(0.039,0.414) | 0.001 |  | 0.127(0.039,0.414) | 0.001 |  | 0.128(0.039,0.415) | 0.001 |  | 0.127(0.039,0.414) | 0.001 |  | 0.127(0.039,0.412) | 0.001 |  | 0.127(0.039,0.414) | 0.001 |  |
| hct_group | Lower and decreasing | Ref |  |  | Ref |  |  | Ref |  |  | Ref |  |  | Ref |  |  | Ref |  |  |
|  | Lower and growing slightly | 0.532(0.272,1.041) | 0.066 |  | 0.534(0.273,1.044) | 0.067 |  | 0.534(0.273,1.044) | 0.067 |  | 0.483(0.250,0.933) | 0.067 |  | 0.53(0.271,1.038) | 0.064 |  | 0.533(0.272,1.043) | 0.066 |  |
|  | Higher and growing slightly | 0.163(0.061,0.439) | 0.000 |  | 0.161(0.060,0.431) | 0.000 |  | 0.161(0.060,0.431) | 0.000 |  | 0.154(0.058,0.406) | 0.000 |  | 0.162(0.060,0.437) | 0.000 |  | 0.162(0.060,0.435) | 0.000 |  |
|  | Higher and growing steadily | 0.100(0.028,0.361) | 0.000 |  | 0.101(0.028,0.364) | 0.000 |  | 0.101(0.028,0.364) | 0.000 |  | 0.134(0.038,0.472) | 0.000 |  | 0.100(0.028,0.360) | 0.000 |  | 0.101(0.028,0.363) | 0.000 |  |
| With Diabetes |  |  |  |  |  |  |  |  |  |  |  |  |  |  |  |  |  |  |  |
| mean_hb |  | 0.862(0.755,0.985) | 0.030 | 0.096 | 0.863(0.756,0.986) | 0.031 | 0.096 | 0.864(0.756,0.987) | 0.032 | 0.097 | 0.863(0.756,0.986) | 0.031 | 0.096 | 0.864(0.756,0.987) | 0.032 | 0.098 | 0.863(0.756,0.986) | 0.031 | 0.097 |
| mean_hct |  | 0.62(0.394,0.976) | 0.039 | 0.000 | 0.62(0.394,0.976) | 0.039 | 0.153 | 0.631(0.401,0.992) | 0.046 | 0.147 | 0.62(0.394,0.976) | 0.039 | 0.153 | 0.62(0.394,0.976) | 0.039 | 0.153 | 0.622(0.395,0.979) | 0.040 | 0.776 |
| hb_group | Lower and decreasing | Ref |  |  | Ref |  |  | Ref |  |  | Ref |  |  | Ref |  |  | Ref |  |  |
|  | Lower and growing slightly | 0.488(0.310,0.769) | 0.002 | 0.698 | 0.484(0.307,0.762) | 0.002 | 0.702 | 0.482(0.306,0.759) | 0.002 | 0.693 | 0.484(0.307,0.763) | 0.002 | 0.699 | 0.486(0.309,0.764) | 0.002 | 0.698 | 0.485(0.308,0.764) | 0.002 | 0.698 |
|  | Higher and growing slightly | 0.407(0.243,0.682) | 0.001 | 0.061 | 0.409(0.244,0.684) | 0.001 | 0.062 | 0.407(0.243,0.682) | 0.001 | 0.063 | 0.406(0.243,0.680) | 0.001 | 0.062 | 0.407(0.243,0.682) | 0.001 | 0.062 | 0.407(0.243,0.682) | 0.001 | 0.062 |
|  | Higher and growing steadily | 0.133(0.031,0.567) | 0.006 | 0.949 | 0.133(0.031,0.568) | 0.006 | 0.949 | 0.133(0.031,0.568) | 0.006 | 0.946 | 0.133(0.031,0.568) | 0.006 | 0.950 | 0.133(0.031,0.570) | 0.007 | 0.950 | 0.133(0.031,0.569) | 0.006 | 0.949 |
| hct_group | Lower and decreasing | Ref |  |  | Ref |  |  | Ref |  |  | Ref |  |  | Ref |  |  | Ref |  |  |
|  | Lower and growing slightly | 0.486(0.312,0.757) | 0.001 | 0.392 | 0.479(0.308,0.744) | 0.001 | 0.390 | 0.479(0.308,0.744) | 0.001 | 0.390 | 0.467(0.301,0.726) | 0.001 | 0.388 | 0.488(0.313,0.760) | 0.001 | 0.393 | 0.482(0.310,0.751) | 0.001 | 0.391 |
|  | Higher and growing slightly | 0.333(0.189,0.587) | 0.000 | 0.059 | 0.323(0.184,0.567) | 0.000 | 0.059 | 0.323(0.184,0.567) | 0.000 | 0.059 | 0.364(0.209,0.635) | 0.000 | 0.059 | 0.333(0.189,0.587) | 0.000 | 0.059 | 0.328(0.186,0.577) | 0.000 | 0.059 |
|  | Higher and growing steadily | 0.000(0.000,Inf) | 0.993 | 0.992 | 0.000(0.000,Inf) | 0.993 | 0.992 | 0.000(0.000,Inf) | 0.993 | 0.992 | 0.000(0.000,Inf) | 0.995 | 0.992 | 0.000(0.000,Inf) | 0.993 | 0.992 | 0.000(0.000,Inf) | 0.993 | 0.992 |
| Without Diabetes |  |  |  |  |  |  |  |  |  |  |  |  |  |  |  |  |  |  |  |
| mean_hb |  | 0.832(0.75,0.923) | 0.001 |  | 0.830(0.748,0.921) | 0.000 |  | 0.830(0.748,0.921) | 0.000 |  | 0.831(0.749,0.922) | 0.000 |  | 0.831(0.749,0.922) | 0.000 |  | 0.831(0.749,0.922) | 0.000 |  |
| mean_hct |  | 0.541(0.380,0.771) | 0.001 |  | 0.541(0.380,0.771) | 0.001 |  | 0.525(0.365,0.753) | 0.000 |  | 0.541(0.380,0.771) | 0.001 |  | 0.541(0.380,0.771) | 0.001 |  | 0.538(0.377,0.768) | 0.001 |  |
| hb_group | Lower and decreasing | Ref |  |  | Ref |  |  | Ref |  |  | Ref |  |  | Ref |  |  | Ref |  |  |
|  | Lower and growing slightly | 0.554(0.374,0.822) | 0.003 |  | 0.550(0.371,0.815) | 0.003 |  | 0.550(0.371,0.815) | 0.003 |  | 0.552(0.372,0.819) | 0.003 |  | 0.552(0.372,0.819) | 0.003 |  | 0.552(0.372,0.818) | 0.003 |  |
|  | Higher and growing slightly | 0.197(0.121,0.323) | 0.000 |  | 0.196(0.120,0.320) | 0.000 |  | 0.196(0.120,0.320) | 0.000 |  | 0.197(0.120,0.321) | 0.000 |  | 0.197(0.120,0.321) | 0.000 |  | 0.196(0.120,0.321) | 0.000 |  |
|  | Higher and growing steadily | 0.142(0.067,0.302) | 0.000 |  | 0.141(0.066,0.300) | 0.000 |  | 0.141(0.066,0.300) | 0.000 |  | 0.142(0.067,0.301) | 0.000 |  | 0.142(0.067,0.300) | 0.000 |  | 0.142(0.067,0.301) | 0.000 |  |
| hct_group | Lower and decreasing | Ref |  |  | Ref |  |  | Ref |  |  | Ref |  |  | Ref |  |  |  | Ref |  |
|  | Lower and growing slightly | 0.618(0.427,0.895) | 0.011 |  | 0.615(0.425,0.889) | 0.010 |  | 0.615(0.425,0.889) | 0.010 |  | 0.557(0.387,0.801) | 0.011 |  | 0.617(0.426,0.893) | 0.010 |  | 0.616(0.426,0.892) | 0.010 |  |
|  | Higher and growing slightly | 0.164(0.097,0.278) | 0.000 |  | 0.162(0.096,0.274) | 0.000 |  | 0.162(0.096,0.274) | 0.000 |  | 0.156(0.093,0.264) | 0.000 |  | 0.164(0.097,0.277) | 0.000 |  | 0.163(0.096,0.276) | 0.000 |  |
|  | Higher and growing steadily | 0.144(0.064,0.324) | 0.000 |  | 0.143(0.064,0.321) | 0.000 |  | 0.143(0.064,0.321) | 0.000 |  | 0.137(0.061,0.307) | 0.000 |  | 0.144(0.064,0.323) | 0.000 |  | 0.144(0.064,0.323) | 0.000 |  |


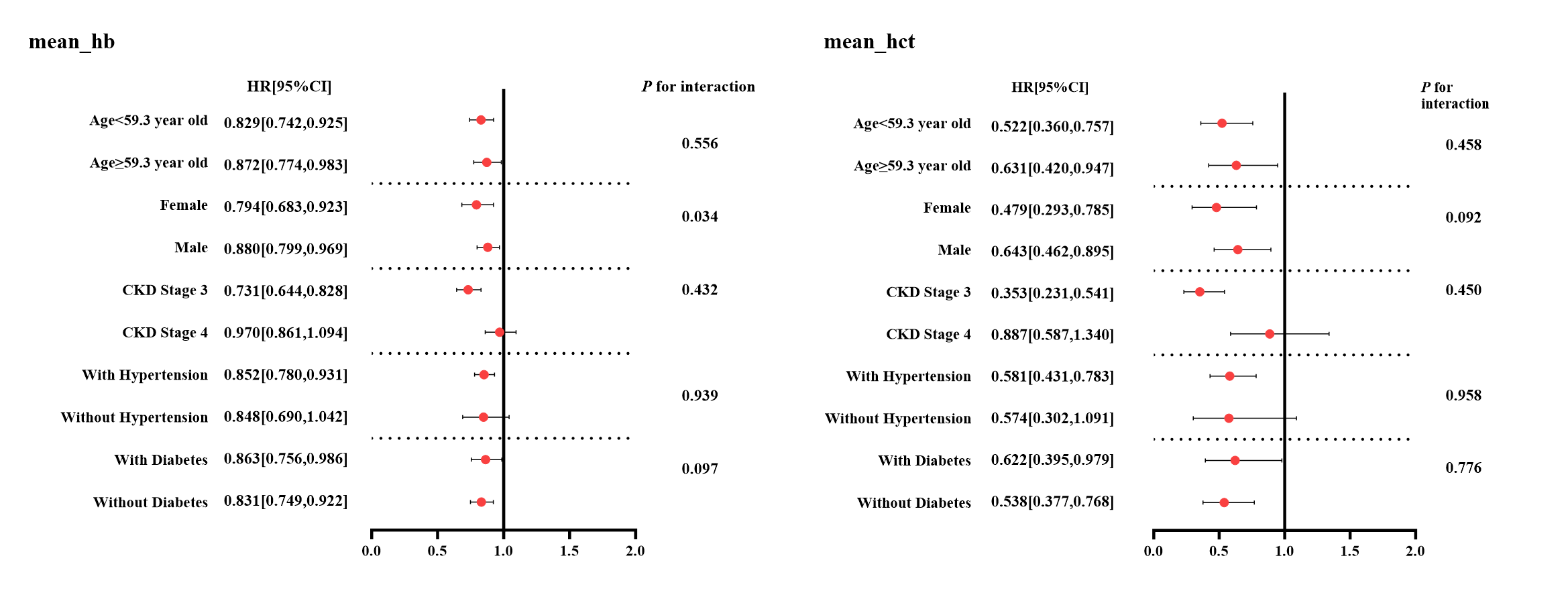


Figure1 Subgroup analyse of mean_hb and mean_hct


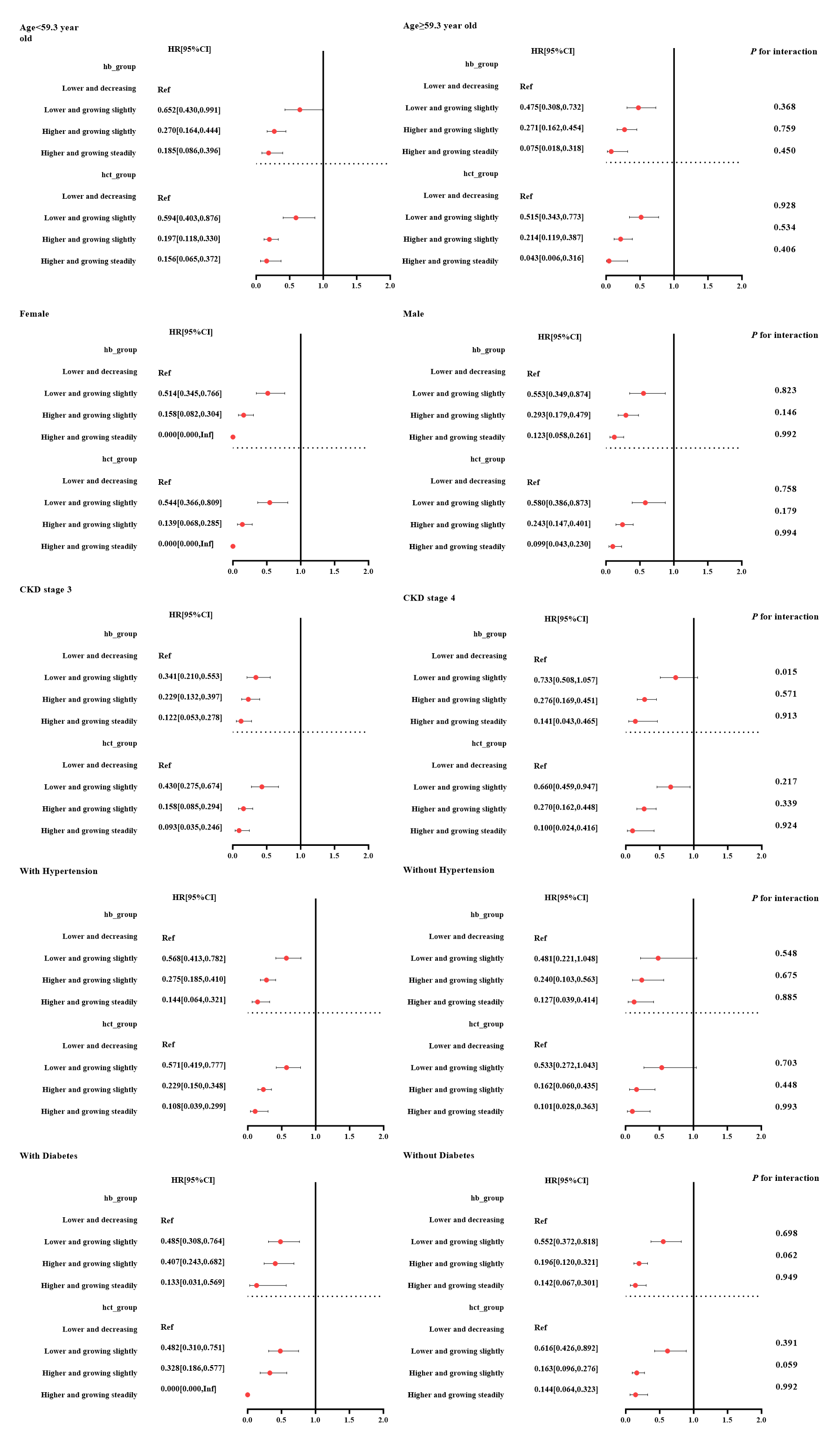


Figure2 Subgroup analyse of hb trajectories and hct trajectories
