# Supplementary material for: Time-updated patterns of hemoglobin and hematocrit and the risk of CKD progression
Source: Front Endocrinol (Lausanne). 2025 Oct 30;16:1642307. doi: 10.3389/fendo.2025.1642307 (PMC12611651; doi:10.3389/fendo.2025.1642307)
Supplement: Supplementary file 6 [file DataSheet6.docx]

**Supplementary file 6 Sensitivity analyse**

Supplementary file 6.1 Test for assumption and Univariate Cox regression of Sensitivity analyse

Table 1 Test for assumption, Univariate Cox regression of demographic information, clinical diagnosis and medication data

| Variables |  | HR(95%CI) | *P* for univariate Cox regression | *P* for Schoenfeld Individual Test |
| --- | --- | --- | --- | --- |
| Age, year |  | 0.997(0.986,1.008) | 0.637 | 0.145 |
| eGFR, ml/min/1.73 m^2^ |  | 0.942(0.930,0.955) | <0.000 | 0.515 |
| Sex | Female | Ref |  | 0.370 |
|  | Male | 0.996(0.724,1.369) | 0.978 |  |
| With Hypertension | No | Ref |  | 0.926 |
|  | Yes | 2.061(1.313,3.237) | 0.002 |  |
| With Diabetes | No | Ref |  | 0.045 |
|  | Yes | 1.955(1.426,2.679) | 0.000 |  |
| With Hyperuricemia | No | Ref |  | 0.305 |
|  | Yes | 1.228(0.751,2.007) | 0.414 |  |
| With Hyperlipidemia | No | Ref |  | 0.438 |
|  | Yes | 1.190(0.820,1.728) | 0.359 |  |
| With Anemia | No | Ref |  | 0.139 |
|  | Yes | 1.582(0.833,3.005) | 0.161 |  |
| Protopathy | Primary Glomerulonephritides | Ref |  | 0.535 |
|  | Hypertensive Renal Disease | 1.041(0.373,2.906) | 0.940 |  |
|  | Diabetic nephropathy | 3.093(1.710,5.594) | 0.000 |  |
|  | Others | 0.652(0.317,1.343) | 0.246 |  |
|  | Unknown | 1.216(0.839,1.763) | 0.303 |  |
| With ACEI/ARB | No | Ref |  | 0.619 |
|  | Yes | 0.928(0.677,1.272) | 0.643 |  |
| With Calcium Supplements | No | Ref |  | 0.170 |
|  | Yes | 0.823(0.567,1.195) | 0.306 |  |
| With Sodium Bicarbonate | No | Ref |  | 0.896 |
|  | Yes | 1.685(1.230,2.308) | 0.001 |  |
| With Ketoacid Tablets | No | Ref |  | 0.038 |
|  | Yes | 1.986(1.450,2.719) | 0.000 |  |
| With Diuretics | No | Ref |  | 0.009 |
|  | Yes | 1.365(0.969,1.922) | 0.075 |  |
| With ESAs or Iron | No | Ref |  |  |
|  | Yes | 1.776(1.239,2.545) | 0.002 | 0.832 |
| ALB, g/L |  | 0.972(0.955,0.990) | 0.002 | 0.056 |
| Urea, mmol/L |  | 1.078(1.057,1.100) | 0.000 | 0.096 |
| UA, mmol/L |  | 1.000(0.999,1.002) | 0.624 | 0.747 |
| TCO2, mmol/L |  | 0.927(0.883,0.974) | 0.002 | 0.023 |
| LDL-C, mmol/L |  | 1.032(0.957,1.113) | 0.415 | 0.562 |
| TC, mmol/L |  | 1.032(0.963,1.105) | 0.376 | 0.753 |
| HDL-C, mmol/L |  | 0.827(0.545,1.253) | 0.370 | 0.050 |
| AST, mmol/L |  | 1.004(0.992,1.016) | 0.527 | 0.067 |
| ALT, mmol/L |  | 0.997(0.988,1.007) | 0.571 | 0.915 |
| Exposures 1 |  |  |  |  |
| baseline_Hb, g/L |  | 0.978(0.972,0.985) | 0.000 | 0.602 |
| mean_Hb, g/L |  | 0.958(0.949,0.967) | <0.000 | 0.017 |
| Exposures 2 |  |  |  |  |
| baseline_HCT, % |  | 0.923(0.901,0.946) | 0.000 | 0.615 |
| mean_HCT, % |  | 0.860(0.834,0.887) | <0.000 | 0.012 |
| GBTM |  |  |  |  |
| hb_group | Lower and decreasing | Ref |  | 0.397 |
|  | Lower and growing slightly | 0.454(0.314,0.657) | 0.000 |  |
|  | Higher and growing slightly | 0.176(0.111,0.280) | 0.000 |  |
|  | Higher and growing steadily | 0.099(0.050,0.197) | 0.000 |  |
| hct_group | Lower and decreasing | Ref |  | 0.541 |
|  | Lower and growing slightly | 0.345(0.242,0.492) | 0.000 |  |
|  | Higher and growing slightly | 0.125(0.076,0.204) | <0.000 |  |
|  | Higher and growing steadily | 0.075(0.034,0.164) | 0.000 |  |

Note: angiotensin converting enzyme inhibitors, ACEI; Angiotensin receptor blocker, ARB;Primary Glomerulonephritides included chronic nephritis, nephropathy syndrome and IgA nephropathy.Other secondary nephrosis included systemic lupus erythematosus nephritis, Henoch-Schonlein purpura,Hepatitis B virus-associated nephritis and obstructive nephropathy, etc.;estimated glomerular filtration rate, eGFR; albumin, ALB; uric acid, UA; total carbon dioxide, TCO2; low-density lipoprotein cholesterol, LDL-C; total cholesterol, TC; high-density lipoprotein cholesterol, HDL-C; aspartate transaminase, AST; alanine aminotransferase, ALT; hemoglobin, Hb; hematocrit, HCT.

Supplementary file 6.2 Lasso selection

| Exposures 1 |  |
| --- | --- |
| 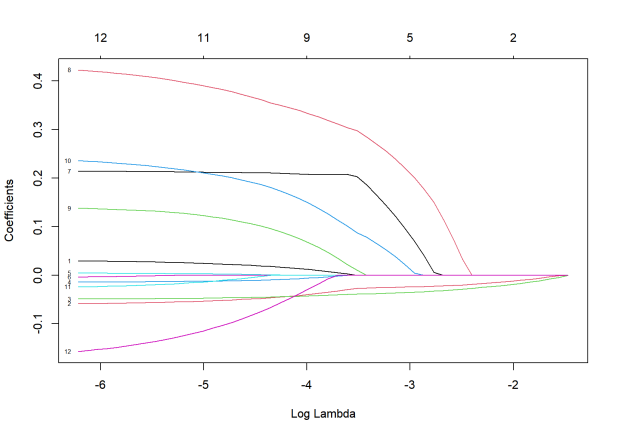 | 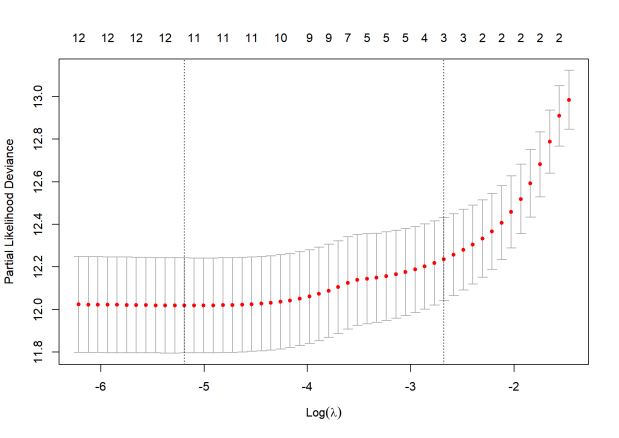 |
| Exposures 2 |  |
| 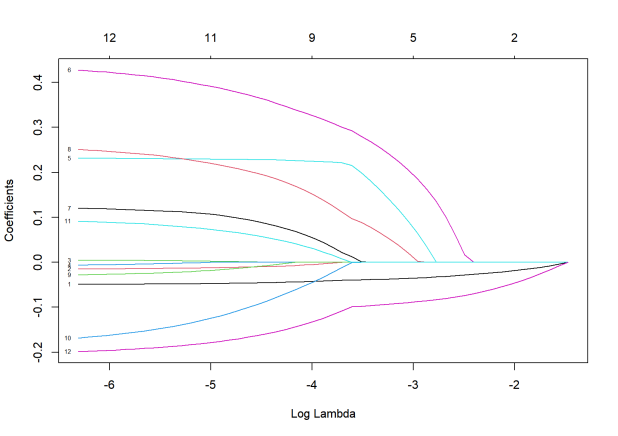 | 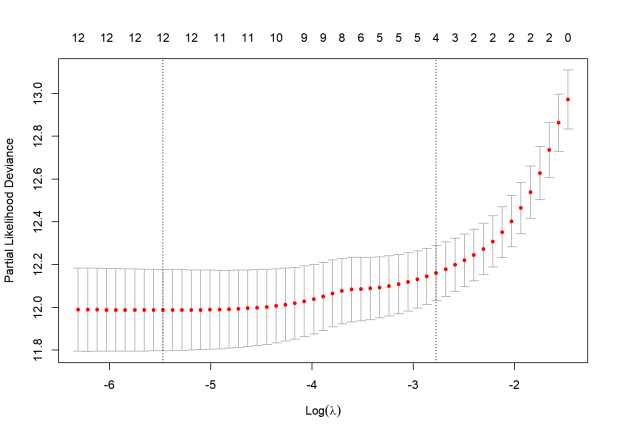 |
| hb_group |  |
| 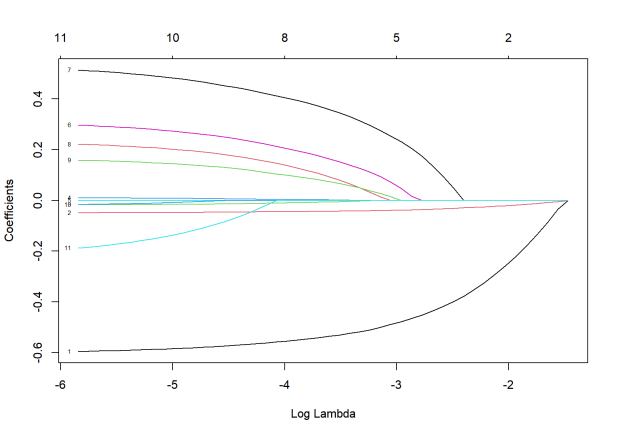 | 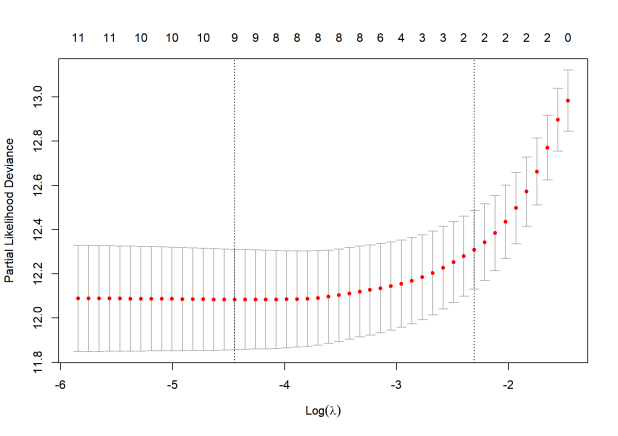 |
| hct_group |  |
| 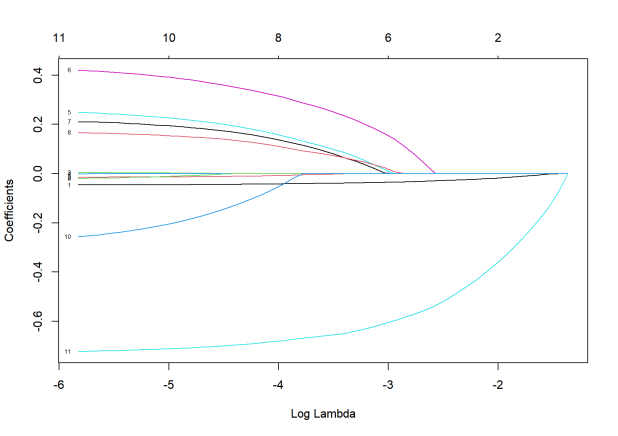 | 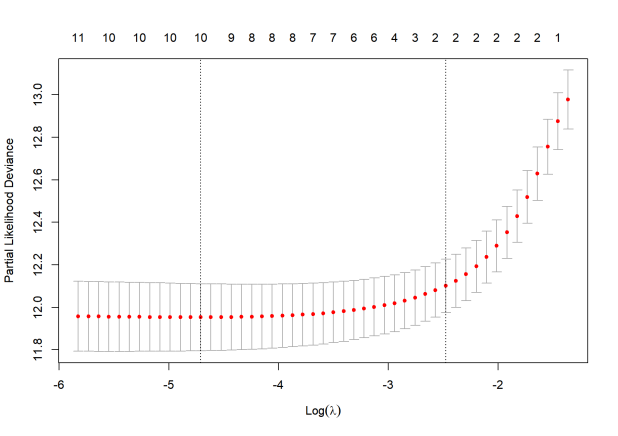 |

Figure1 Lasso selection of Exposures

Results of Multivariate Cox regression

Table 4 Multivariate Cox regression of Exposures

| Variables |  | HR(95%CI) | *P* | *P* for trend |
| --- | --- | --- | --- | --- |
| Exposures 1 |  |  |  |  |
| mean_hb, g/L |  | 0.862(0.782,0.950) | 0.003 |  |
| eGFR, ml/min/1.73 m^2^ |  | 0.954(0.941,0.967) | 0.000 |  |
| With Diabetes | No | Ref |  |  |
|  | Yes | 1.653(1.198,2.282) | 0.002 |  |
| Exposures 2 |  |  |  |  |
| mean_HCT, % |  | 0.580(0.416,0.808) | 0.001 |  |
| eGFR, ml/min/1.73 m^2^ |  | 0.953(0.941,0.966) | 0.000 |  |
| With Hypertension | No | Ref |  |  |
|  | Yes | 1.603(1.008,2.550) | 0.046 |  |
| With Diabetes | No | Ref |  |  |
|  | Yes | 1.503(1.080,2.089) | 0.015 |  |
| hb_group |  |  |  |  |
| hb_group | Lower and decreasing | Ref |  | 0.000 |
|  | Lower and growing slightly | 0.573(0.394,0.831) | 0.003 |  |
|  | Higher and growing slightly | 0.230(0.144,0.368) | 0.000 |  |
|  | Higher and growing steadily | 0.166(0.083,0.334) | 0.000 |  |
| eGFR, ml/min/1.73 m^2^ |  | 0.951(0.938,0.964) | 0.000 |  |
| hct_group |  |  |  |  |
| hct_group | Lower and decreasing | Ref |  | 0.000 |
|  | Lower and growing slightly | 0.460(0.321,0.660) | 0.000 |  |
|  | Higher and growing slightly | 0.173(0.105,0.285) | 0.000 |  |
|  | Higher and growing steadily | 0.121(0.055,0.266) | 0.000 |  |
| eGFR, ml/min/1.73 m^2^ |  | 0.955(0.942,0.968) | 0.000 |  |

Note: estimated glomerular filtration rate, eGFR;hemoglobin, Hb; hematocrit, HCT.
